# Biotransformation of Ursonic Acid by *Aspergillus ochraceus* and *Aspergillus oryzae* to Discover Anti-Neuroinflammatory Derivatives

**DOI:** 10.3390/molecules28247943

**Published:** 2023-12-05

**Authors:** Yan-Ni Wu, Dan Su, Jia Yang, Ying Yi, An-Dong Wang, Min Yang, Jian-Lin Li, Bo-Yi Fan, Guang-Tong Chen, Wen-Li Wang, Bai Ling

**Affiliations:** 1 School of Pharmacy, Nantong University, 19 Qixiu Road, Nantong 226001, Chinawangwenli16@126.com (W.-L.W.); 2 Department of Pharmacy, The Fourth Affiliated Hospital of Nantong University, The First People’s Hospital of Yancheng, 166 Yulongxi Road, Yancheng 224005, China

**Keywords:** ursonic acid, biotransformation, anti-neuroinflammatory, *Aspergillus ochraceus*, *Aspergillus oryzae*

## Abstract

Biotransformation of ursonic acid (**1**) by two fungal strains *Aspergillus ochraceus* CGMCC 3.5324 and *Aspergillus oryzae* CGMCC 3.407 yielded thirteen new compounds (**4**, **5**, **7**–**10**, and **13**–**19**), along with five recognized ones. The structural details of new compounds were determined through spectroscopic examination (NMR, IR, and HR-MS) and X-ray crystallography. Various modifications, including hydroxylation, epoxidation, lactonization, oxygen introduction, and transmethylation, were identified on the ursane core. Additionally, the anti-neuroinflammatory efficacy of these derivatives was assessed on BV-2 cells affected by lipopolysaccharides. It was observed that certain methoxylated and epoxylated derivatives (**10**, **16**, and **19**) showcased enhanced suppressive capabilities, boasting IC_50_ values of 8.2, 6.9, and 5.3 μM. Such ursonic acid derivatives might emerge as potential primary molecules in addressing neurodegenerative diseases.

## 1. Introduction

Neurodegenerative diseases (NDDs), including Alzheimer’s disease, Parkinson’s disease, Huntington’s disease, and multiple sclerosis, are an important global healthy problem due to an increase in the aging population [1]. It brings a huge burden to patients and social sanitary systems all over the world. However, the pathologies of these diseases are not fully understood and some factors, such as genetic factors, oxidative stress, neuroinflammation, and environmental factors, are believed to have played a role in the development of NDDs. In clinics, there is no effective cure for these diseases [2,3]. Therefore, it is a very challenging task to find innovative potential drugs for NDDs.

Ursonic acid (UNA, **1**), an ursane-type compound with a five-ring triterpene structure, is commonly found in a variety of plants frequently used in traditional remedies [4,5,6]. It exhibits an array of biological properties, encompassing anti-inflammatory, anti-cancer, growth-inhibitory, and anti-protozoan effects [7,8,9]. Notably, being a primary oxo-derivative of ursolic acid (ULA), UNA is a crucial chemical precursor for developing potential drug candidates. However, its medicinal potential and the mechanisms driving its effects remain relatively underexplored [10].

Various semisynthetic derivatives of UNA have been chemically crafted, showcasing enhanced absorption in the digestive tract and amplified medicinal properties [11,12,13]. Yet, these chemical methods have predominantly targeted only the activated substituents at C-3 and C-28 of the molecular framework. The potential for a wider range of structural variations of UNA is restrained due to its dearth of functional units. However, this limitation is difficult to overcome by conventional chemical synthesis.

Utilizing biotransformation emerges as a strategic method to attain structural variation, especially when dealing with intricate natural compounds [14,15,16,17]. The significant improvements in instruments and experimental techniques have enabled the biotransformation process to be carried out in NMR tubes and in situ monitoring using NMR spectroscopy [18,19]. Biotransformation offers a solution for targeting specific molecular sites that traditional chemical procedures may find challenging. In particular, microbial transformation stands out for its ability to provide precise and location-specific alterations in the structure of triterpenoids, thanks to its inherent stereo- and region-selective catalytic potential [20,21,22,23]. Even though numerous microbial adaptations of ULA have been explored to yield derivatives with augmented solubility and therapeutic traits, such endeavors with UNA are not as prevalent [24]. Hence, leveraging biotransformation to craft UNA derivatives is of immense significance.

Anti-neuroinflammatory effects of triterpenoids from medicinal plants are well reported [25]. Nitric oxide (NO) is a molecule which is highly linked with immunity and inflammation [26]. The anti-neuroinflammatory activity of some ULA derivatives has been evaluated with lipopolysaccharide (LPS)-induced BV-2 microglia [27]. As ongoing research to find triterpenoid derivatives with anti-neuroinflammatory activity, in this study, we identified 13 previously undescribed derivatives of UNA derived from the biotransformation processes of two fungal varieties: *Aspergillus ochraceus* CGMCC 3.5324 and *Aspergillus oryzae* CGMCC 3.407. Additionally, we evaluated the anti-neuroinflammatory properties of these biotransformation derivatives to inhibit LPS-induced NO production in BV-2 cells.

## 2. Results

### 2.1. Biotransformation of Ursonic Acid

**Figure 1:**
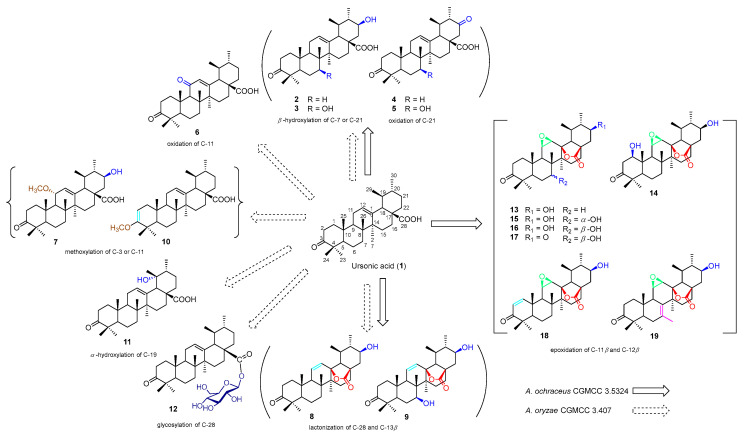
Biotransformation of ursonic acid (**1**) by *A. ochraceus* and *A. oryzae*.

We introduced 2.0 g of UNA to *A. ochraceus* cultures and a lesser quantity of 1.2 g to *A. oryzae* cultures. Following a week-long incubation period, both cultures were merged and subsequently sieved. We proceeded to extract the filtrates using ethyl acetate in three separate stages. After concentrating these extracts, we differentiated them through multiple rounds of column chromatography and semi-preparative high-performance liquid chromatography (HPLC). This process yielded a total of 18 distinct compounds (**2**–**19**). Specifically, *A. ochraceus* produced 13 of these derivatives (**2**–**5**, **8**, **9**, and **13**–**19**), while *A. oryzae* was responsible for the remaining 11 (**2**–**12**) (Figure 1).

To ascertain the molecular structures of these derivatives, we undertook a thorough examination utilizing diverse spectral studies and X-ray crystallography. Remarkably, among these, 13 (**4**, **5**, **7**–**10**, and **13**–**19**) were identified as novel compounds, as shown in Figure 1. Detailed ^1^H and ^13^C NMR data pertaining to these compounds are presented in Table 1, Table 2, Table 3 and Table 4. Furthermore, all relevant spectra can be found in the Supplementary Materials, labeled as Figures S1–S104. For compounds already known in the scientific literature, we determined their identities through spectral comparison. These were recognized as 3-oxo-21β-hydroxy-12-en-urs-28-oic acid (**2**) [28], 3-oxo-7β,21β-dihydroxy-12-en-28-oic acid (**3**) [29], 3,11-dioxo-12-en-urs-28-oic acid (**6**) [30], 3-oxo-19α-hydroxy-12-en-urs-28-oic acid (**11**), and ursonic acid -28-*O*-β-d-glucopyranosyl ester (**12**) [31] by juxtaposing their spectroscopic data with previously documented findings.

**Figure 2:**
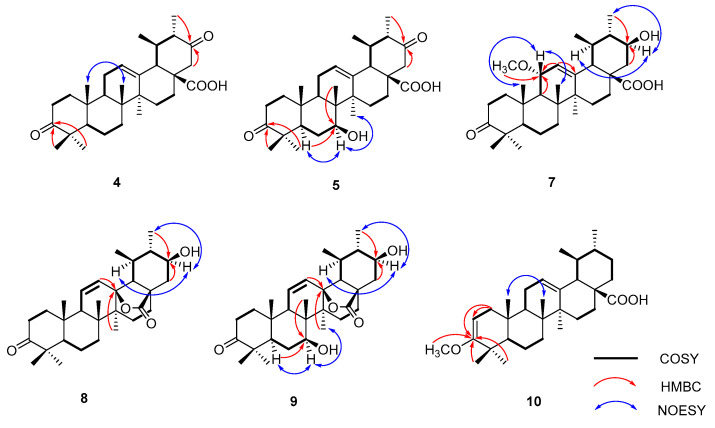
Key ^1^H-^1^H COSY, HMBC, and NOESY correlations for compounds **4**, **5**, and **7**–**10**.

Compound **4**, based on HR-ESI-MS measurements, displayed a molecular structure C_30_H_44_O_4_, as shown by the [M − H]^−^ ion at *m*/*z* 467.3174 (calcd. for C_30_H_43_O_4_, 467.3161). This structure was 14 amu heavier than UNA. The ^13^C NMR spectrum notably exhibited a fresh carbonyl signal at *δ*_C_ 209.8 ppm. In the HMBC spectrum, connections between H-22 (*δ*_H_ 2.57 and 2.35) and C-21 (*δ*_C_ 209.8) became evident (Figure 2). Also, correlations of 30-CH_3_ (*δ*_H_ 1.01) with the carbonyl signal at *δ*_C_ 209.8 were noticed. These data led to the identification of the carbonyl group at C-21, thus finalizing compound **4** as 3,21-dioxo-urs-12-en-28-oic acid.

For compound **5**, its HR-ESI-MS data revealed a molecular formula of C_30_H_44_O_5_, as reflected by the [M − H]^−^ ion at *m*/*z* 483.3126 (calcd. for C_30_H_43_O_5_, 483.3112). This was 30 amu heavier than UNA. The ^1^H NMR spectrum showed a new signal at *δ*_H_ 3.89, indicating the presence of a hydroxyl group. The ^13^C NMR spectrum distinguished itself with an oxygenated methine at *δ*_C_ 73.4 and a carbonyl signal at *δ*_C_ 210.4 ppm. HMBC correlations between 26-CH_3_ (*δ*_H_ 0.81) and the oxygenated methine at *δ*_C_ 73.4 suggested the attachment of the hydroxyl group to C-7 (Figure 2). The NOESY data further linked H-7 (*δ*_H_ 3.87) and 27-CH_3_ (*δ*_H_ 1.08), affirming the β-orientation of the 7-OH group. The HMBC spectrum showcased H-22 (*δ*_H_ 2.56 and 2.37) correlations with C-21 (*δ*_C_ 210.4). The linkage of 30-CH_3_ (*δ*_H_ 0.99) with the carbonyl signal at *δ*_C_ 210.4 confirmed the carbonyl’s placement at C-21. Hence, compound **5** was determined to be 3,21-dioxo-7β-hydroxy-urs-12-en-28-oic acid.

**Table 1:** The ^1^H- and ^13^C-NMR data of compounds **4**, **5**, and **7** (400 and 100 MHz, respectively, in CDCl_3_)

Position	4		5		7 ^a^	
	*δ*_C_	*δ*_H_ (*J* in Hz)	*δ*_C_	*δ*_H_ (*J* in Hz)	*δ*_C_	*δ*_H_ (*J* in Hz)
1	39.3	1.39, 1.83 m	39.1	1.35, 1.86 m	40.4	1.67, 2.22 m
2	34.1	2.30, 2.48 m	34.0	2.34, 2.48 m	34.4	2.39, 2.53 m
3	217.7		217.0		217.9	
4	47.4		47.7		47.6	
5	55.2	1.23 m	52.5	1.37 m	55.3	1.36 m
6	19.5	1.24, 1.41 m	26.6	1.55, 1.82 m	19.6	1.32, 1.47 m
7	32.3	1.29, 1.42 m	73.4	3.89 dd (9.7, 6.0)	33.1	1.31, 1.46 m
8	39.5		45.2		42.3	
9	46.7	1.53 m	47.0	1.44 m	51.3	1.74 d (9.1)
10	36.7		36.8		37.7	
11	23.6	1.53, 1.94 m	23.7	1.93, 2.04 m	76.5	3.84 d (8.6)
12	127.2	5.35 d (3.8)	127.5	5.42 dd (4.6, 2.7)	125.7	5.52 m
13	137.1		136.6		141.6	
14	41.9		43.4		42.3	
15	29.7	1.14 d (6.0), 1.18 m	31.6	1.49, 1.92 m	28.1	1.15, 1.77 m
16	27.9	1.06, 1.80 m	30.4	1.37, 1.54 m	25.2	1.77, 1.88 m
17	51.2		51.1		48.4	
18	52.4	2.62 m	51.1	2.08 m	51.8	2.29 d (11.4)
19	41.5	1.72 m	41.7	1.74 m	37.7	1.46 m
20	51.1	2.08 m	53.1	2.63 d (11.4)	46.6	0.99 m
21	209.8		210.4		71.1	3.44 m
22	50.5	2.35, 2.57 m	50.3	2.37 m, 2.56 d (12.9)	44.5	1.58, 2.09 m
23	26.6	1.02 s	26.6	1.04 s	26.7	1.09 s
24	21.5	0.96 s	21.5	0.98 s	21.5	1.04 s
25	15.3	0.99 s	12.4	1.00 s	15.7	1.13 s
26	17.0	0.76 s	9.7	0.81 s	18.5	0.86 s
27	23.8	0.99 s	23.4	1.08 s	22.7	1.15 s
28	180.0		179.1		175.7	
29	18.4	0.95 d (5.7)	18.4	0.95 d (6.4)	17.2	0.99 d (6.5)
30	12.5	1.01 d (3.7)	15.4	0.99 d (5.7)	16.5	1.09 d (6.3)
-OCH_3_					54.7	3.29 s

^a^ H and C were measured at 600 and 150 MHz, respectively.

Compound **7**, as determined by its HR-ESI-MS data, had a molecular formula of C_31_H_48_O_5_. This was supported by the [M − H]^−^ ion at *m*/*z* 499.3433 (calcd. for C_31_H_47_O_5_, 499.3423), indicating that it was 46 amu heavier than UNA. In the ^1^H NMR spectrum, two prominent signals at *δ*_H_ 3.84 and 3.44 emerged. Additionally, the ^13^C NMR and DEPT 135 spectra displayed new methine carbon signals at *δ*_C_ 76.5 and 71.1. The oxygenated methine signal at *δ*_C_ 71.1 was mapped to C-21, drawn from HMBC correlations between C-21 (*δ*_C_ 71.1) and 30-CH_3_ (*δ*_H_ 1.09) (Figure 2). The shift of C-30 to *δ*_C_ 16.5 was attributed to the γ-gauche effect. The ^1^H-^1^H COSY and HSQC spectra depicted a connectivity of H-9 (*δ*_H_ 1.74) → H-11 (*δ*_H_ 3.84) → H-12 (*δ*_H_ 5.52) within the C ring. This traced the oxygenated methine signal at *δ*_C_ 76.5 to C-11. A methoxyl singlet in the ^1^H NMR spectrum was evident at *δ*_H_ 3.29, with its carbon counterpart at *δ*_C_ 54.7 in the HSQC spectrum. In the HMBC spectrum, a clear connection between C-11 (*δ*_C_ 76.5) and the methoxyl singlet (*δ*_H_ 3.29) was observed. The 11-OCH_3_ group’s α-orientation was confirmed through NOESY correlations of H-11 (*δ*_H_ 3.84) and 26-CH_3_ (*δ*_H_ 0.86). Hence, compound **7** was finalized as 3-oxo-11α-methoxy-21β-hydroxy-urs-12-en-28-oic acid.

Compound **8**’s molecular formula was inferred to be C_30_H_44_O_4_ based on HR-ESI-MS data ([M − H_2_O + H]^+^ *m*/*z* 451.3216, calcd. for C_30_H_43_O_3_ 451.3212), suggesting a 14 amu difference when contrasted with UNA. An extra low-field proton was observed at *δ*_H_ 3.39 in the ^1^H NMR reading for **8**, with the corresponding carbon reading at *δ*_C_ 71.8 evident in the HSQC reading. The HMBC revealed clear connections between the distinct 30-CH_3_ (*δ*_H_ 1.01) and the new oxygenated methine reading at *δ*_C_ 71.8 (Figure 2). NOESY correlations between H-21 (*δ*_H_ 3.39) and H-19 (*δ*_H_ 1.82) confirmed the β-orientation of the 21-OH group. In the ^1^H NMR reading, two vinyl protons appeared at *δ*_H_ 5.92 and 5.51, linking with two sp^2^ methine readings at *δ*_C_ 133.3 and 128.8 in the HSQC reading. This double bond deviated from the one found in UNA. The ^1^H-^1^H COSY connections between H-11 (*δ*_H_ 5.92) and H-9 (*δ*_H_ 1.98) indicated a double-bond shift from C-12 (13) to C-11 (12). This was further verified by long-range connections of H-11 with C-8 (*δ*_C_ 42.0) and C-13 (*δ*_C_ 89.5) and H-12 with C-18 (*δ*_C_ 59.9) in the HMBC spectrum. Additionally, the oxygen-rich quaternary carbon at *δ*_C_ 89.5 was linked to C-13 due to its connections with H-11 and 27-CH_3_ (*δ*_H_ 1.08). The chemical positioning of C-18 also changed (downshifting from about *δ*_C_ 52.7 to *δ*_C_ 59.9). Analyzing the NMR findings, two potential compositions for this substance were proposed. The first had free 13-OH and 28-COOH. The second suggested a lactone bond between 13-OH and 28-COOH. With a molecular weight of 468, it adhered to the latter configuration. Consequently, compound **8** was identified as 3-oxo-21β-hydroxy-urs-11-en-13β,28β-lactone.

Compound **9**’s molecular formula was inferred to be C_30_H_44_O_5_ from its HR-ESI-MS results ([M + COOH]^−^ *m*/*z* 529.3171, calcd. for C_31_H_45_O_7_ 529.3165), a 16 amu increment from metabolite **8**, pointing to an extra oxygen atom. In the ^1^H NMR reading, two more low-field protons, *δ*_H_ 3.87 and 3.39, were present. The ^13^C NMR, DEPT 135, and HSQC results displayed two fresh oxygenated methine signals at *δ*_C_ 72.6 and 71.7. Contrasted with **8**’s NMR findings, the ^13^C NMR readings were largely aligned, excluding the B ring. HMBC correlations between the distinct 26-CH_3_ (*δ*_H_ 1.07) and the new oxygenated methine signal at *δ*_C_ 72.6 were evident (Figure 2). Furthermore, NOESY correlations between H-7 (*δ*_H_ 3.87) and 27-CH_3_ (*δ*_H_ 1.15) confirmed the β-orientation of the 7-OH group. Thus, compound **9** was identified as 3-oxo-7β,21β-dihydroxy-urs-11-en-13β,28β-lactone.

**Figure 3:**
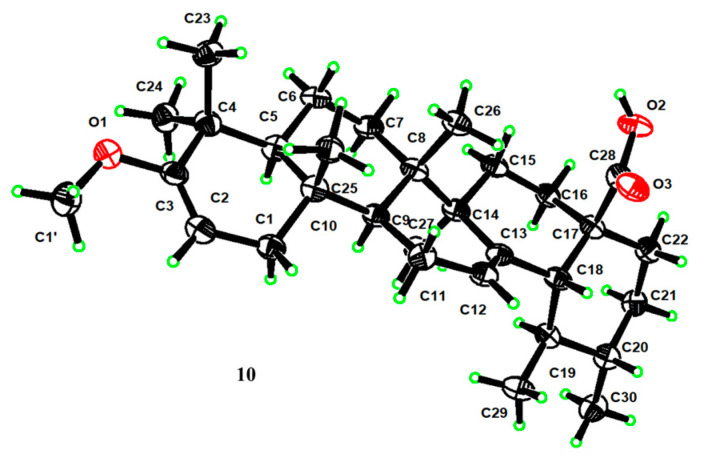
X-ray ORTEP drawing of compound **10**.

For compound **10**, its molecular formula was concluded to be C_31_H_48_O_3_ based on HR-ESI-MS, showing a [M − H]^−^ ion at *m*/*z* 467.3534 (calcd. for C_31_H_47_O_3_, 467.3525), a 14 amu increase from UNA. The ^1^H NMR reading for compound **10** displayed an extra vinyl proton at *δ*_H_ 4.39 and an oxygenated methine at *δ*_H_ 3.47. Their respective carbon signals at *δ*_C_ 89.6 and *δ*_C_ 54.2 appeared in the HSQC reading. The ^13^C NMR and DEPT 135 findings disclosed a new seasonal double-bond carbon signal at *δ*_C_ 160.6, and when compared to UNA’s NMR readings, metabolite **10**’s keto carbonyl signal at C-3 vanished. The carbon signal at *δ*_C_ 89.6 and the new seasonal double-bond carbon signal at *δ*_C_ 160.6 were likely paired due to the HMBC’s vinyl proton (*δ*_H_ 4.39) connections with the seasonal double-bond carbon signal at *δ*_C_ 160.6 (Figure 2). Moreover, the HMBC connections between 23-CH_3_ (*δ*_H_ 1.04), 24-CH_3_ (*δ*_H_ 0.93), and the new seasonal double-bond carbon signal (*δ*_C_ 160.6) hinted at the double bond’s positioning at C-2 and C-3. Additionally, the oxygenated methine group should connect to C-3 based on the HMBC correlation of C-3 (*δ*_C_ 160.6) with the oxygenated methine signal at *δ*_H_ 3.47. The 2D structure of compound **10** was further endorsed by suitable crystal X-ray crystallography [Cu Ka; Flack parameter: −0.4(5); CCDC: 2266165] (Figure 3). Sadly, ideal crystals were not acquired, making observation of absolute configurations impossible. Therefore, compound **10** was pinpointed as 3-methoxy-urs-2,12-dien-28-oic acid.

**Table 2:** The ^1^H- and ^13^C-NMR data of compounds **8**–**10** (400 and 100 MHz, respectively, in CDCl_3_).

Position	8		9		10	
	*δ*_C_	*δ*_H_ (*J* in Hz)	*δ*_C_	*δ*_H_ (*J* in Hz)	*δ*_C_	*δ*_H_ (*J* in Hz)
1	39.0	1.38, 2.02 m	38.5	1.34, 2.00 m	39.7	1.70, 2.00 m
2	33.9	2.36, 2.57 m	33.7	2.37, 2.55 m	89.6	4.39 dd (6.7, 1.8)
3	216.8		216.1		160.6	
4	47.6		47.2		37.1	
5	54.6	1.27 m	51.8	1.89 m	53.0	1.12 m
6	18.8	1.49, 1.62 m	29.8	1.52, 1.64 m	19.4	1.37, 1.48 m
7	30.5	1.22, 1.38 m	72.6	3.87 dd (9.9, 5.5)	32.4	1.37,1.51 m
8	42.0		46.8		39.4	
9	52.4	1.98 m	52.3	1.36 m	46.1	1.54 m
10	36.1		36.0		35.9	
11	133.3	5.92 dd (10.3, 1.6)	132.1	5.85 d (10.4)	23.3	1.91, 1.97 m
12	128.8	5.51 dd (10.3, 3.2)	129.5	5.53 dd (10.3, 3.1)	126.1	5.27 t (3.6)
13	89.5		89.4		137.7	
14	41.6		42.9		42.0	
15	25.6	1.17, 1.68 m	29.4	1.18, 1.91 m	28.0	1.11, 1.86 m
16	23.7	1.50 m, 1.92 td (13.0, 5.7)	23.8	1.48, 1.92 m	24.1	1.67, 2.00 m
17	45.6		45.5		48.1	
18	59.9	1.64 m	60.1	1.64 m	52.7	2.19 dd (11.5, 1.7)
19	36.0	1.82 m	36.0	1.84 m	39.1	1.34 m
20	47.8	0.78 m	47.8	0.80 m	38.8	1.01 m
21	71.8	3.39 m	71.7	3.39 m	30.7	1.34, 1.51 m
22	40.3	1.40 m, 2.09 dd (12.8, 4.5)	40.2	1.37 m, 2.09 dd (12.8, 4.5)	36.8	1.67, 1.71 m
23	26.0	1.03 s	26.0	1.04 s	28.6	1.04 s
24	20.8	0.98 s	20.8	0.98 s	20.0	0.93 s
25	17.8	0.99 s	17.1	0.98 s	15.4	0.95 s
26	18.6	1.03 s	13.7	1.07 s	17.0	0.80 s
27	16.0	1.08 s	16.3	1.15 s	23.5	1.08 s
28	178.5		178.5		183.7	
29	17.3	0.97 d (6.0)	17.8	0.97 d (5.8)	17.0	0.86 d (6.4)
30	14.4	1.01 d (6.1)	14.4	1.01 d (6.4)	21.2	0.94 d (5.4)
-OCH_3_					54.2	3.47 s

**Figure 4:**
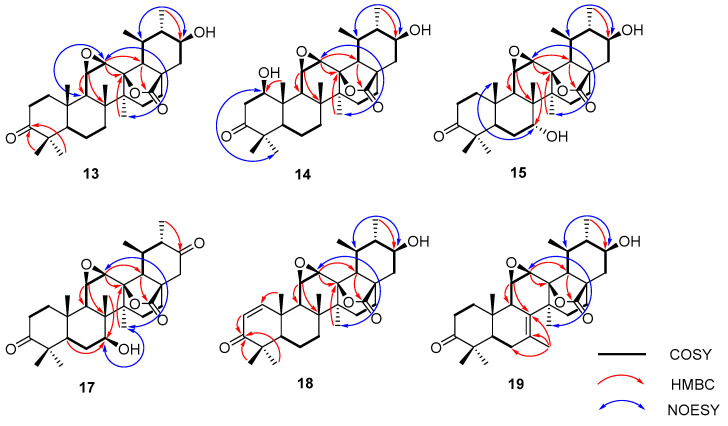
Key ^1^H-^1^H COSY, HMBC, and NOESY correlations for compounds **13**–**18** and **17**–**19**.

**Figure 5:**
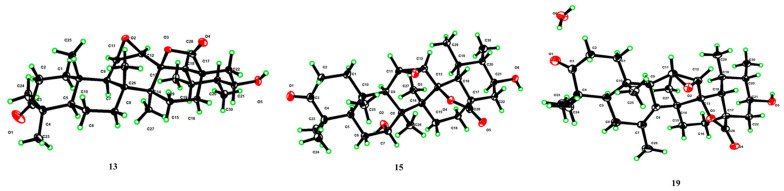
X-ray ORTEP drawing of compounds **13**, 1**5**, and **19**.

Compound **13**’s molecular formula was deduced as C_30_H_44_O_5_ from its HR-ESI-MS readings (*m*/*z* 529.3173 [M + COOH]^−^, computed for C_31_H_45_O_7_ at 529.3165). When juxtaposed with UNA (**1**) in the ^1^H NMR analysis, three distinct low-field protons surfaced at *δ*_H_ 3.16, 3.41, and 3.49. The ^13^C NMR and DEPT 135 analyses further identified three unique downfield methine carbon resonances at *δ*_C_ 51.3, 52.0, and 71.5. Key correlations were seen in the HMBC analysis, notably the 30-CH_3_ resonance (*δ*_H_ 1.13) associated with C-19 (*δ*_C_ 36.3), C-20 (*δ*_C_ 47.5), and an unfamiliar oxygenated methine resonance at *δ*_C_ 71.5 (Figure 4). Both the ^1^H-^1^H COSY and HSQC analyses showcased a spin system stretching from H-18 (*δ*_H_ 1.99) to H-22 (*δ*_H_ 1.51 and 2.21) in the E ring. These insights suggested the addition of a hydroxyl unit to C-21. The NOESY analysis underscored correlations between H-21 (*δ*_H_ 3.49) and H-19 (*δ*_H_ 1.97), indicating that the 21-OH unit possessed a β-orientation. Two proton resonances emerged in the ^1^H NMR reading at *δ*_H_ 3.41 and 3.16, linked to epoxide protons on C-11 and C-12. The ^13^C NMR reading aligned with the traits of the epoxy-γ-lactone component, evidenced by distinct peaks for C-11, C-12, and C-13 [32]. The HMBC reading (Figure 4) and other NMR analyses exposed a spin system in the C ring extending from H-9 (*δ*_H_ 1.62) to H-12 (*δ*_H_ 3.16). The NOESY study also identified associations between H-12 (*δ*_H_ 3.16) and 27-CH_3_ (*δ*_H_ 1.09), pinpointing a β-orientation for the C-11(12) epoxy unit (Figure 4). Our 1D and 2D NMR analyses conclusively disclosed the spatial arrangements across various ring junctions and specific orientations of functional groups. A conclusive configuration for **13** was substantiated through X-ray crystallographic evaluation [Cu Ka; Flack metric: 0.08(8); CCDC: 2256928] as (5*R*, 8*R*, 9*R*, 10*S*, 11*R*, 12*R*, 13*S*, 14*S*, 17*R*, 18*R*, 19*S*, 20*S*, 21*S*) (Figure 5). As a result, the identity of compound **13** was established as 3-oxo-21β-hydroxyl-11β,12β-epoxyl-urs-13β,28β-lactone.

**Table 3:** The ^1^H- and ^13^C-NMR data of compounds **13**–**15** (400 and 100 MHz, respectively, in CDCl_3_).

Position	13		14 ^a^		15 ^b^	
	*δ*_C_	*δ*_H_ (*J* in Hz)	*δ*_C_	*δ*_H_ (*J* in Hz)	*δ*_C_	*δ*_H_ (*J* in Hz)
1	39.6	1.62, 2.26 m	78.8	4.25 dd (7.2, 6.6)	39.1	1.67, 2.18 m
2	33.9	2.48, 2.71 m	45.8	2.99 dd (14.8, 6.6) 3.20 dd (14.8, 7.2)	33.6	2.41, 2.62 m
3	216.6		214.1		218.4	
4	47.7		47.5		46.9	
5	55.1	1.36 m	51.1	1.40 dd (12.6, 3.1)	46.3	2.02 m
6	19.0	1.56, 1.72 m	19.1	1.52, 1.71 m	28.8	1.32, 2.05 m
7	32.5	1.14, 1.39 m	32.3	1.03, 1.36 m	72.5	3.35 m
8	40.1		40.7		42.5	
9	49.2	1.62 m	50.2	1.98 br.s	44.9	2.14 m
10	37.3		43.9		37.3	
11	52.0	3.41 d (3.8)	54.8	4.78 d (3.8)	53.0	3.41 m
12	51.3	3.16 d (3.8)	52.1	3.28 d (3.8)	51.4	3.13 d (3.8)
13	88.8		89.2		90.2	
14	41.9		42.2		42.6	
15	25.5	1.20, 1.75 m	26.1	1.16, 1.79 m	24.4	1.60, 2.08 m
16	23.4	1.58, 1.97 m	23.9	1.55, 2.12 m	22.9	1.38, 2.08 m
17	46.3		46.6		46.2	
18	60.8	1.99 m	60.0	2.00 d (11.2)	60.9	1.85 dd (11.6, 1.4)
19	36.3	1.97 m	36.7	2.09 m	36.7	1.98 m
20	47.5	0.97 m	48.4	1.12 m	47.2	0.81 m
21	71.5	3.49 m	70.7	3.76 m	70.4	3.32 ddd (11.6, 10.0, 4.6)
22	40.2	1.51, 2.21 m	41.6	1.92, 2.56 m	40.0	1.32, 1.98 m
23	26.0	1.11 s	26.8	1.16 s	24.9	0.96 s
24	20.8	1.09 s	20.6	1.14 s	20.0	0.95 s
25	17.6	1.32 s	14.1	1.55 s	16.7	1.18 s
26	19.3	1.31 s	19.8	1.59 s	20.2	1.10 s
27	15.9	1.09 s	16.0	1.19 s	16.7	1.45 s
28	177.7		178.5		179.6	
29	18.6	1.20 d (5.0)	18.6	1.13 d (6.2)	17.7	1.11 d (5.7)
30	14.6	1.13 d (6.3)	15.0	1.33 d (6.4)	13.5	0.99 d (6.4)

^a^ H and C were measured at 600 and 150 MHz, respectively, in Pyridine-*d*_5_. ^b^ H and C were measured in CD_3_OD.

The molecular composition of compound **14** was confirmed as C_30_H_44_O_6_, supported by HR-ESI-MS readings (*m*/*z* 545.3156 [M + COOH]^−^, estimated for C_31_H_45_O_8_ at 545.3154), marking an increase of 16 amu from compound **13**. In the ^1^H NMR analysis, a distinct resonance at *δ*_H_ 4.25 (1H, dd, *J* = 7.2, 6.6 Hz) was seen, along with a corresponding oxygenated methine resonance at *δ*_C_ 78.8 in the ^13^C NMR reading. Hence, compound **14** was inferred to be a hydroxyl derivative of **13**. Within the HMBC analysis, crucial associations were observed between the 25-CH_3_ resonance (*δ*_H_ 1.55) and multiple carbon resonances, including the newly observed *δ*_C_ 78.8 (Figure 4). The NOESY study revealed correlations between H-1 (*δ*_H_ 4.25) and both H-5 (*δ*_H_ 1.40) and H-9 (*δ*_H_ 1.98), suggesting a β-orientation for the 1-OH group (Figure 4). Using analyses mirroring the NMR data of **13**, the epoxy-γ-lactone component was ascribed to positions C-11 through C-13 and C-28. The NOESY study further highlighted associations between H-12 (*δ*_H_ 3.28) and 27-CH_3_ (*δ*_H_ 1.19), pinpointing a β-orientation for the C-11(12) epoxy segment (Figure 4). Consequently, compound **14**’s identity was resolved as 3-oxo-1β,21β-dihydroxyl-11β,12β- epoxyl-urs-13β,28β-lactone.

For compound **15**, its molecular configuration was discerned as C_30_H_44_O_6_ from the HR-ESI-MS measurements (*m*/*z* 545.3130 [M + COOH]^−^, estimated for C_31_H_45_O_8_ at 545.3114). The ^1^H NMR analysis exhibited four distinct low-field proton resonances, and the ^13^C NMR displayed four unique downfield carbon resonances. Key associations in the HMBC were seen between the resonance of C-7 (*δ*_C_ 72.5) and 26-CH_3_ (*δ*_H_ 1.10) (Figure 4). NOESY associations of H-7 (*δ*_H_ 3.35) with both 25-CH_3_ (*δ*_H_ 1.18) and 26-CH_3_ (*δ*_H_ 1.10) proposed an α-orientation for the 7-OH segment (Figure 4). The oxygenated methine resonance at *δ*_C_ 70.4 was ascribed to C-21 based on HMBC associations with various resonances (Figure 4). Moreover, the NOESY study demonstrated links between H-21 (*δ*_H_ 3.32) and H-19 (*δ*_H_ 1.98), suggesting a β-orientation for the 21-OH segment (Figure 4). Employing assessments akin to the NMR data of **13**, the epoxy-γ-lactone segment was positioned at C-11 through C-13 and C-28. Subsequent NOESY associations between H-12 (*δ*_H_ 3.13) and 27-CH_3_ (*δ*_H_ 1.45) indicated a β-orientation for the C-11(12) epoxy fragment (Figure 4). After thorough validation using X-ray crystallography [Cu Ka; Flack value: 0.04(6); CCDC: 2256926] (Figure 5), compound **15**’s structure was firmly established as 3-oxo-7α,21β-dihydroxyl-11β,12β-epoxyl-urs-13β,28β-lactone.

Compound **16** displayed an [M + COOH]^−^ at *m*/*z* 545.3123 (calcd. for C_31_H_45_O_8_, 545.3114) signifying a molecular composition of C_30_H_44_O_6_. Compound **16** possessed two more hydroxylation sites at C-7 and C-21. One hydroxyl placement was determined at C-7 through the HMBC connections of 26-CH_3_ (*δ*_H_ 1.28) to C-7 (*δ*_C_ 73.7). Also, the NOESY interaction of H-7 (*δ*_H_ 3.79) with 27-CH_3_ (*δ*_H_ 1.20) indicated the β-orientation of the 7-OH. A second hydroxyl spot was discerned at C-21 due to the HMBC interactions of the distinctive 30-CH_3_ resonance (*δ*_H_ 1.08) with C-19 (*δ*_C_ 37.6), C-20 (*δ*_C_ 48.6), and a freshly oxygenated methine signal at *δ*_C_ 71.9. The upfield shift of C-30 to *δ*_C_ 14.7, attributed to the γ-gauche effect, substantiated the hydroxylation’s position at C-21. Moreover, the NOESY connections of H-21 (*δ*_H_ 3.42) to H-19 (*δ*_H_ 2.08) highlighted that the 21-OH group had a β-orientation. Relative to the NMR spectra of **13**, the epoxy-γ-lactone segment was attributed to C-11, C-12, C-13, and C-28. Hence, compound **16** was pinpointed as 3-oxo-7β,21β-dihydroxyl-11β,12β-epoxyl-urs-13β,28β-lactone.

For compound **17**, the molecular composition was delineated as C_30_H_42_O_6_ by HR-ESI-MS, noting an [M + COOH]^−^ ion at *m*/*z* 543.2968 (calcd. for C_31_H_43_O_8_, 543.2958), showing a 2 amu mass reduction in comparison to **16**, pointing to a dehydrogenated variant of compound **16**. The ^13^C NMR reading of compound **17** displayed an oxymethine at *δ*_C_ 73.0 and an extra carbonyl frequency at *δ*_C_ 208.4, implying that compound **17** emerged as a carbonylated variant of **16**. The carbonyl cluster was linked to C-21 owing to the HMBC interaction between C-21 (*δ*_C_ 208.4) and 30-CH_3_ (*δ*_H_ 1.09). Moreover, the NOESY interplay of H-7 (*δ*_H_ 3.78) with 27-CH_3_ (*δ*_H_ 1.14) indicated the β-orientation of the 7-OH. Consequently, compound **17** was pinpointed as 3,21-dioxo-7β-hydroxyl-11β,12β-epoxyl-urs-13β,28β-lactone.

Compound **18** displayed an [M + COOH]^−^ at *m*/*z* 527.2993 (calcd. for C_31_H_43_O_7_, 527.3001), representing a molecular composition of C_30_H_42_O_5_, which was 18 amu less than compound **14**. In the ^1^H NMR spectrum, two distinctive signals emerged at *δ*_H_ 7.62 (1H, d, *J* = 10.3 Hz) and *δ*_H_ 5.89 (1H, d, *J* = 10.3 Hz), with associated olefin carbon signals at *δ*_C_ 160.5 and *δ*_C_ 126.1 in the ^13^C NMR spectrum. Therefore, compound **18** appeared to be a desiccated variant of compound **14**. The HMBC spectrum revealed links of the distinct 25-CH_3_ resonance (*δ*_H_ 1.41) with C-5 (*δ*_C_ 54.5), C-9 (*δ*_C_ 45.1), C-10 (*δ*_C_ 41.4), and a fresh olefin carbon frequency at *δ*_C_ 160.5 (Figure 4). Furthermore, the carbonyl carbon signal at C-3 moved upfield to *δ*_C_ 207.3 due to the π → π conjugate effect, suggesting the presence of a double bond between C-1 and C-2. In the HMBC spectrum, connections of the signature 30-CH_3_ resonance (*δ*_H_ 1.09) with C-19 (*δ*_C_ 37.4), C-20 (*δ*_C_ 48.6), and a novel oxygenated methine signal at *δ*_C_ 71.8 were noted (Figure 4). Moreover, the NOESY interactions of H-21 (*δ*_H_ 3.42) with H-19 (*δ*_H_ 2.05) underscored that the 21-OH group had a β-orientation (Figure 4). By comparing with the NMR spectra of **13**, the epoxy-γ-lactone segment was pinpointed at C-11, C-12, C-13, and C-28. The NOESY interactions of H-12 (*δ*_H_ 3.28) with 27-CH_3_ (*δ*_H_ 1.14) revealed the β-orientation of the C-11(12) epoxy group. Thus, compound **18** was identified as 3-oxo-21β-hydroxyl-11β,12β-epoxyl-urs-1-ene-13β,28β-lactone.

The molecular structure of compound **19** was deduced as C_30_H_42_O_5_, as evidenced by the HR-ESI-MS displaying an [M + COOH]^−^ ion at *m*/*z* 527.3017 (calcd. for C_31_H_43_O_7_, 527.3017). Three additional low-field proton signals were spotted at *δ*_H_ 3.09, 3.30, and 3.42 in the ^1^H NMR spectrum. Furthermore, in the ^13^C NMR and DEPT 135 spectra, three more downfield methine carbon signals at *δ*_C_ 52.5, 52.6, and 71.7, along with two double-bond quaternary carbon signals at *δ*_C_ 128.5 and 129.7, were identified. The HMBC spectrum showcased correlations of the signature 30-CH_3_ resonance (*δ*_H_ 1.05) with C-19 (*δ*_C_ 35.3), C-20 (*δ*_C_ 47.5), and a new oxygenated methine signal at *δ*_C_ 71.7 (Figure 4). The NOESY interactions of H-21 (*δ*_H_ 3.42) with H-19 (*δ*_H_ 1.88) highlighted the β-orientation of the 21-OH group (Figure 4). The HMBC links of C-8 (*δ*_C_ 128.5) and C-7 (*δ*_C_ 129.7) with a methyl signal at *δ*_H_ 1.82 and of H-11 (*δ*_H_ 3.30) with C-8 (*δ*_C_ 128.5) and C-9 (*δ*_C_ 49.5) proposed a double bond at C-7(8) with the methyl group positioned at C-7. Using an analysis analogous to the NMR spectra of **13**, the epoxy-γ-lactone portion was mapped at C-11, C-12, C-13, and C-28. Additionally, the NOESY interactions of H-12 (*δ*_H_ 3.09) with 27-CH_3_ (*δ*_H_ 1.18) signified a β-orientation for the C-11(12) epoxy group. A subsequent X-ray crystallographic assessment [Cu Ka; Flack parameter: −0.09(10); CCDC: 2256927] (Figure 5) corroborated both the structure and definitive configuration of **19**. Hence, compound **19**’s structure was established as (5R, 9R, 10S, 11R, 12R, 13S, 14S, 17R, 18R, 19S, 20S, 21S) 3-oxo-21β-hydroxyl-7-methyl-11β,12β-epoxyl-7-ene-26-norurs-13β,28β-lactone.

**Table 4:** The ^1^H- and ^13^C-NMR data of compounds **16–19** (500 and 125 MHz, respectively, in CD_3_OD).

Position	16		17 ^a^		18		19 ^a^	
	*δ*_C_	*δ*_H_ (*J* in Hz)	*δ*_C_	*δ*_H_ (*J* in Hz)	*δ*_C_	*δ*_H_ (*J* in Hz)	*δ*_C_	*δ*_H_ (*J* in Hz)
1	40.0	1.70, 2.19 m	39.2	1.58, 2.23 m	160.5	7.62 d (10.3)	39.4	1.54, 2.27 m
2	34.8	2.51, 2.67 m	33.7	2.49, 2.69 m	126.1	5.89 d (10.3)	34.4	2.24 m 2.75 td (14.1, 5.5)
3	219.2		215.6		207.3		216.1	
4	48.3		47.3		46.1		47.1	
5	53.3	1.60 m	52.7	1.45 m	54.5	1.76 m	50.2	1.36 dd (12.5, 4.1)
6	30.8	1.62, 1.77 m	30.3	1.46, 1.70 m	19.5	1.63, 1.76 m	32.5	1.58, 2.61 m
7	73.7	3.79 dd (10.7, 4.3)	73.0	3.78 t (7.6)	33.6	1.17, 1.50 m	129.7	
8	46.7		45.4		42.3		128.5	
9	49.1	1.60 m	48.1	1.46 m	45.1	1.98 m	49.5	2.37 m
10	38.4		37.2		41.4		36.6	
11	53.9	3.44 m	52.3	3.43 m	52.8	3.75 d (3.8)	52.6	3.30 dd (3.8, 1.5)
12	53.0	3.19 d (3.9)	51.4	3.24 d (3.8)	52.8	3.28 d (3.8)	52.5	3.09 d (3.8)
13	91.3		88.7		90.9		87.8	
14	44.4		43.1		43.3		43.4	
15	30.4	1.29, 1.88 m	29.2	1.70, 1.96 m	26.7	1.28, 1.64 m	35.5	1.70, 2.14 m
16	24.6	1.41, 2.08 m	23.9	1.58, 1.70 m	24.4	1.43 m 2.14 td (13.3, 6.0)	23.7	1.50, 1.88 m
17	47.6		48.4		47.7		46.6	
18	62.4	1.99 d (11.7)	60.6	2.49 m	62.0	2.04 m	59.5	1.91 m
19	37.6	2.08 m	38.3	2.28 m	37.4	2.05 m	35.3	1.88 m
20	48.6	0.92 m	51.0	2.04 m	48.6	0.92 d	47.5	0.90 m
21	71.9	3.42 m	208.4	-	71.8	3.42 m	71.7	3.42 m
22	41.1	1.41, 2.06 m	47.0	2.58, 2.61 m	41.1	1.44, 2.06 m	39.8	1.44, 2.13 m
23	26.6	1.10 s	26.0	1.11 s	27.6	1.15 s	25.3	1.01 s
24	21.2	1.07 s	20.8	1.08 s	21.8	1.11 s	22.3	1.07 s
25	18.1	1.25 s	17.4	1.30 s	21.5	1.41 s	15.6	1.19 s
26	14.9	1.28 s	13.9	1.36 s	20.4	1.32 s	23.2	1.82 s
27	16.4	1.20 s	16.4	1.14 s	16.4	1.14 s	24.3	1.18 s
28	181.0		176.3		180.7		177.7	
29	18.9	1.19 d (6.3)	19.6	1.29 d (6.3)	18.9	1.21 d (5.6)	18.4	1.11 d (5.8)
30	14.7	1.08 d (6.6)	11.2	1.09 d (6.5)	14.8	1.09 d (6.4)	14.6	1.05 d (6.4)

^a^ H and C were measured at 400 and 100 MHz, respectively, in CDCl_3_.

### 2.2. Anti-Neuroinflammatory Activities

To assess the potential capabilities of all modified products in counteracting neuroinflammation, we measured their suppressive effects on NO generation within LPS-triggered BV-2 cells using the Griess method. Table 5 illustrates the findings. The majority of these modified substances exhibited stronger suppression capabilities on NO generation compared to the base compound, UNA. Specifically, compounds **7**, **10**, **13**, **16**, **18**, and **19** had notable suppression outcomes with IC_50_ values of 11.58, 8.23, 15.19, 6.86, 17.42, and 5.25 µM, respectively, surpassing the base compound’s IC_50_ at 84.72 µM. In addition, compounds **2**, **3**, **12**, **14**, and **15** exhibited intermediate suppression results with IC_50_ values of 38.17, 20.93, 31.05, 22.57, and 36.64 µM, respectively. Conversely, compounds **4**, **8**, and **9** did not showcase any suppression capabilities towards NO generation.

**Table 5:** Inhibitory effects of transformed products on NO production induced by LPS in BV-2 cells (mean ± SD, n = 3).

Compounds	IC_50_ (μM)	Cell Viability (%)	Compounds	IC_50_ (μM)	Cell Viability (%)
L-NMMA ^a^	28.25 ± 2.97	100.51 ± 4.36	**10**	8.23 ± 2.61	104.41 ± 4.05
Ursonic acid (**1**)	84.72 ± 3.22	98.84 ± 3.61	**11**	42.48 ± 3.70	101.29 ± 3.42
**2**	38.17 ± 4.09	101.25 ± 2.45	**12**	31.05 ± 3.98	98.23 ± 4.37
**3**	20.93 ± 2.13	100.33 ± 3.92	**13**	15.19 ± 3.07	102.64 ± 3.59
**4**	>100	103.62 ± 3.18	**14**	22.57 ± 3.44	104.05 ± 4.92
**5**	52.81 ± 3.34	100.56 ± 4.11	**15**	36.64 ± 2.33	101.78 ± 3.18
**6**	50.24 ± 3.16	104.79 ± 4.23	**16**	6.86 ± 3.52	97.82 ± 4.75
**7**	11.58 ± 2.01	99.17 ± 3.72	**17**	40.79 ± 3.26	100.37 ± 3.54
**8**	>100	102.48 ± 3.54	**18**	17.42 ± 2.72	103.24 ± 3.26
**9**	>100	103.95 ± 3.86	**19**	5.25 ± 3.19	102.83 ± 3.03

^a^ L-NMMA as a positive control.

## 3. Discussion

Earlier research on triterpenes reveals that microbial modifications possess a heightened catalytic propensity, resulting in a variety of hydroxylated and carbonylated byproducts [33]. *A. ochraceus* is prevalently found in the environment, commonly in soil and decaying plant matter. Historically, *A. ochraceus* has been employed as a biocatalyst in the hydroxylation processes of steroids, triterpenes, flavonoids, and coumarins [34,35,36,37]. In our present investigation, we discerned that *A. ochraceus* primarily initiated hydroxylation, oxidation, lactonization, and epoxidation reactions on UNA.

*A. ochraceus* exhibited the ability to concurrently initiate hydroxylation, lactonization, and epoxidation processes on UNA. In our current study, we isolated seven unique compounds (**13**–**19**) that simultaneously possessed the 21β-hydroxyl group, 11β,12β-epoxyl group, and 13β,28β-lactone. Notably, *A. ochraceus* exhibited a preference for initiating the 11β,12β-epoxidation, yielding compounds **13**–**19**, which are unveiled here for the first time. Furthermore, *A. ochraceus* triggered a transmethylation process, resulting in the formation of the distinctive ursane structure **19**. This process showcased an atypical biocatalytic transformation.

*A. oryzae * is predominantly identified in specific regions within China and Japan and is integral to the fermentation process of certain edibles. Its utility as a biocatalyst in the hydroxylation, oxidation, and lactonization of isoflavones, triterpenes, and sterols is well-documented [38,39,40,41]. In earlier findings, we have ascertained that *A. oryzae* can facilitate hydroxylation, acetylation, and epoxidation processes on cycloastragenol, a distinct cycloartane-type triterpene [42]. In this study, *A. oryzae* predominantly initiated 7β,21β-hydroxylation (**2**, **3**, and **6**–**9**), 21-oxidation (**4** and **5**), and 13β,28β-lactonization (**8** and **9**) reactions on UNA. Intriguingly, *A. oryzae* also showcased its ability to drive a methoxylation process either at C-3 or C-11, resulting in compounds **7** and **10**.

**Figure 6:**
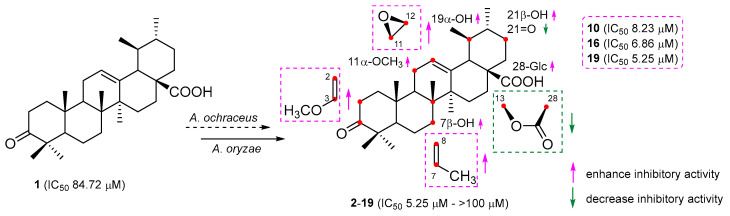
Preliminary structure–activity relationship of biotransformation products.

The position and arrangement of hydroxyl and epoxyl groups on the UNA structure can influence their capacity to inhibit NO activity. Compound **2**, which had a hydroxyl group at C-21β, demonstrated stronger inhibitory effects on NO generation compared to UNA itself. This finding indicated that introducing a hydroxyl group at C-21β could amplify the compound’s inhibitory effect on NO production. On the contrary, compound **4**, containing a carbonyl group at C-21, presented a notably reduced inhibition compared to compound **2**, indicating the detrimental effect of carbonylation at C-21. Moreover, compounds **3** and **5**, which had a hydroxyl group at C-7β, presented more potent inhibitory effects than that of compounds **2** and **4**, respectively. These indicated that hydroxylation at C-7β could enhance the NO inhibitory activity. In a parallel fashion, the inhibitory impact on NO production by compounds **13**–**19**, which possessed an epoxyl group at C-11β and C-12β, surpassed that of compounds **8** and **9**. This finding pointed to the conclusion that epoxidation at C-11β and C-12β could significantly augment inhibitory effects on NO production. Meanwhile, compounds **8** and **9**, carrying a lactone group at C-13β and C-28, did not exhibit any inhibitory activities, insinuating that having a lactone group at these positions could be detrimental to inhibiting NO production. Compounds **10**, **16**, and **19** showcased the strongest inhibitory potential, with IC_50_ values of 8.23, 6.86, and 5.25 µM, respectively (Figure 6). Such results underlined the promise of these compounds as primary candidates for addressing neuronal injuries.

The biotransformation of UNA by *A. ochraceus CGMCC 3.5324 and A. oryzae* CGMCC 3.407 produced 18 derivatives, of which 13 were novel compounds (**4**, **5**, **7**–**10**, and **13**–**19**). The principal reaction types observed were region-selective hydroxylation, epoxidation, lactonization, carbonylation, and transmethylation. Notably, *A. ochraceus* demonstrated the capability to concurrently catalyze the epoxidation at C-11(12) and the lactonization at C-13(28). Additionally, the epoxidation and lactonization reactions were stereo-selective at C-11β, C-12β, and C-13β positions. Achieving such specific reactions through conventional chemical synthesis is challenging. On the other hand, *A. oryzae* facilitated hydroxylation, oxidation, and lactonization reactions and uniquely catalyzed the methoxylation reaction, resulting in two distinct products. Some of these biotransformed derivatives exhibited significant inhibitory effects on NO production, positioning them as potential anti-neuroinflammatory agents. This research underscored the potential of biotransformation for the structural diversification of UNA, enabling the discovery of valuable derivatives. With the distinct biocatalytic capabilities of the fungi studied, a combination of microbial transformation and chemical semi-synthesis could be leveraged to produce an even broader array of UNA derivatives and analogs.

## 4. Materials and Methods

### 4.1. General

NMR spectra were recorded using Bruker AV-400, DRX-500, and AV-600 spectrometers. X-ray crystallographic analysis was conducted on a Bruker APEX-II CCD detector, utilizing graphite-monochromated Cu Kα radiation (λ = 1.54178 Å) from Bruker Biospin, Rheinstetten, Germany. Optical rotations were determined with a JASCO P-1020 digital polarimeter. Melting points, taken with an XT4A apparatus (Dianguang Corp., Shanghai, China), were uncorrected. IR spectra were obtained using a Nicolet 5700 FT-IR microscope spectrometer (Thermo Fisher Scientific Inc., Waltham, MA, USA). HR-ESI-MS spectral data were sourced from an Agilent 6540 UHD Q-TOF mass spectrometer. Reversed-phase preparative HPLC was conducted on a Shimadzu LC-20A instrument equipped with an SPD-20A UV detector and using YMC-Pack ODS-A (5 μm, 10.0 × 250 mm) columns.

### 4.2. Microorganism and Substance

UNA (**1**) was procured from Push Bio-technology Co., Ltd., Chengdu, China. Its authenticity was confirmed by comparing its physical and spectroscopic data with previously reported values. The purity was verified to be above 98% through UV-HPLC analysis. All solvents used were of AR grade, sourced from Sinopharm Chemical Reagent Co., Ltd., Shanghai, China. The strains *A. ochraceus* CGMCC 3.5324 and *A. oryzae* CGMCC 3.407 were acquired from the China General Microbiological Culture Collection Center (CGMCC). They were maintained on potato slants solidified with agar and stored at 4 °C. The BV-2 cell line was sourced from the Cell Bank of the Chinese Academy of Sciences.

### 4.3. Biotransformation Procedures

Biotransformation experiments were conducted in 1000 mL flasks, each containing 400 mL of liquid potato medium. These flasks were incubated on a rotary shaker at 26 °C with a shaking speed of 160 rpm. After a 24 h pre-culture period, 20 mg of the substrate dissolved in 2 mL of ethanol was added to each flask. Fermentation then proceeded for 7 days. In total, 2.0 g of UNA was used for *A. ochraceus* and 1.2 g for *A. oryzae*. After the 7-day incubation, the cultures from each flask were combined and filtered. The resulting filtrates underwent extraction with ethyl acetate (EtOAc) three times. The organic layers were then gathered, and after solvent evaporation, residues weighing 3.4 g and 2.3 g were obtained for *A. ochraceus* and *A. oryzae*, respectively.

### 4.4. Extraction and Isolation

*A. ochraceus* CGMCC 3.5324: The residual material, weighing 3.4 g, was subjected to silica gel column chromatography using a gradient elution of dichloromethane (CH_2_Cl_2_) to methanol (CH_3_OH) ranging from 100:1 to 1:1 (*v*/*v*). This separation yielded five primary fractions (Fr.1–Fr.5). These fractions were further purified using semi-preparative HPLC to isolate the pure compounds. Fr.1 produced compounds **4** (23.6 mg), **13** (24.5 mg), **18** (18.6 mg), and **19** (32.1 mg). Compound **2** (58.5 mg) was isolated from Fr.2. Fr.3 gave rise to compounds **14** (14.5 mg), **15** (28.8 mg), **3** (20.9 mg), and **5** (15.7 mg). Fr.4 produced compounds **16** (13.9 mg) and **17** (25.2 mg). Lastly, compounds **8** (23.6 mg), **9** (19.5 mg), and **1** (93.8 mg) were obtained from Fr.5.

*A. oryzae* CGMCC 3.407: The crude extract, with a mass of 2.3 g, underwent chromatographic separation on an ODS-C18 open column using a gradient elution of methanol (CH_3_OH) to water (H_2_O) with the following ratios: 20:80, 40:60, 60:40, 80:20, 90:10, and 100:0 (*v*/*v*). This resulted in five primary fractions (Fr.1–Fr.4). Each fraction was then further purified by semi-preparative HPLC to obtain the pure compounds. Specifically, Fr.1 yielded compounds **2** (20.5 mg), **6** (16.1 mg), **7** (13.6 mg), and **12** (15.4 mg). Fr.2 produced compounds **3** (22.1 mg), **4** (17.4 mg), **5** (23.8 mg), and **11** (19.2 mg). Fr.3 gave rise to compound **10** (18.5 mg), while Fr.4 produced compounds **8** (12.5 mg), **9** (24.4 mg), and **1** (78.2 mg).

### 4.5. Compound Characterization

*Characterization of 3,21-dioxo-urs-12-en-28-oic acid* (**4**), White powder; mp 232–225 °C; [α${]}_{D}^{25}$: +28.6° (*c* = 0.1, MeOH); IR (KBr): *ν*_max_ 3607, 2956, 1768, 1724, 1705, 1384, 1235, 1052 cm^−1^; ^1^H NMR (CDCl_3_, 400 MHz) and ^13^C NMR (CDCl_3_, 100 MHz) (for data, see Table 1); HR-ESI-MS: *m*/*z* 467.3174 [M − H]^−^ (calcd. for C_30_H_43_O_4_, 467.3161).

*Characterization of 3,21-dioxo-7β-hydroxy-urs-12-en-28-oic acid* (**5**), White powder; mp 251–254 °C; [α${]}_{D}^{25}$: +36.8° (*c* = 0.1, MeOH); IR (KBr): *ν*_max_ 3657, 2934, 1761, 1726, 1702, 1367, 1245, 1063 cm^−1^; ^1^H NMR (CDCl_3_, 400 MHz) and ^13^C NMR (CDCl_3_, 100 MHz) (for data, see Table 1); HR-ESI-MS: *m*/*z* 483.3126 [M − H]^−^ (calcd. for C_30_H_43_O_5_, 483.3112).

*Characterization of 3-oxo-11α-methoxy-21β-hydroxy-urs-12-en-28-oic acid* (**7**), White powder; mp 268–271 °C; [α${]}_{D}^{25}$: +18.9° (*c* = 0.1, MeOH); IR (KBr): *ν*_max_ 3667, 2965, 1763, 1711, 1376, 1239, 1042 cm^−1^; ^1^H NMR (CDCl_3_, 600 MHz) and ^13^C NMR (CDCl_3_, 150 MHz) (for data, see Table 1); HR-ESI-MS: *m*/*z* 499.3433 [M − H]^−^ (calcd. for C_31_H_47_O_5_, 499.3423).

*Characterization of 3-oxo-21β-hydroxy-urs-11-en-13β,28β-lactone* (**8**), White powder; mp 245–247 °C; [α${]}_{D}^{25}$: +30.2° (*c* = 0.1, MeOH); IR (KBr): *ν*_max_ 3673, 2954, 1742, 1703, 1388, 1264, 1039 cm^−1^; ^1^H NMR (CDCl_3_, 400 MHz) and ^13^C NMR (CDCl_3_, 100 MHz) (for data, see Table 2); HR-ESI-MS: *m*/*z* 451.3216 [M − H_2_O+H]^+^ (calcd. for C_30_H_43_O_3_, 451.3212).

*Characterization of 3-oxo-21β-hydroxy-urs-11-en-13β,28β-lactone* (**9**), White powder; mp 261–265 °C; [α${]}_{D}^{25}$: +58.5° (*c* = 0.1, MeOH); IR (KBr): *ν*_max_ 3685, 2972, 1751, 1712, 1371, 1255, 1046 cm^−1^; ^1^H NMR (CDCl_3_, 400 MHz) and ^13^C NMR (CDCl_3_, 100 MHz) (for data, see Table 2); HR-ESI-MS: *m*/*z* 529.3171 [M + COOH]^−^ (calcd. for C_31_H_45_O_7_, 529.3165).

*Characterization of 3-methoxy-urs-2,12-dien-28-oic acid* (**10**), White powder; mp 284–288 °C; [α${]}_{D}^{25}$: +31.7° (*c* = 0.1, MeOH); IR (KBr): *ν*_max_ 3631, 2955, 1719, 1383, 1264, 1052 cm^−1^; ^1^H NMR (CDCl_3_, 400 MHz) and ^13^C NMR (CDCl_3_, 100 MHz) (for data, see Table 2); HR-ESI-MS: *m*/*z* 467.3525 [M − H]^−^ (calcd. for C_31_H_47_O_3_, 467.3525).

*Characterization of 3-oxo-21β-hydroxy-11β,12β-epoxyl-urs-13β,28β-lactone* (**13**), White powder; mp 257–263 °C; [α${]}_{D}^{25}$: +47.1° (*c* = 0.1, MeOH); IR (KBr): *ν*_max_ 3612, 2943, 1765, 1718, 1377, 1226, 1048 cm^−1^; ^1^H NMR (CDCl_3_, 400 MHz) and ^13^C NMR (CDCl_3_, 100 MHz) (for data, see Table 3); HR-ESI-MS: *m*/*z* 529.3173 [M + COOH]^−^ (calcd. for C_31_H_45_O_7_, 529.3165).

*Characterization of 3-oxo-1β,21β-dihydroxyl-11β,12β-epoxyl-urs-13β,28β-lactone* (**14**), White powder; mp 278–280 °C; [α${]}_{D}^{25}$: +72.3° (*c* = 0.1, MeOH); IR (KBr): *ν*_max_ 3568, 3527, 2961, 1768, 1722, 1362, 1239, 1054 cm^−1^; ^1^H NMR (Pyridine-*d*_5_, 600 MHz) and ^13^C NMR (Pyridine-*d*_5_, 150 MHz) (for data, see Table 3); HR-ESI-MS: *m*/*z* 545.3156 [M + COOH]^−^ (calcd. for C_31_H_45_O_8_, 545.3114).

*Characterization of 3-oxo-7α,21β-dihydroxyl-11β,12β-epoxyl-urs-13β,28β-lactone* (**15**), White powder; mp 270–274 °C; [α${]}_{D}^{25}$: +24.5° (*c* = 0.1, MeOH); IR (KBr): *ν*_max_ 3685, 3618, 2956, 1767, 1717, 1322, 1247, 1053 cm^−1^; ^1^H NMR (CD_3_OD, 400 MHz) and ^13^C NMR (CD_3_OD, 100 MHz) (for data, see Table 3); HR-ESI-MS: *m*/*z* 545.3130 [M + COOH]^−^ (calcd. for C_31_H_45_O_8_, 545.3114).

*Characterization of 3-oxo-7β,21β-dihydroxyl-11β,12β-epoxyl-urs-13β,28β-lactone* (**16**), White powder; mp 268–273 °C; [α${]}_{D}^{25}$: +83.1° (*c* = 0.1, MeOH); IR (KBr): *ν*_max_ 3647, 3586, 2959, 1765, 1713, 1351, 1232, 1055 cm^−1^; ^1^H NMR (CD_3_OD, 500 MHz) and ^13^C NMR (CD_3_OD, 125 MHz) (for data, see Table 4); HR-ESI-MS: *m*/*z* 545.3123 [M + COOH]^−^ (calcd. for C_31_H_45_O_8_, 545.3114).

*Characterization of 3,21-dioxo-7β-hydroxyl-11β,12β-epoxyl-urs-13β,28β-lactone* (**17**), White powder; mp 247–249 °C; [α${]}_{D}^{25}$: +20.3° (*c* = 0.1, MeOH); IR (KBr): *ν*_max_ 3674, 2956, 1765, 1721, 1714, 1366, 1267, 1068 cm^−1^; ^1^H NMR (CDCl_3_, 400 MHz) and ^13^C NMR (CDCl_3_, 100 MHz) (for data, see Table 4); HR-ESI-MS: *m*/*z* 543.2968 [M + COOH]^−^ (calcd. for C_31_H_43_O_8_, 543.2958).

*Characterization of 3-oxo-21β-hydroxyl-11β,12β-epoxyl-urs-1-ene-13β,28β-lactone* (**18**), White powder; mp 242–245 °C; [α${]}_{D}^{25}$: +38.6° (*c* = 0.1, MeOH); IR (KBr): *ν*_max_ 3622, 3031, 2955, 1765, 1689, 1332, 1267, 1091 cm^−1^; ^1^H NMR (CD_3_OD, 500 MHz) and ^13^C NMR (CD_3_OD, 125 MHz) (for data, see Table 4); HR-ESI-MS: *m*/*z* 527.2993 [M + COOH]^−^ (calcd. for C_31_H_43_O_7_, 527.3009).

*Characterization of 3-oxo-21β-hydroxyl-7-methyl-11β,12β-epoxyl-7-ene-26-norurs-13β,28β-lactone* (**19**), White powder; mp 282–284 °C; [α${]}_{D}^{25}$: −35.7° (c = 0.1, MeOH). IR (KBr): ν_max_ 3598, 2971, 1767, 1716, 1380, 1233, 1048 cm^−1^; ^1^H NMR (CDCl_3_, 400 MHz) and ^13^C NMR (CDCl_3_, 100 MHz) (for data, see Table 4); HR-ESI-MS: *m*/*z* 527.3017 [M + COOH]^−^ (calcd. for C_31_H_43_O_7_, 527.3009).

### 4.6. Anti-Neuroinflammatory Activities

NO production was assessed indirectly using the Griess reaction by measuring nitrite concentration in a culture medium. BV-2 cells were cultured in Dulbecco’s Modified Eagle Medium, supplemented with 10% FBS, 100 units/mL of penicillin, and 100 μg/mL of streptomycin. These cells were seeded in 96-well plates at a density of 8 × 10^4^ cells/well and incubated for 24 h. Post incubation, they were exposed to 100 ng/mL of LPS alongside different compound concentrations for 48 h. A mixture of 100 μL culture supernatant and Griess reagent was left at room temperature for 10 min, with absorbance later read at 570 nm. L-NMMA served as the positive control. Cell viability was determined using the MTT assay, and experiments were conducted in triplicate. The IC_50_ for NO production inhibition was computed using GraphPad Prism 7.00 software.

### 4.7. X-ray Crystallographic Analyses

Colorless needle crystals of compound **10** were obtained using an acetone–H_2_O mixture (3:1). Similarly, compounds **13** and **19** were derived from a MeOH–H_2_O mixture (9:1), and compound **15** from an acetone–H_2_O mixture (8:2). A suitable crystal was chosen and analyzed on a Bruker APEX-II CCD diffractometer, with the crystal maintained at 173.0 K during data collection. The structure was deciphered using the ShelXT structure solution program within Olex2, employing Intrinsic Phasing. Refinement was performed with the ShelXL package using least squares minimization.

Deposition numbers for compounds **10**, **13**, **15**, and **19** in the Cambridge Crystallographic Data Centre (CCDC) are 2266165 and 2256926-2256928, respectively.

## Data Availability

The NMR data for compounds **4**, **5**, **7**–**10**, and **13**–**19** have been deposited to the Harvard Dataverse at https://doi.org/10.7910/DVN/TYF1KG.

## Supplementary Materials

The following supporting information can be downloaded at: https://www.mdpi.com/article/10.3390/molecules28247943/s1.
