# Supplementary material for: Biotransformation of Ursonic Acid by Aspergillus ochraceus and Aspergillus oryzae to Discover Anti-Neuroinflammatory Derivatives
Source: Molecules. 2023 Dec 5;28(24):7943. doi: 10.3390/molecules28247943 (PMC10745867; doi:10.3390/molecules28247943)
Supplement: Supplementary file 1 [file molecules-28-07943-s001.zip › molecules-2710556-supplementary.pdf]

# Supporting Information

# Biotransformation of Ursonic Acid by *Aspergillus ochraceus* and *Aspergillus oryzae* to Discover Anti-Neuroinflammatory Derivatives

**Yan-Ni Wu [1], Dan Su [1], Jia Yang [1], Ying Yi [1], An-Dong Wang [1], Min Yang [1], Jian-Lin Li [1], Bo-Yi Fan [1], Guang-Tong Chen [1,*], Wen-Li Wang [1] and Bai Ling [2,*]**

[1] School of Pharmacy, Nantong University, 19 Qixiu Road, Nantong 226001, China; wangwenli16@126.com (W.-L.W.)

[2] Department of Pharmacy, The Fourth Affiliated Hospital of Nantong University, The First People's Hospital of Yancheng, 166 Yulongxi Road, Yancheng 224005, China

* Correspondence: guangtong_chen@163.com (G.-T.C.); 18005108889@163.com (B.L.)

# Table of contents


**Table S1** Crystal data and structure refinement for compound **10** ········ 5
**Table S2** Crystal data and structure refinement for compound **13** ········ 6
**Table S3** Crystal data and structure refinement for compound **15** ········ 7
**Table S4** Crystal data and structure refinement for compound **19** ········ 8
**Figure S1** $^{1}$H-NMR spectrum of compound **4** ········ 9
**Figure S2** $^{13}$C-NMR spectrum of compound **4** ········ 9
**Figure S3** DEPT135 spectrum of compound **4** ········ 10
**Figure S4** HSQC spectrum of compound **4** ········ 10
**Figure S5** $^{1}$H-$^{1}$H COSY spectrum of compound **4** ········ 11
**Figure S6** HMBC spectrum of compound **4** ········ 11
**Figure S7** NOESY spectrum of compound **4** ········ 12
**Figure S8** HR-ESI-MS spectrum of compound **4** ········ 12
**Figure S9** $^{1}$H-NMR spectrum of compound **5** ········ 13
**Figure S10** $^{13}$C-NMR spectrum of compound **5** ········ 13
**Figure S11** DEPT135 spectrum of compound **5** ········ 14
**Figure S12** HSQC spectrum of compound **5** ········ 14
**Figure S13** $^{1}$H-$^{1}$H COSY spectrum of compound **5** ········ 15
**Figure S14** HMBC spectrum of compound **5** ········ 15
**Figure S15** NOESY spectrum of compound **5** ········ 16
**Figure S16** HR-ESI-MS spectrum of compound **5** ········ 16
**Figure S17** $^{1}$H-NMR spectrum of compound **7** ········ 17
**Figure S18** $^{13}$C-NMR spectrum of compound **7** ········ 17
**Figure S19** DEPT135 spectrum of compound **7** ········ 18
**Figure S20** HSQC spectrum of compound **7** ········ 18
**Figure S21** $^{1}$H-$^{1}$H COSY spectrum of compound **7** ········ 19
**Figure S22** HMBC spectrum of compound **7** ········ 19
**Figure S23** NOESY spectrum of compound **7** ········ 20
**Figure S24** HR-ESI-MS spectrum of compound **7** ········ 20
**Figure S25** $^{1}$H-NMR spectrum of compound **8** ········ 21
**Figure S26** $^{13}$C-NMR spectrum of compound **8** ········ 21
**Figure S27** DEPT135 spectrum of compound **8** ········ 22
**Figure S28** HSQC spectrum of compound **8** ········ 22
**Figure S29** $^{1}$H-$^{1}$H COSY spectrum of compound **8** ········ 23
**Figure S30** HMBC spectrum of compound **8** ········ 23
**Figure S31** NOESY spectrum of compound **8** ········ 24
**Figure S32** HR-ESI-MS spectrum of compound **8** ········ 24
**Figure S33** $^{1}$H-NMR spectrum of compound **9** ········ 25
**Figure S34** $^{13}$C-NMR spectrum of compound **9** ········ 25
**Figure S35** DEPT135 spectrum of compound **9** ········ 26
**Figure S36** HSQC spectrum of compound **9** ········ 26
**Figure S37** $^{1}$H-$^{1}$H COSY spectrum of compound **9** ········ 27
**Figure S38** HMBC spectrum of compound **9** ········ 27
**Figure S39** NOESY spectrum of compound **9** ········ 28
**Figure S40** HR-ESI-MS spectrum of compound **9** ········ 28
**Figure S41** $^{1}$H-NMR spectrum of compound **10** ········ 29
**Figure S42** $^{13}$C-NMR spectrum of compound **10** ········ 29
**Figure S43** DEPT135 spectrum of compound **10** ········ 30
**Figure S44** HSQC spectrum of compound **10** ········ 30
**Figure S45** $^{1}$H-$^{1}$H COSY spectrum of compound **10** ········ 31
**Figure S46** HMBC spectrum of compound **10** ········ 31
**Figure S47** NOESY spectrum of compound **10** ········ 32
**Figure S48** HR-ESI-MS spectrum of compound **10** ········ 32

**Figure S49** $^{1}$H-NMR spectrum of compound **13** ··· 33
**Figure S50** $^{13}$C-NMR spectrum of compound **13** ··· 33
**Figure S51** DEPT135 spectrum of compound **13** ··· 34
**Figure S52** HSQC spectrum of compound **13** ··· 34
**Figure S53** $^{1}$H-$^{1}$H COSY spectrum of compound **13** ··· 35
**Figure S54** HMBC spectrum of compound **13** ··· 35
**Figure S55** NOESY spectrum of compound **13** ··· 36
**Figure S56** HR-ESI-MS spectrum of compound **13** ··· 36
**Figure S57** $^{1}$H-NMR spectrum of compound **14** ··· 37
**Figure S58** $^{13}$C-NMR spectrum of compound **14** ··· 37
**Figure S59** DEPT135 spectrum of compound **14** ··· 38
**Figure S60** HSQC spectrum of compound **14** ··· 38
**Figure S61** $^{1}$H-$^{1}$H COSY spectrum of compound **14** ··· 39
**Figure S62** HMBC spectrum of compound **14** ··· 39
**Figure S63** NOESY spectrum of compound **14** ··· 40
**Figure S64** HR-ESI-MS spectrum of compound **14** ··· 40
**Figure S65** $^{1}$H-NMR spectrum of compound **15** ··· 41
**Figure S66** $^{13}$C-NMR spectrum of compound **15** ··· 41
**Figure S67** DEPT135 spectrum of compound **15** ··· 42
**Figure S68** HSQC spectrum of compound **15** ··· 42
**Figure S69** $^{1}$H-$^{1}$H COSY spectrum of compound **15** ··· 43
**Figure S70** HMBC spectrum of compound **15** ··· 43
**Figure S71** NOESY spectrum of compound **15** ··· 44
**Figure S72** HR-ESI-MS spectrum of compound **15** ··· 44
**Figure S73** $^{1}$H-NMR spectrum of compound **16** ··· 45
**Figure S74** $^{13}$C-NMR spectrum of compound **16** ··· 45
**Figure S75** DEPT135 spectrum of compound **16** ··· 46
**Figure S76** HSQC spectrum of compound **16** ··· 46
**Figure S77** $^{1}$H-$^{1}$H COSY spectrum of compound **16** ··· 47
**Figure S78** HMBC spectrum of compound **16** ··· 47
**Figure S79** NOESY spectrum of compound **16** ··· 48
**Figure S80** HR-ESI-MS spectrum of compound **16** ··· 48
**Figure S81** $^{1}$H-NMR spectrum of compound **17** ··· 49
**Figure S82** $^{13}$C-NMR spectrum of compound **17** ··· 49
**Figure S83** DEPT135 spectrum of compound **17** ··· 50
**Figure S84** HSQC spectrum of compound **17** ··· 50
**Figure S85** $^{1}$H-$^{1}$H COSY spectrum of compound **17** ··· 51
**Figure S86** HMBC spectrum of compound **17** ··· 51
**Figure S87** NOESY spectrum of compound **17** ··· 52
**Figure S88** HR-ESI-MS spectrum of compound **17** ··· 52
**Figure S89** $^{1}$H-NMR spectrum of compound **18** ··· 53
**Figure S90** $^{13}$C-NMR spectrum of compound **18** ··· 53
**Figure S91** DEPT135 spectrum of compound **18** ··· 54
**Figure S92** HSQC spectrum of compound **18** ··· 54
**Figure S93** $^{1}$H-$^{1}$H COSY spectrum of compound **18** ··· 55
**Figure S94** HMBC spectrum of compound **18** ··· 55
**Figure S95** NOESY spectrum of compound **18** ··· 56
**Figure S96** HR-ESI-MS spectrum of compound **18** ··· 56
**Figure S97** $^{1}$H-NMR spectrum of compound **19** ··· 57
**Figure S98** $^{13}$C-NMR spectrum of compound **19** ··· 57
**Figure S99** DEPT135 spectrum of compound **19** ··· 58
**Figure S100** HSQC spectrum of compound **19** ··· 58
**Figure S101** $^{1}$H-$^{1}$H COSY spectrum of compound **19** ··· 59

**Figure S102** HMBC spectrum of compound **19** ··· 59
**Figure S103** NOESY spectrum of compound **19** ··· 60
**Figure S104** HR-ESI-MS spectrum of compound **19** ··· 60

**Table S1.** Crystal data and structure refinement for compound **10**

| | |
|---|---|
| Identification code | cu_20221190_0m_sq_sq |
| Empirical formula | $C_{31}H_{48}O_3$ |
| Formula weight | 468.69 |
| Temperature/K | 173.0 |
| Crystal system | monoclinic |
| Space group | $P2_1$ |
| a/Å | 14.3799(8) |
| b/Å | 7.9576(4) |
| c/Å | 24.7623(15) |
| α/° | 90 |
| β/° | 101.068(4) |
| γ/° | 90 |
| Volume/Å$^3$ | 2780.8(3) |
| Z | 4 |
| $\rho_{calc}$g/cm$^3$ | 1.119 |
| μ/mm$^{-1}$ | 0.537 |
| F(000) | 1032.0 |
| Crystal size/mm$^3$ | 0.07 × 0.03 × 0.01 |
| Radiation | CuKα (λ = 1.54178) |
| 2Θ range for data collection/° | 6.262 to 124.748 |
| Index ranges | $-16 \leq h \leq 16$, $-5 \leq k \leq 9$, $-28 \leq l \leq 26$ |
| Reflections collected | 12677 |
| Independent reflections | 6322 [$R_{int}$ = 0.0856, $R_{sigma}$ = 0.1046] |
| Data/restraints/parameters | 6322/1/631 |
| Goodness-of-fit on $F^2$ | 1.142 |
| Final R indexes [I>=2σ (I)] | $R_1$ = 0.1085, $wR_2$ = 0.2960 |
| Final R indexes [all data] | $R_1$ = 0.1502, $wR_2$ = 0.3421 |
| Largest diff. peak/hole / e Å$^{-3}$ | 0.51/-0.39 |
| Flack parameter | -0.4(5) |

**Table S2.** Crystal data and structure refinement for compound **13**

| | |
|---|---|
| Identification code | XJTS13_0m |
| Empirical formula | $C_{30}H_{44}O_5$ |
| Formula weight | 484.65 |
| Temperature/K | 170.0 |
| Crystal system | orthorhombic |
| Space group | $P2_12_12_1$ |
| a/Å | 6.70930(10) |
| b/Å | 14.3484(2) |
| c/Å | 26.2474(4) |
| α/° | 90 |
| β/° | 90 |
| γ/° | 90 |
| Volume/Å$^3$ | 2526.78(6) |
| Z | 4 |
| $\rho_{calc}$g/cm$^3$ | 1.274 |
| μ/mm$^{-1}$ | 0.672 |
| F(000) | 1056.0 |
| Crystal size/mm$^3$ | 0.12 × 0.08 × 0.05 |
| Radiation | CuKα (λ = 1.54178) |
| 2Θ range for data collection/° | 6.736 to 149.31 |
| Index ranges | $-8 \le h \le 7$, $-17 \le k \le 17$, $-32 \le l \le 32$ |
| Reflections collected | 33867 |
| Independent reflections | 5138 [$R_{int}$ = 0.0539, $R_{sigma}$ = 0.0344] |
| Data/restraints/parameters | 5138/0/324 |
| Goodness-of-fit on $F^2$ | 1.058 |
| Final R indexes [I>=2σ (I)] | $R_1$ = 0.0367, $wR_2$ = 0.0887 |
| Final R indexes [all data] | $R_1$ = 0.0406, $wR_2$ = 0.0919 |
| Largest diff. peak/hole / e Å$^{-3}$ | 0.28/-0.19 |
| Flack parameter | 0.08(8) |

**Table S3.** Crystal data and structure refinement for compound **15**

| | |
|---|---|
| Identification code | x45_0m |
| Empirical formula | $C_{30}H_{46}O_7$ |
| Formula weight | 518.67 |
| Temperature/K | 170.0 |
| Crystal system | monoclinic |
| Space group | $P2_1$ |
| a/Å | 12.3958(7) |
| b/Å | 7.8656(5) |
| c/Å | 28.4418(17) |
| α/° | 90 |
| β/° | 96.882(3) |
| γ/° | 90 |
| Volume/Å$^3$ | 2753.1(3) |
| Z | 4 |
| $\rho_{calc}$g/cm$^3$ | 1.251 |
| μ/mm$^{-1}$ | 0.705 |
| F(000) | 1128.0 |
| Crystal size/mm$^3$ | 0.08 × 0.05 × 0.04 |
| Radiation | CuKα (λ = 1.54178) |
| 2Θ range for data collection/° | 6.26 to 150.308 |
| Index ranges | $-14 \leq h \leq 15$, $-9 \leq k \leq 9$, $-35 \leq l \leq 35$ |
| Reflections collected | 35468 |
| Independent reflections | 10864 [$R_{int}$ = 0.0527, $R_{sigma}$ = 0.0523] |
| Data/restraints/parameters | 10864/10/698 |
| Goodness-of-fit on F$^2$ | 1.090 |
| Final R indexes [I>=2σ (I)] | $R_1$ = 0.0854, $wR_2$ = 0.2337 |
| Final R indexes [all data] | $R_1$ = 0.0877, $wR_2$ = 0.2352 |
| Largest diff. peak/hole / e Å$^{-3}$ | 0.49/-0.37 |
| Flack parameter | 0.04(6) |

**Table S4.** Crystal data and structure refinement for compound **19**

| | |
|---|---|
| Identification code | 12_0m |
| Empirical formula | $C_{30}H_{44}O_6$ |
| Formula weight | 500.65 |
| Temperature/K | 170.0 |
| Crystal system | orthorhombic |
| Space group | $P2_12_12_1$ |
| a/Å | 6.4846(2) |
| b/Å | 14.6936(6) |
| c/Å | 28.2892(12) |
| α/° | 90 |
| β/° | 90 |
| γ/° | 90 |
| Volume/Å$^3$ | 2695.46(18) |
| Z | 4 |
| $\rho_{calc}$g/cm$^3$ | 1.234 |
| μ/mm$^{-1}$ | 0.675 |
| F(000) | 1088.0 |
| Crystal size/mm$^3$ | 0.09 × 0.06 × 0.05 |
| Radiation | CuKα (λ = 1.54178) |
| 2Θ range for data collection/° | 6.248 to 149.172 |
| Index ranges | -7 ≤ h ≤ 8, -18 ≤ k ≤ 18, -33 ≤ l ≤ 34 |
| Reflections collected | 43437 |
| Independent reflections | 5462 [$R_{int}$ = 0.0744, $R_{sigma}$ = 0.0361] |
| Data/restraints/parameters | 5462/0/336 |
| Goodness-of-fit on F$^2$ | 1.030 |
| Final R indexes [I>=2σ (I)] | $R_1$ = 0.0465, $wR_2$ = 0.1185 |
| Final R indexes [all data] | $R_1$ = 0.0534, $wR_2$ = 0.1239 |
| Largest diff. peak/hole / e Å$^{-3}$ | 0.38/-0.17 |
| Flack parameter | -0.09(10) |

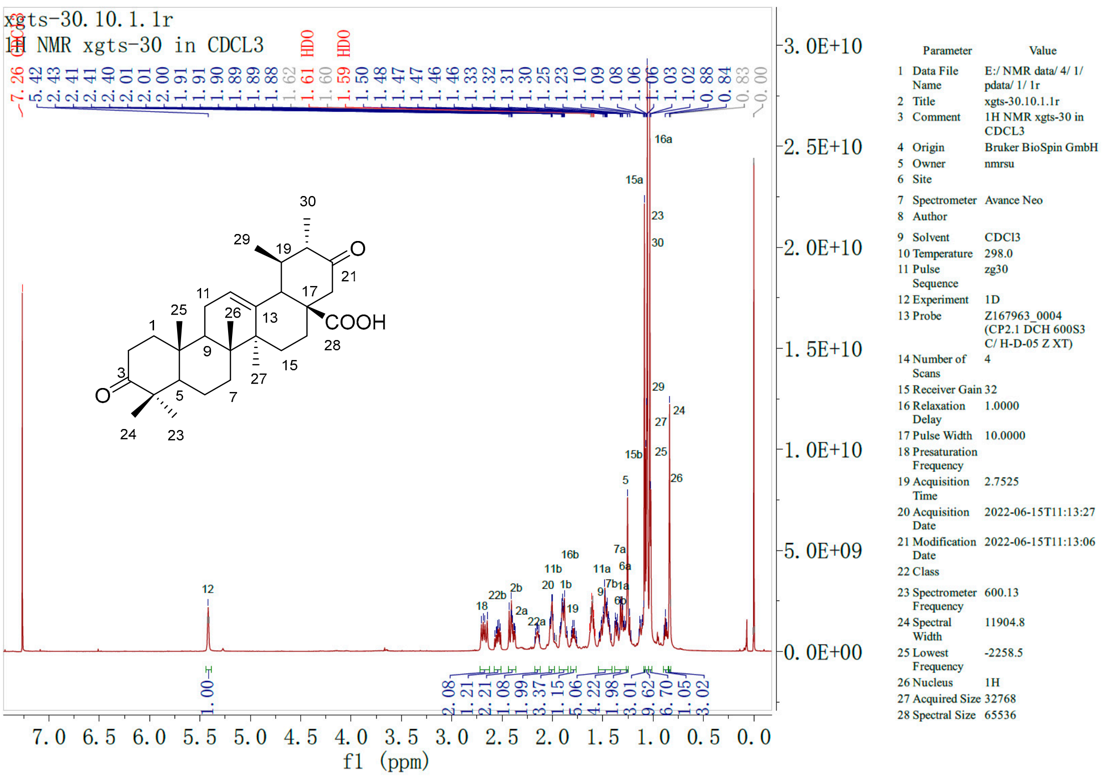

**Figure S1** $^{1}$H NMR spectrum (400 MHz) of compound **4** in $CDCl_3$

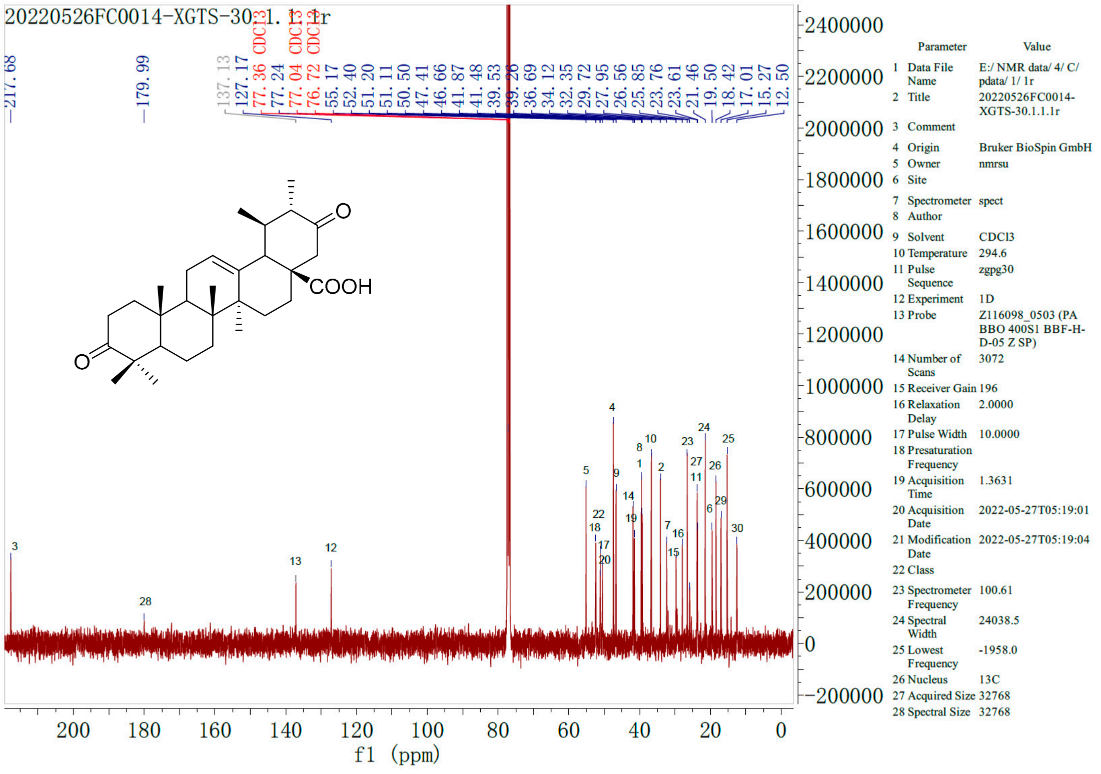

**Figure S2** $^{13}$C NMR spectrum (100 MHz) of compound **4** in $CDCl_3$

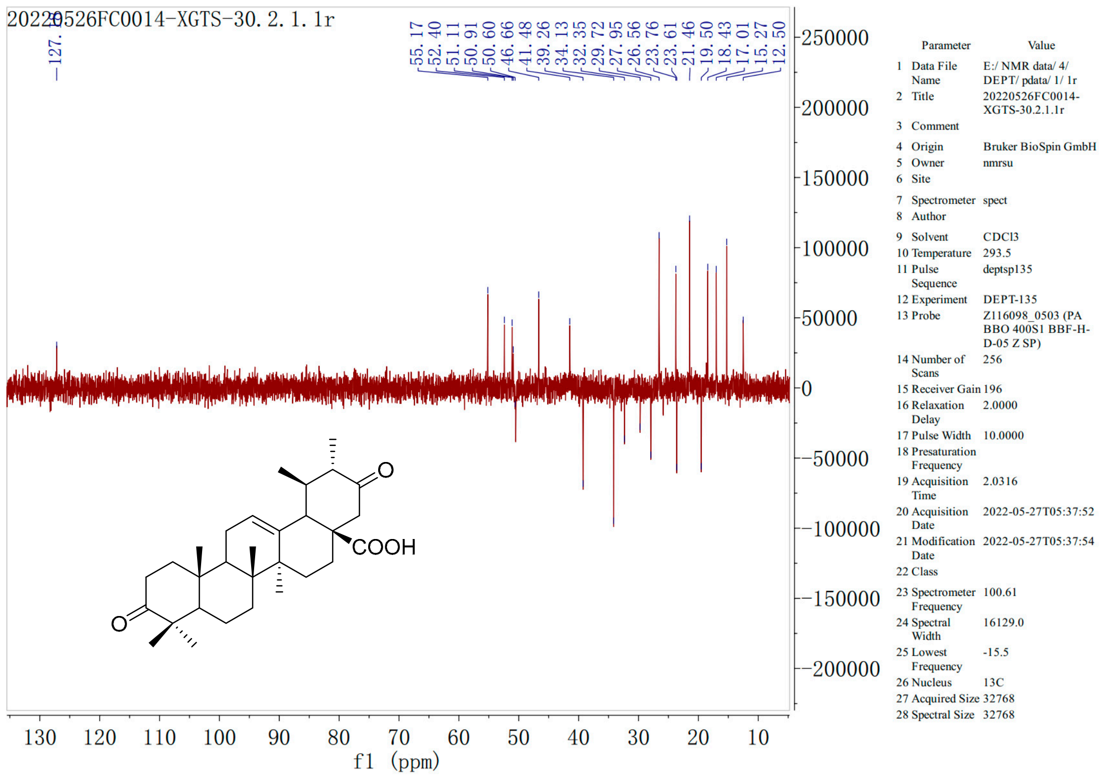


**Figure S3** DEPT-135 spectrum (100 MHz) of compound **4** in $CDCl_3$

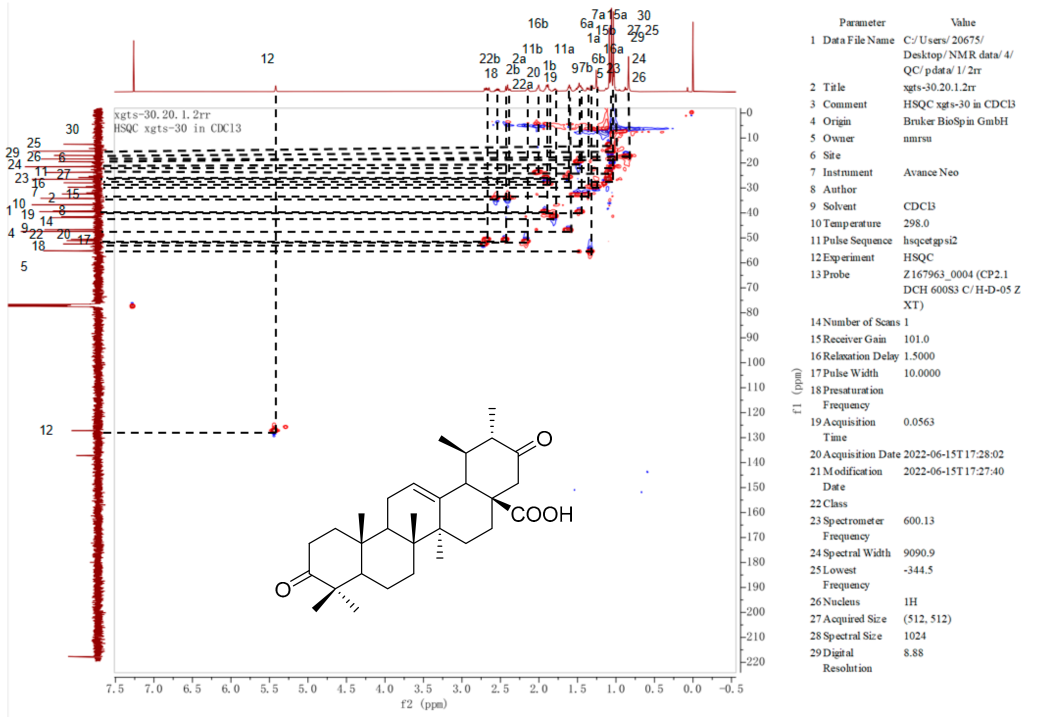


**Figure S4** HSQC spectrum (100 MHz) of compound **4** in $CDCl_3$

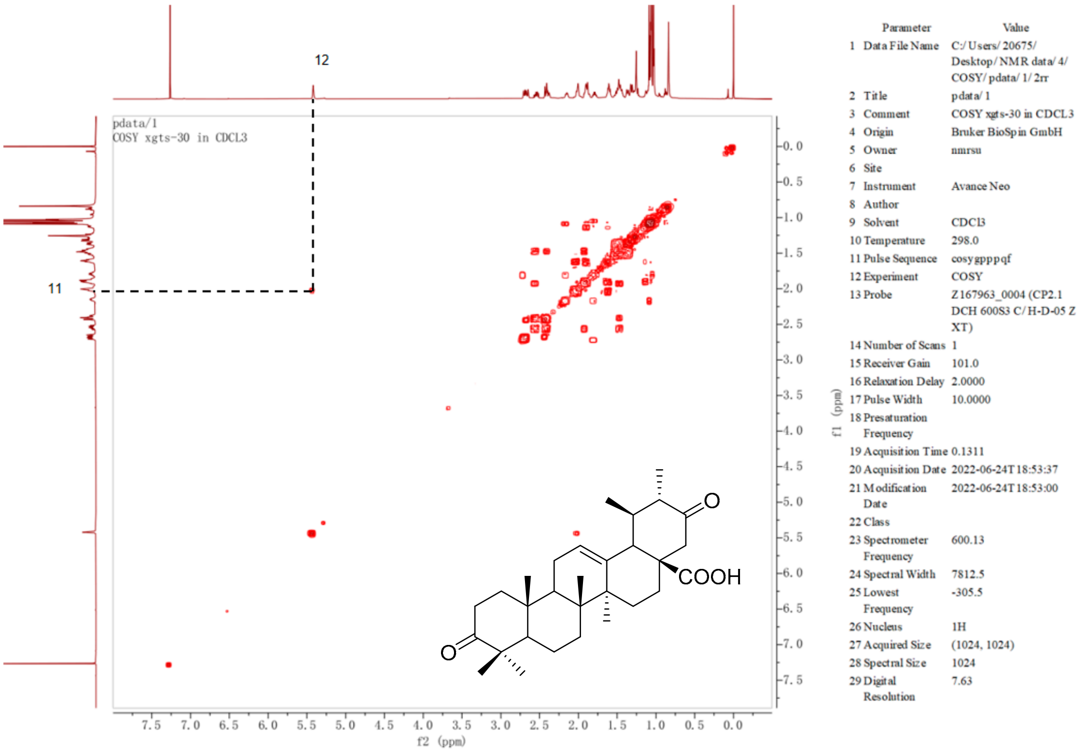


**Figure S5** $^{1}H$-$^{1}H$ COSY spectrum (400 MHz) of compound **4** in $CDCl_3$

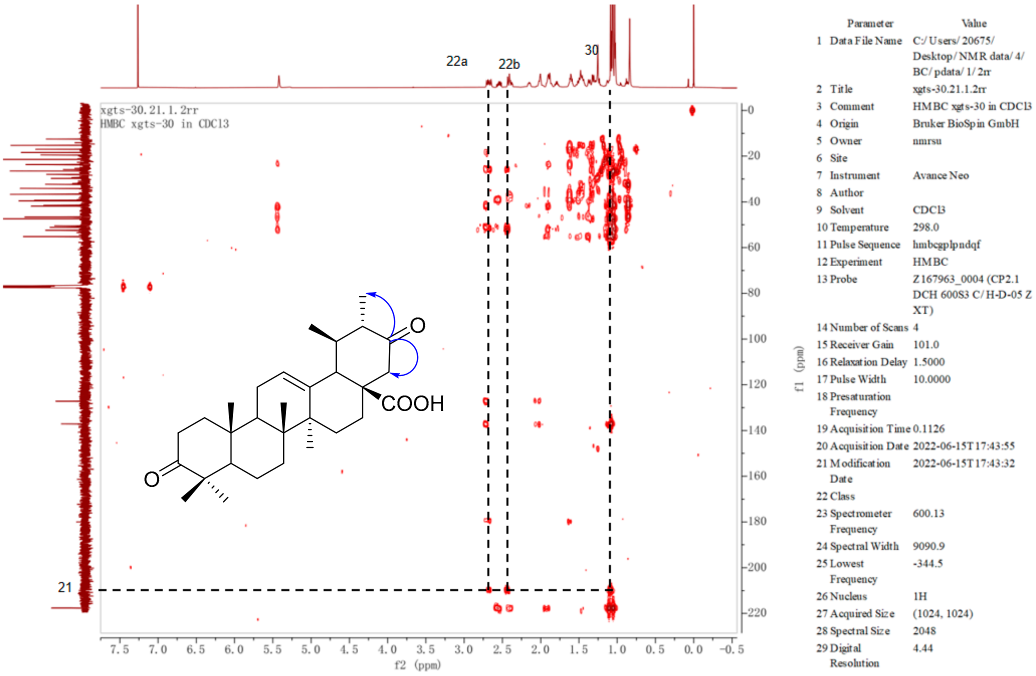


**Figure S6** HMBC spectrum (100 MHz) of compound **4** in $CDCl_3$

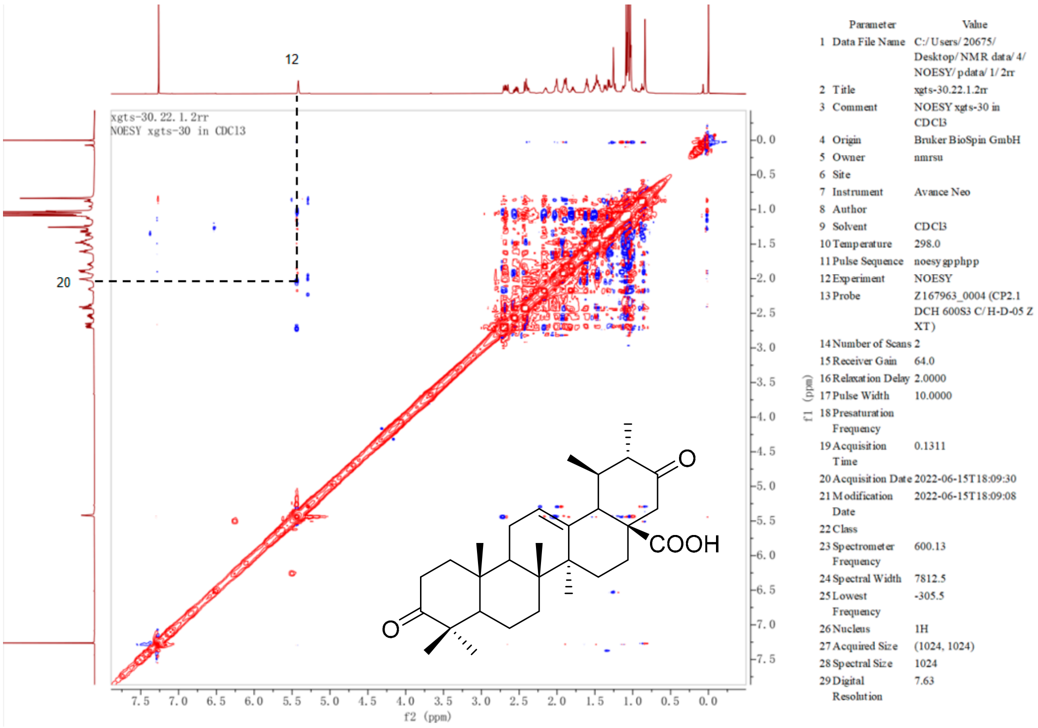


**Figure S7** NOESY spectrum (400 MHz) of compound **4** in $CDCl_3$

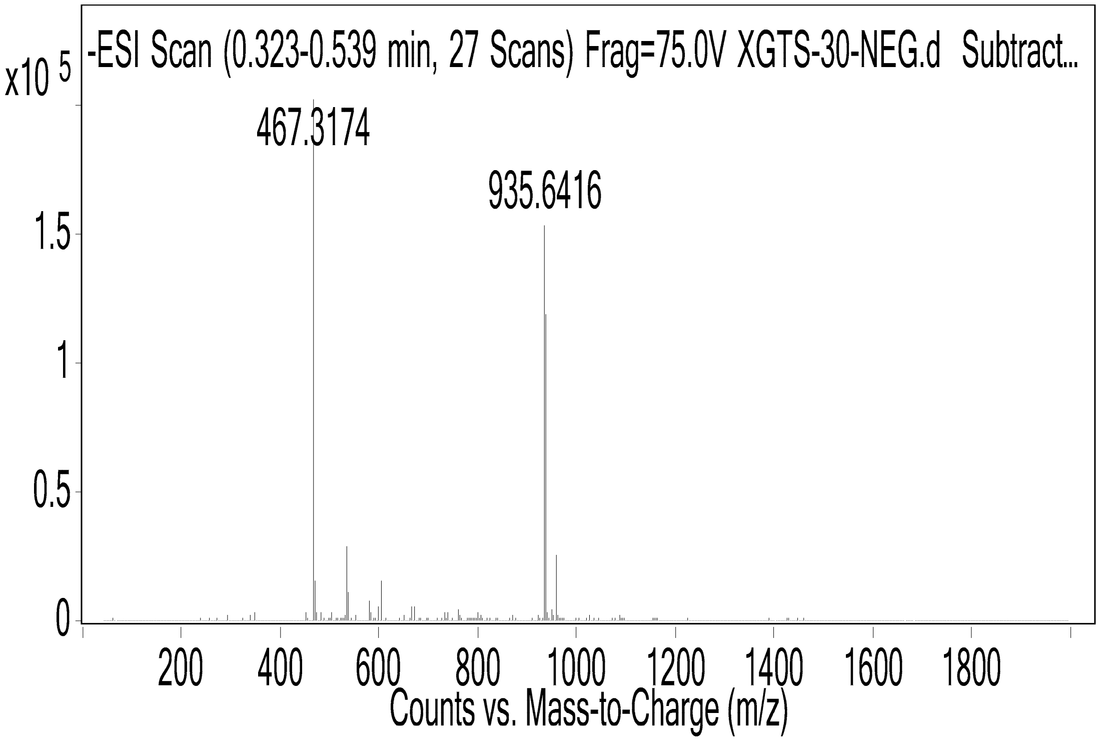


**Figure S8** HR-ESI-MS spectrum of compound **4**

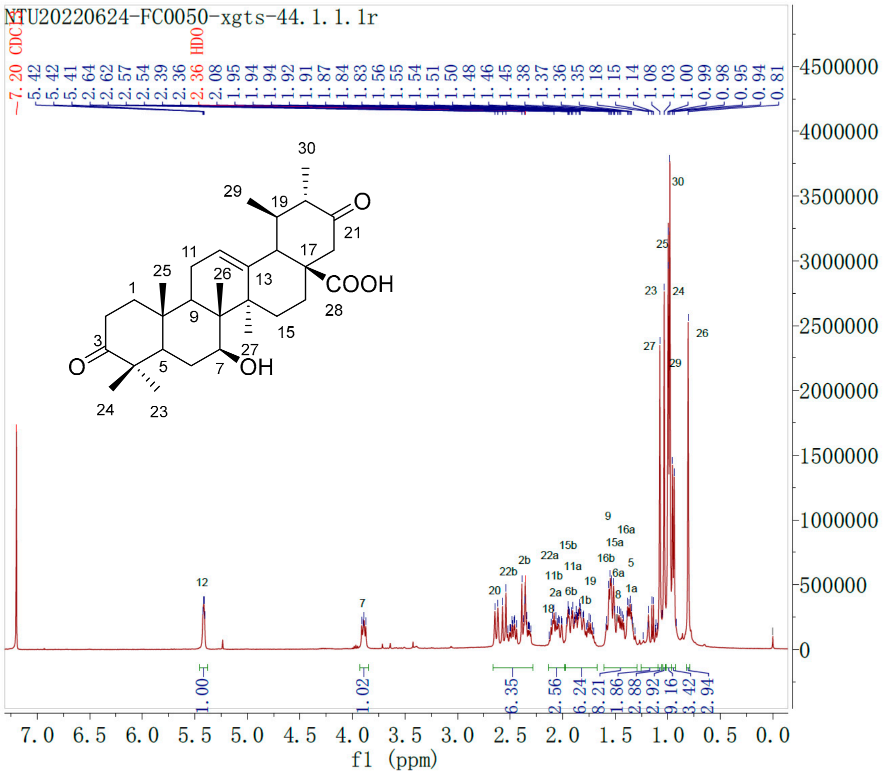


| Parameter | | Value |
|---|---|---|
| 1 | Data File Name | E:/ NMR data/ 5/ H/ pdata/ 1/ 1r |
| 2 | Title | NTU20220624-FC0050-xgts-44.1.1.1r |
| 3 | Comment | |
| 4 | Origin | Bruker BioSpin GmbH |
| 5 | Owner | nmrsu |
| 6 | Site | |
| 7 | Spectrometer | spect |
| 8 | Author | |
| 9 | Solvent | CDCl3 |
| 10 | Temperature | 0.0 |
| 11 | Pulse Sequence | zg30 |
| 12 | Experiment | 1D |
| 13 | Probe | Z116098_0503 (PA BBO 400S1 BBF-H-D-05 Z SP) |
| 14 | Number of Scans | 16 |
| 15 | Receiver Gain | 86 |
| 16 | Relaxation Delay | 1.0000 |
| 17 | Pulse Width | 9.7000 |
| 18 | Presaturation Frequency | |
| 19 | Acquisition Time | 4.0894 |
| 20 | Acquisition Date | 2022-06-24T10:59:11 |
| 21 | Modification Date | 2022-06-24T10:59:14 |
| 22 | Class | |
| 23 | Spectrometer Frequency | 400.13 |
| 24 | Spectral Width | 8012.8 |
| 25 | Lowest Frequency | -1570.4 |
| 26 | Nucleus | 1H |
| 27 | Acquired Size | 32768 |
| 28 | Spectral Size | 65536 |

**Figure S9** $^{1}H$ NMR spectrum (400 MHz) of compound **5** in $CDCl_3$

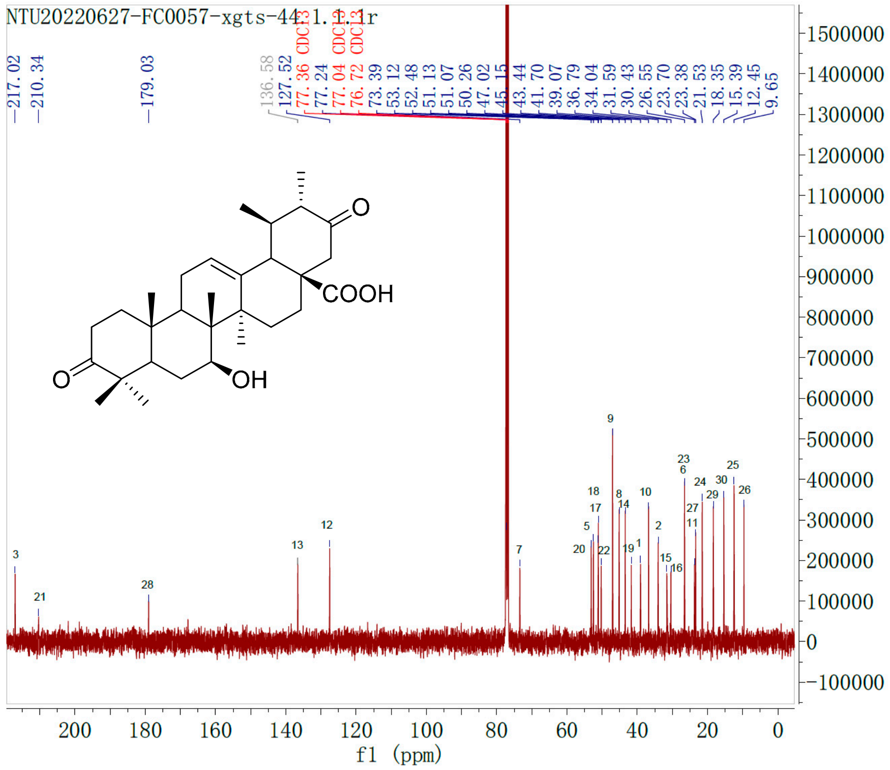


| Parameter | | Value |
|---|---|---|
| 1 | Data File Name | E:/ NMR data/ 5/ C/ pdata/ 1/ 1r |
| 2 | Title | NTU20220627-FC0057-xgts-44.1.1.1r |
| 3 | Comment | |
| 4 | Origin | Bruker BioSpin GmbH |
| 5 | Owner | nmrsu |
| 6 | Site | |
| 7 | Spectrometer | spect |
| 8 | Author | |
| 9 | Solvent | CDCl3 |
| 10 | Temperature | 0.0 |
| 11 | Pulse Sequence | zgpg30 |
| 12 | Experiment | 1D |
| 13 | Probe | Z116098_0503 (PA BBO 400S1 BBF-H-D-05 Z SP) |
| 14 | Number of Scans | 1024 |
| 15 | Receiver Gain | 196 |
| 16 | Relaxation Delay | 2.0000 |
| 17 | Pulse Width | 10.0000 |
| 18 | Presaturation Frequency | |
| 19 | Acquisition Time | 1.3631 |
| 20 | Acquisition Date | 2022-06-27T17:41:49 |
| 21 | Modification Date | 2022-06-27T17:41:52 |
| 22 | Class | |
| 23 | Spectrometer Frequency | 100.61 |
| 24 | Spectral Width | 24038.5 |
| 25 | Lowest Frequency | -1958.0 |
| 26 | Nucleus | 13C |
| 27 | Acquired Size | 32768 |
| 28 | Spectral Size | 32768 |

**Figure S10** $^{13}C$ NMR spectrum (100 MHz) of compound **5** in $CDCl_3$

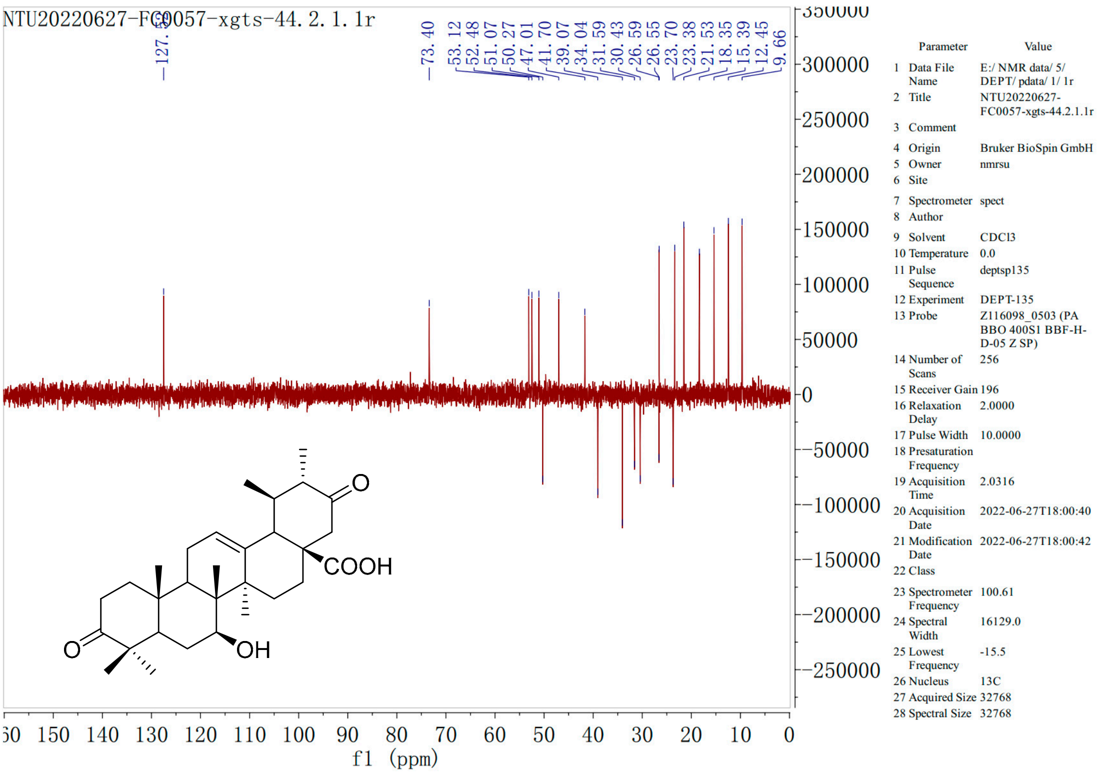


**Figure S11** DEPT-135 spectrum (100 MHz) of compound **5** in $CDCl_3$

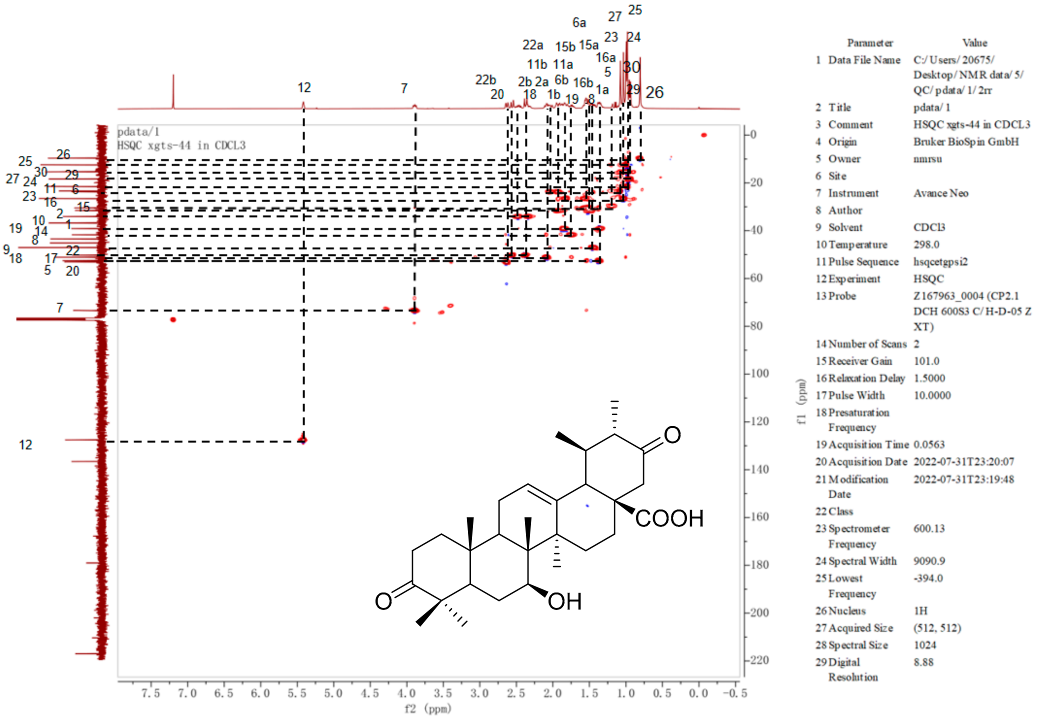


**Figure S12** HSQC spectrum (100 MHz) of compound **5** in $CDCl_3$

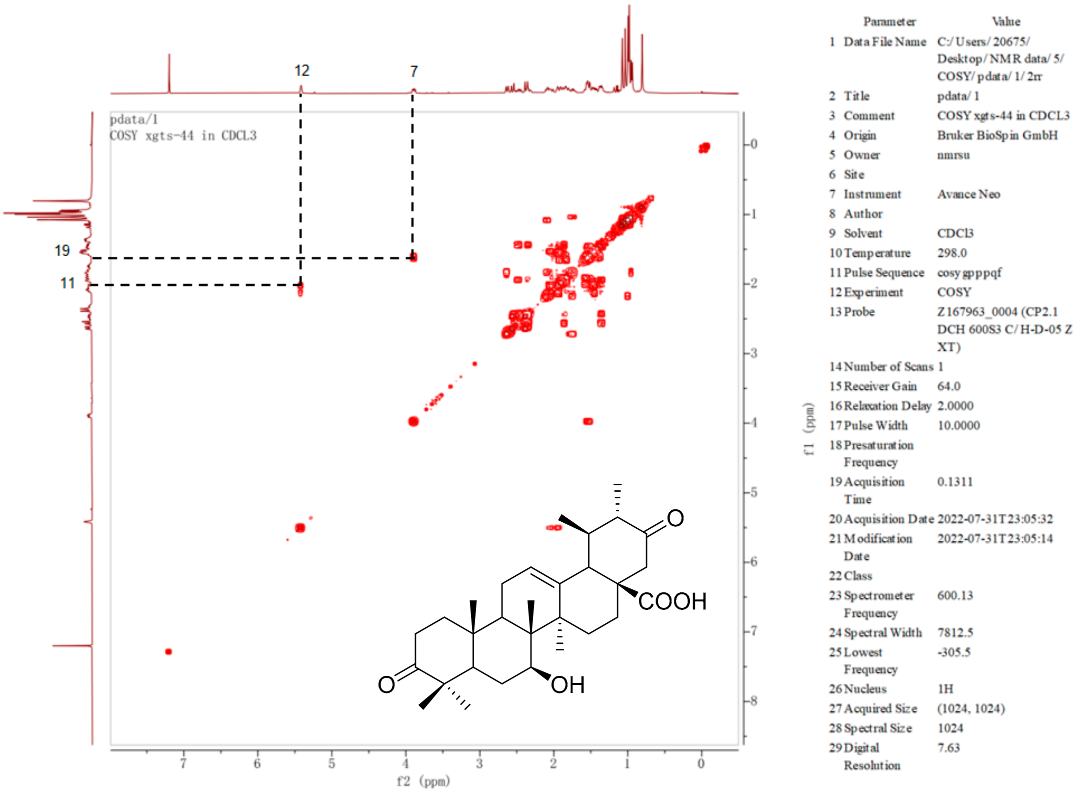


**Figure S13** $^1$H-$^1$H COSY spectrum (400 MHz) of compound **5** in $CDCl_3$

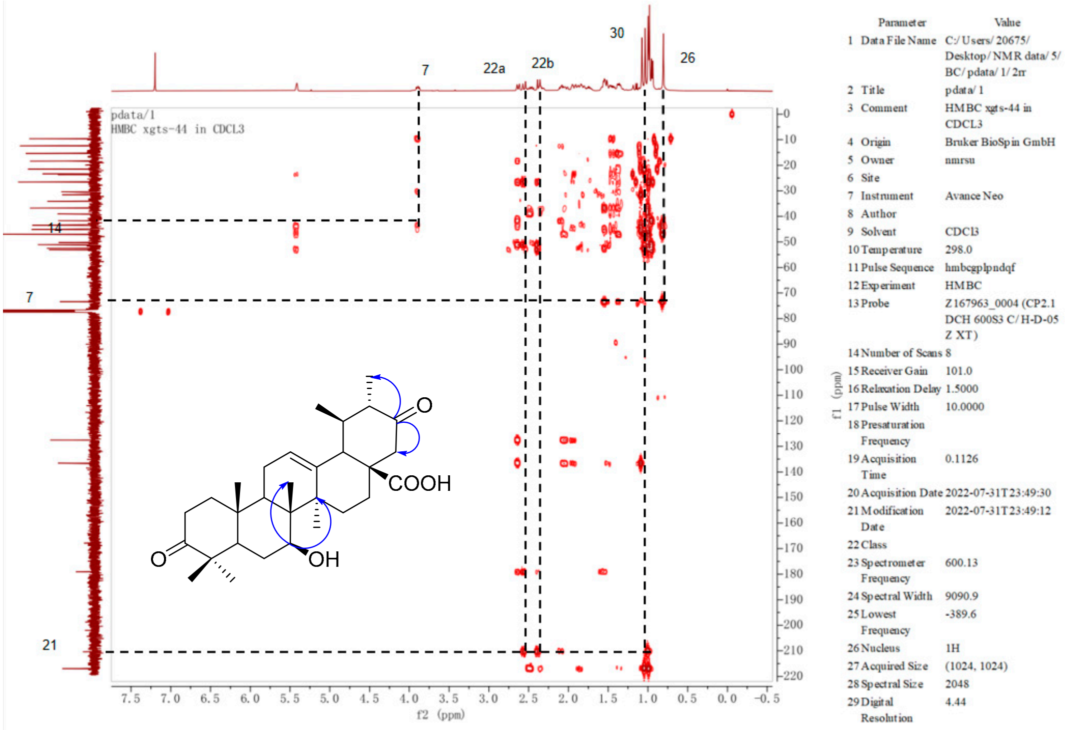


**Figure S14** HMBC spectrum (100 MHz) of compound **5** in $CDCl_3$

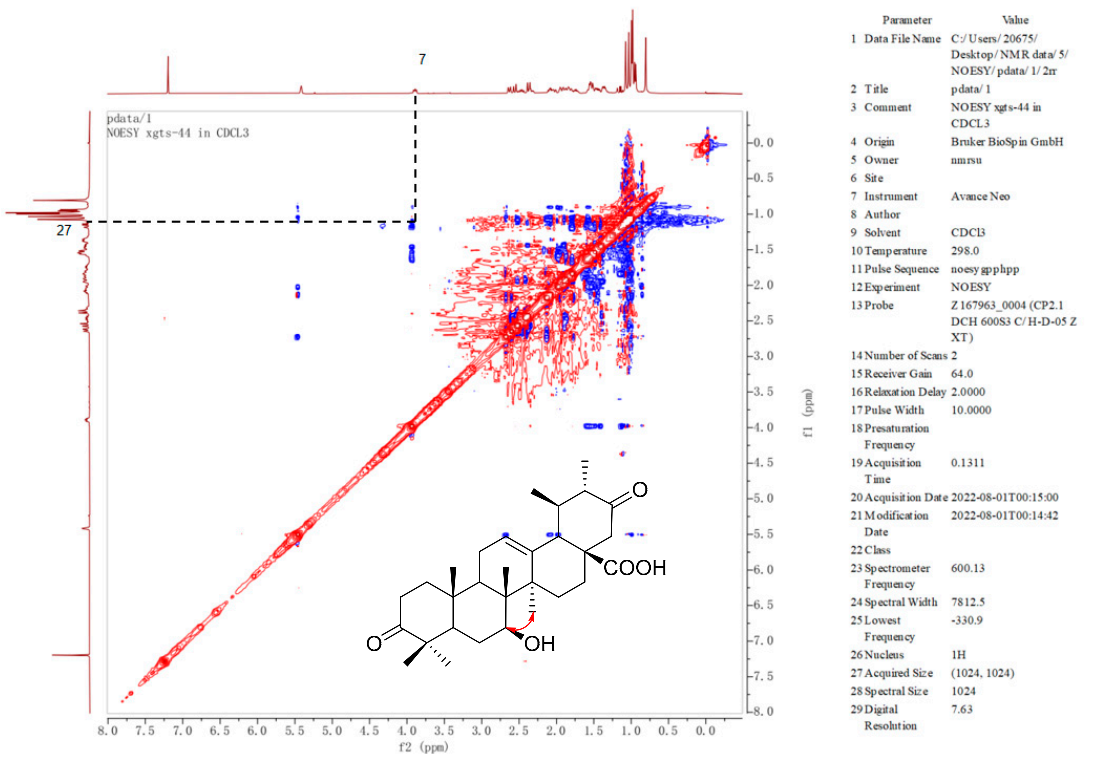


**Figure S15** NOESY spectrum (400 MHz) of compound **5** in $CDCl_3$

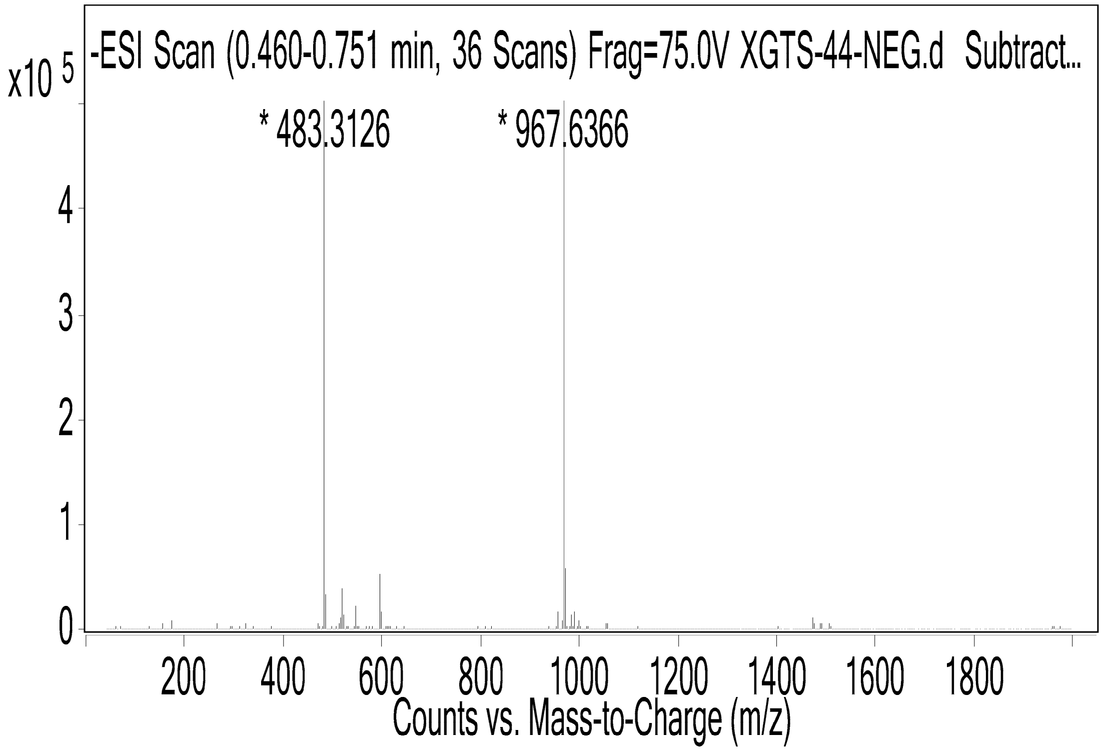


**Figure S16** HR-ESI-MS spectrum of compound **5**

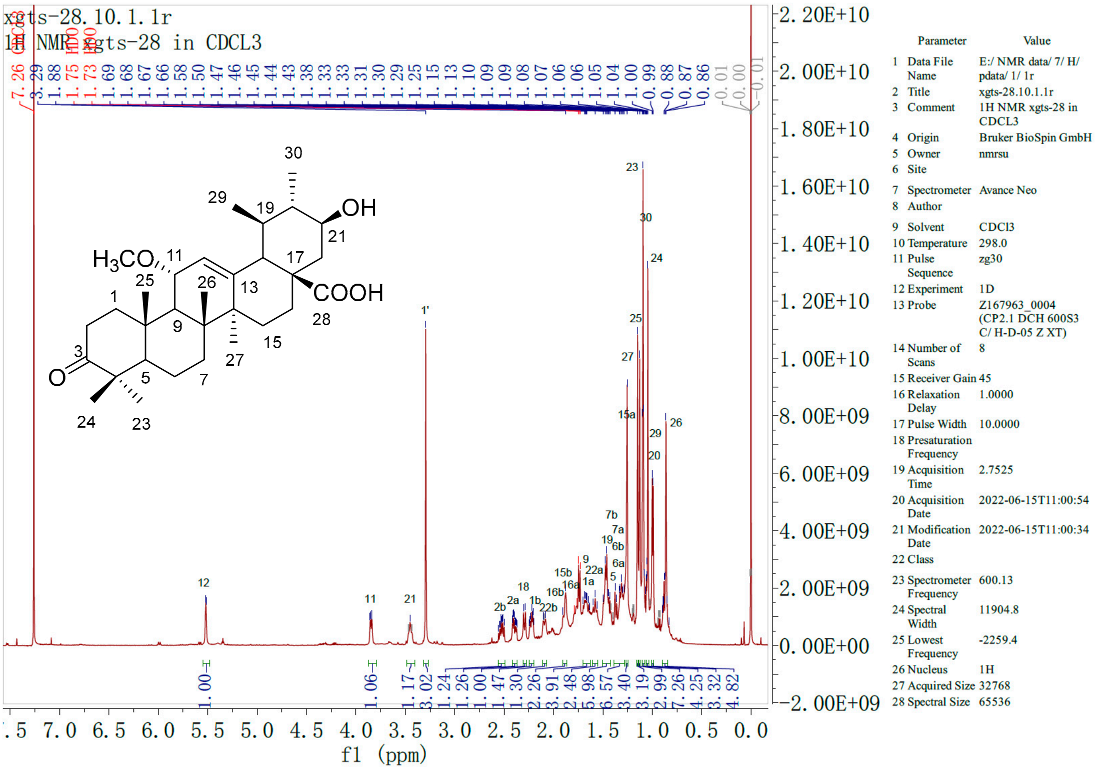


**Figure S17** $^{1}H$ NMR spectrum (600 MHz) of compound **7** in $CDCl_3$

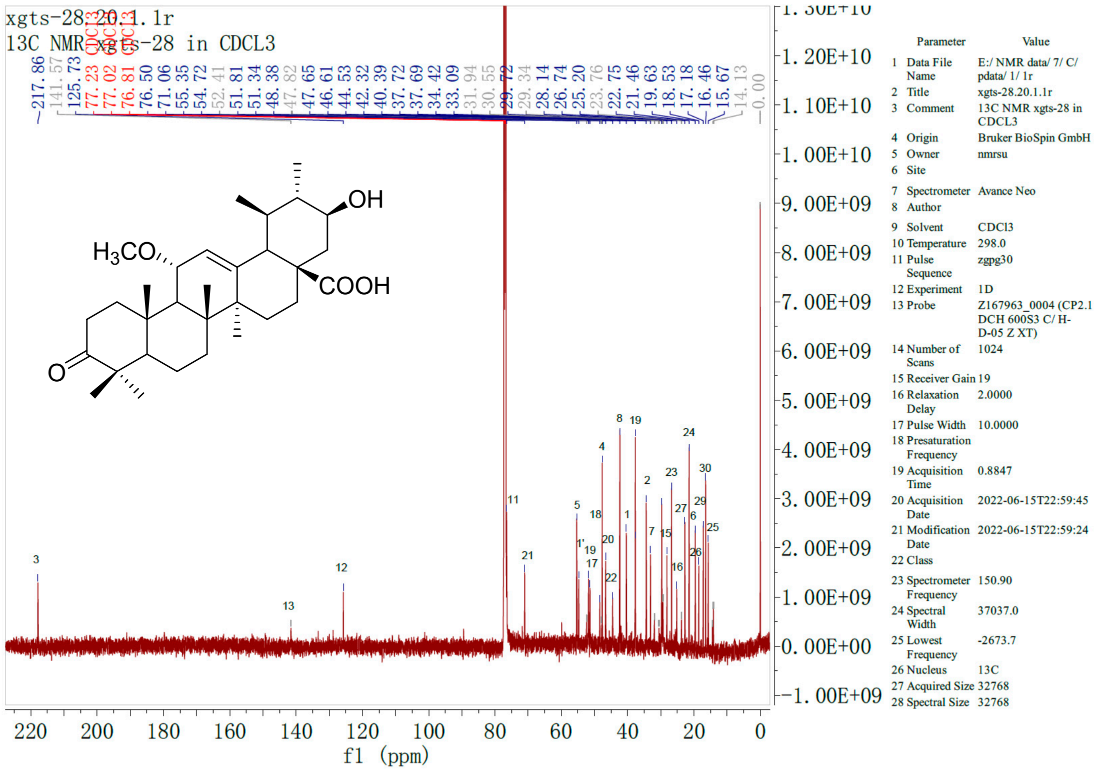


**Figure S18** $^{13}C$ NMR spectrum (150 MHz) of compound **7** in $CDCl_3$

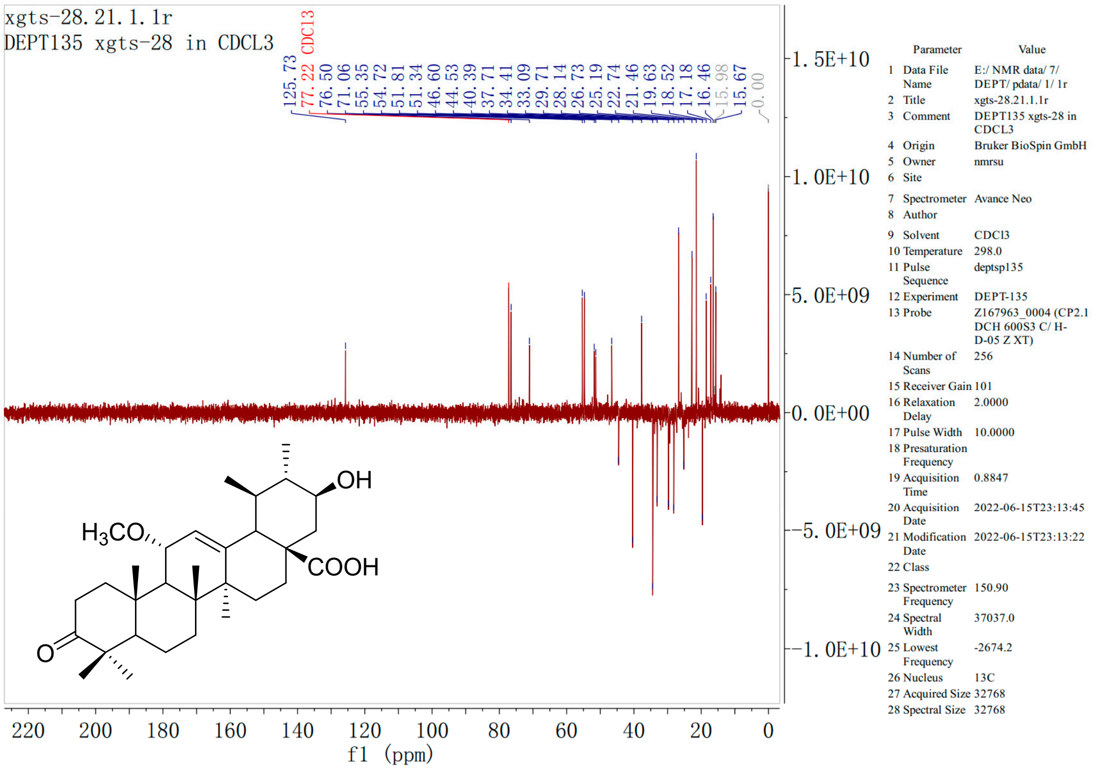


**Figure S19** DEPT-135 spectrum (150 MHz) of compound **7** in $CDCl_3$

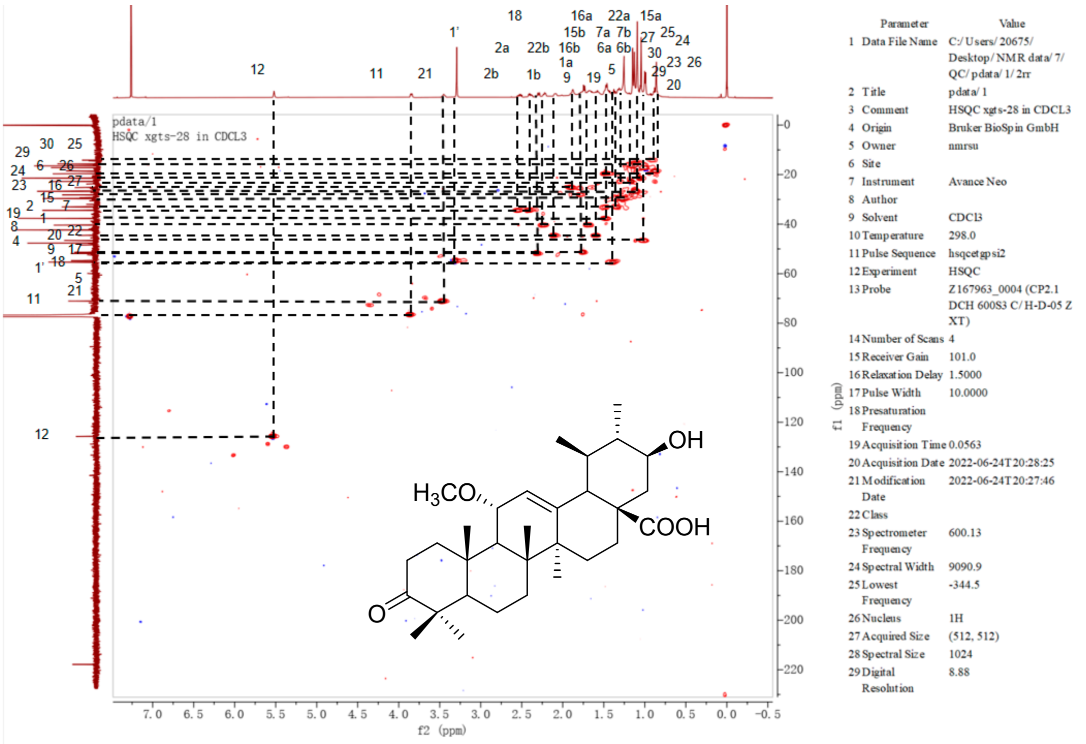


**Figure S20** HSQC spectrum (150 MHz) of compound **7** in $CDCl_3$

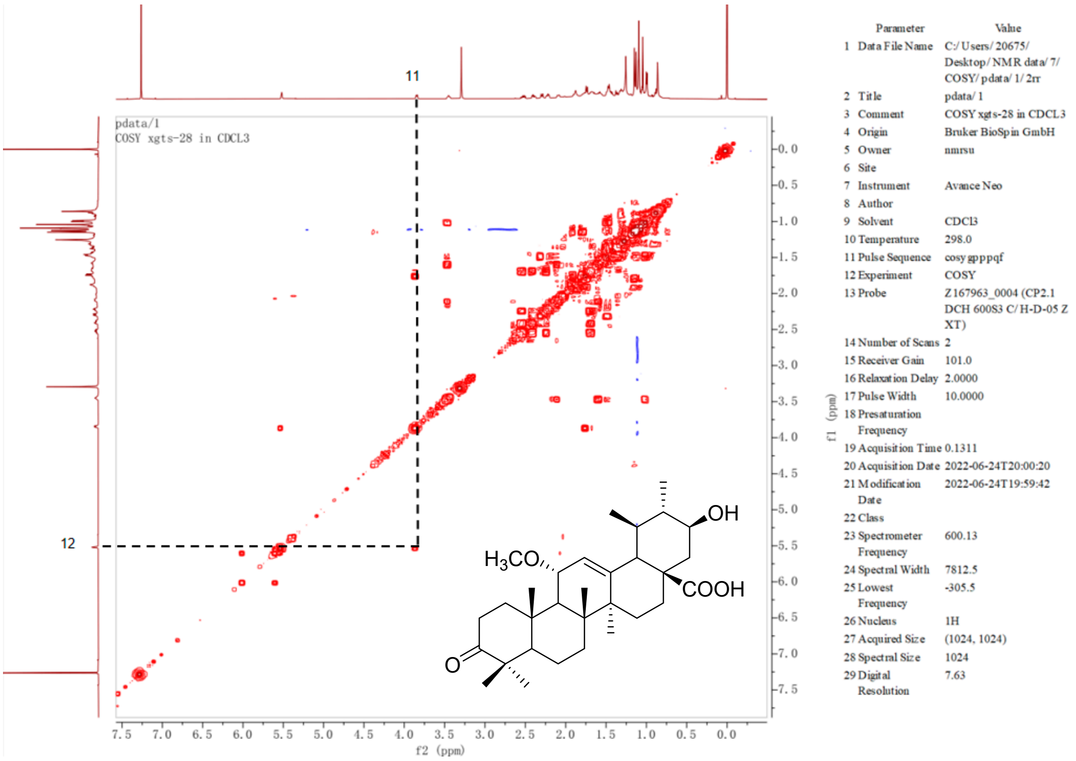


**Figure S21** $^{1}H$-$^{1}H$ COSY spectrum (600 MHz) of compound **7** in $CDCl_3$

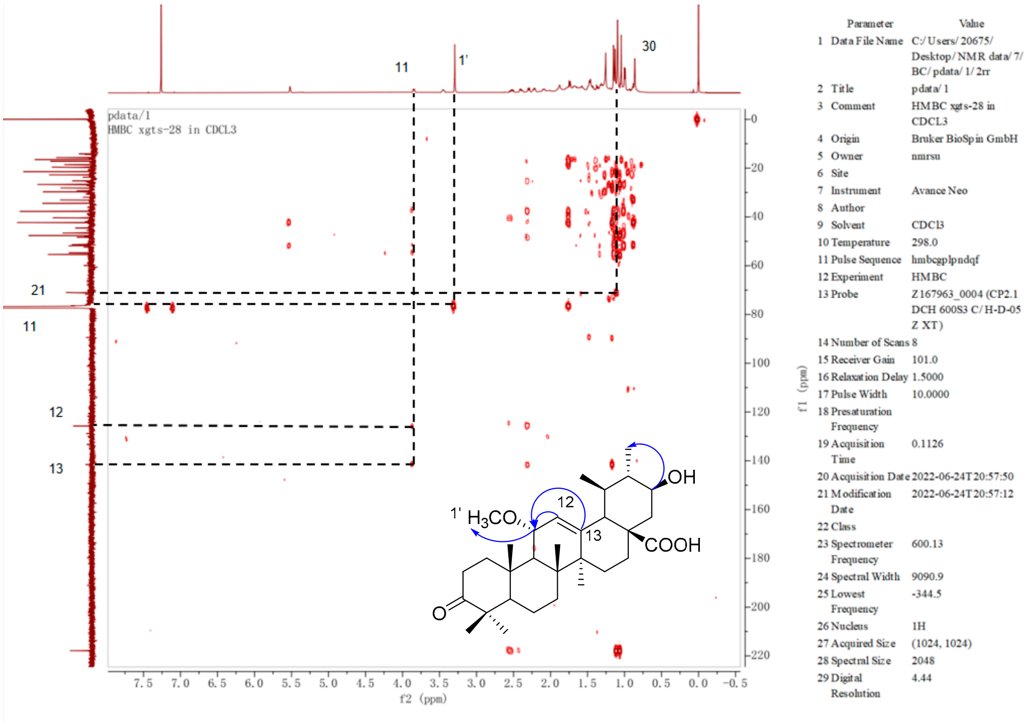


**Figure S22** HMBC spectrum (150 MHz) of compound **7** in $CDCl_3$

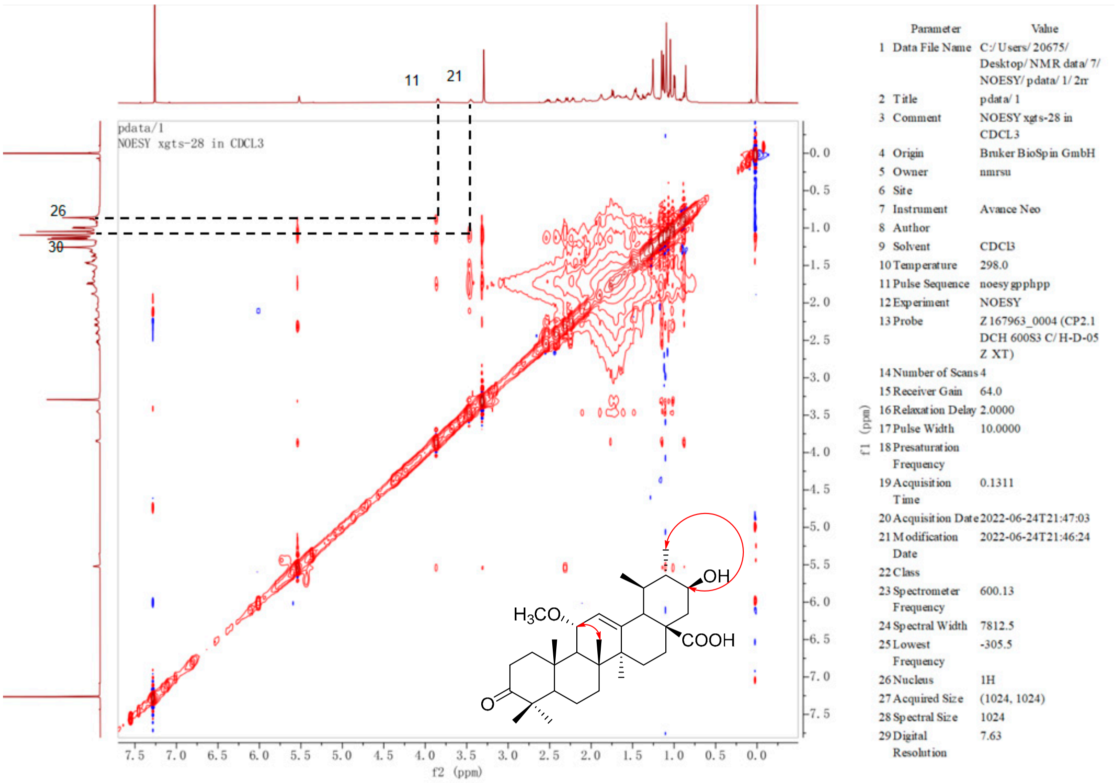


**Figure S23** NOESY spectrum (600 MHz) of compound **7** in $CDCl_3$

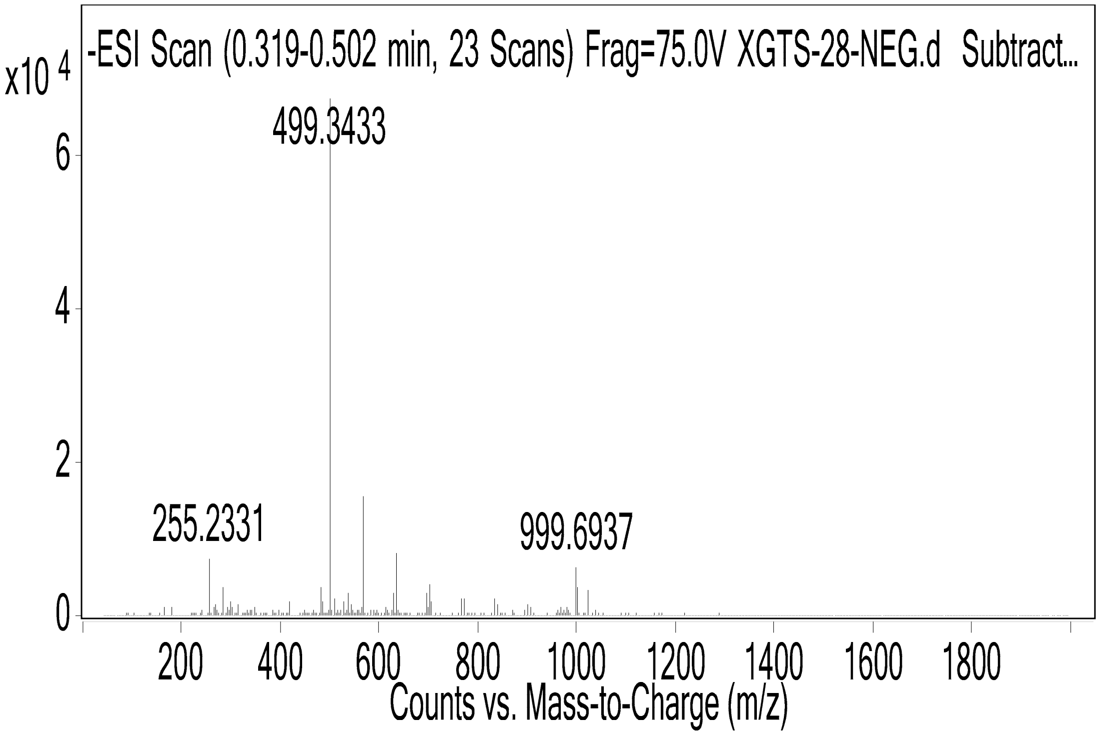


**Figure S24** HR-ESI-MS spectrum of compound **7**

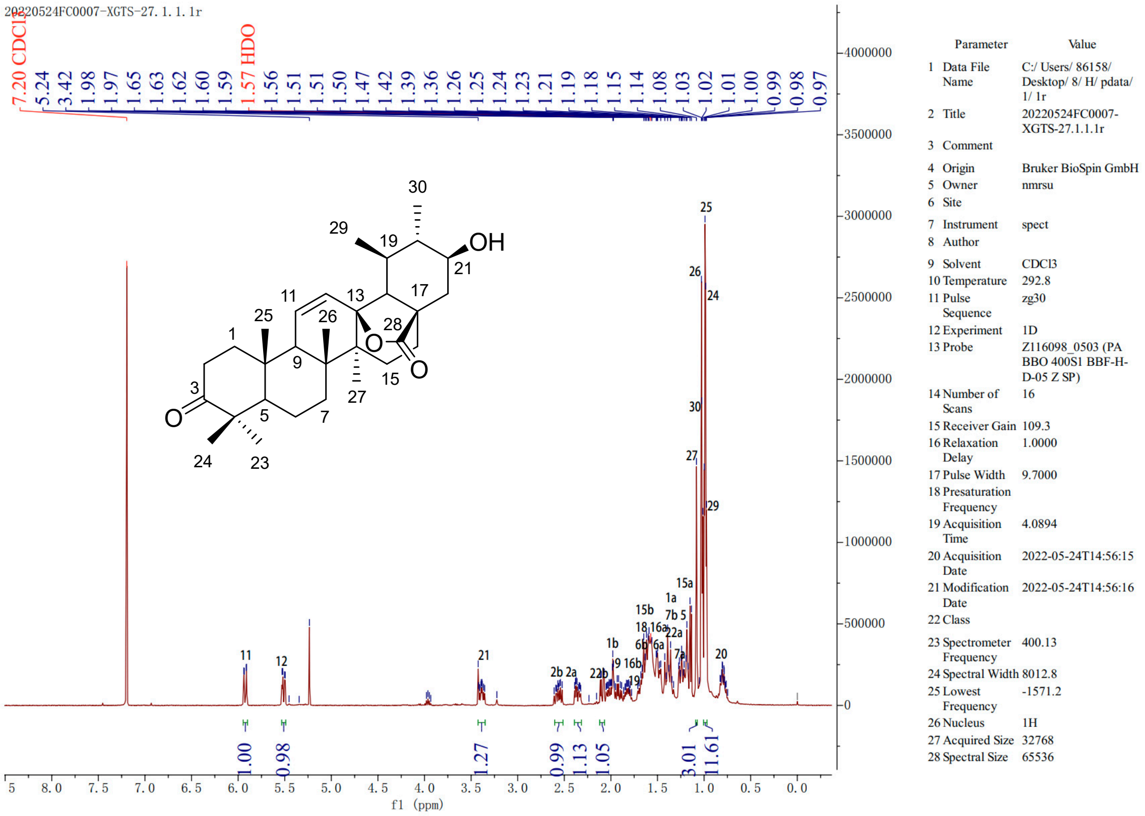


**Figure S25** $^{1}H$ NMR spectrum (400 MHz) of compound **8** in $CDCl_3$

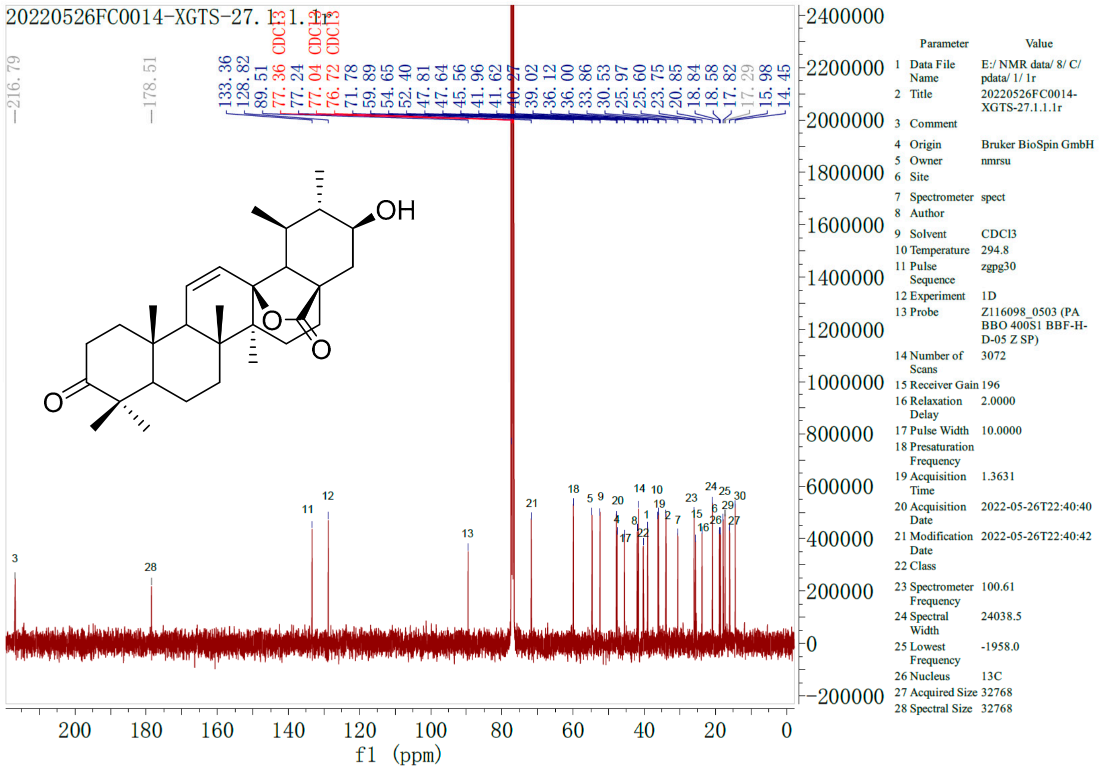


**Figure S26** $^{13}C$ NMR spectrum (100 MHz) of compound **8** in $CDCl_3$

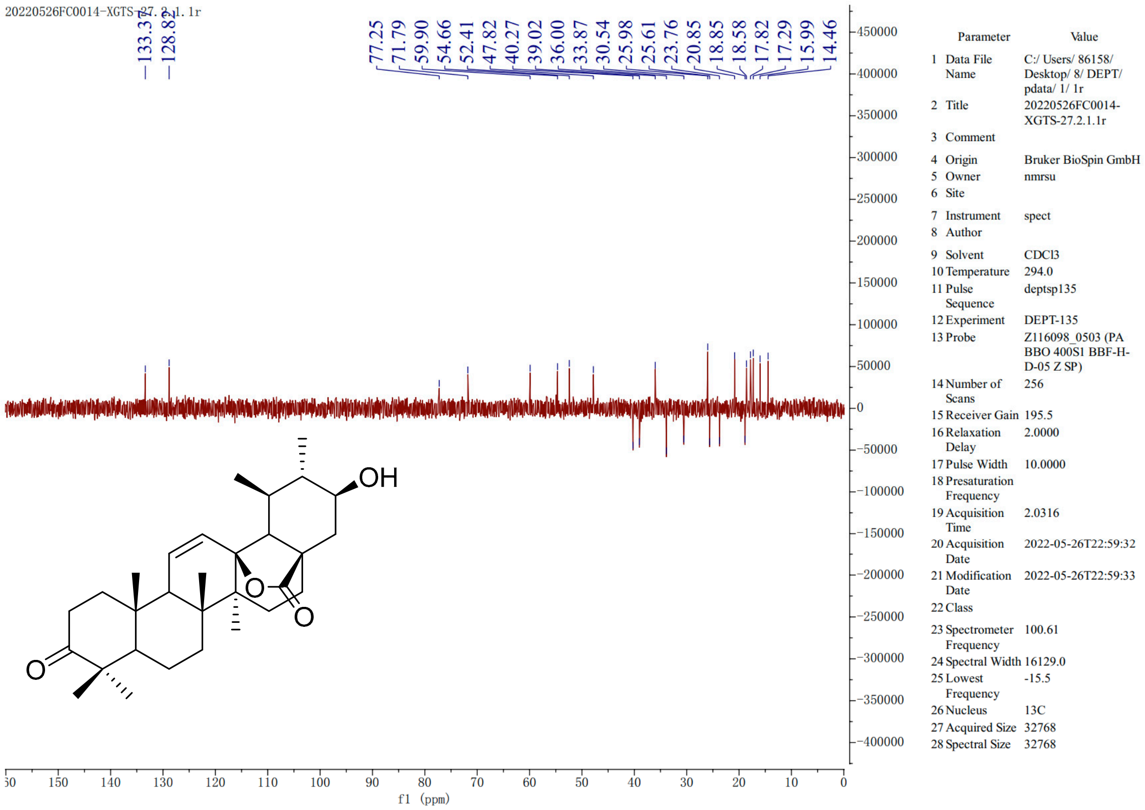


**Figure S27** DEPT-135 spectrum (100 MHz) of compound **8** in $CDCl_3$

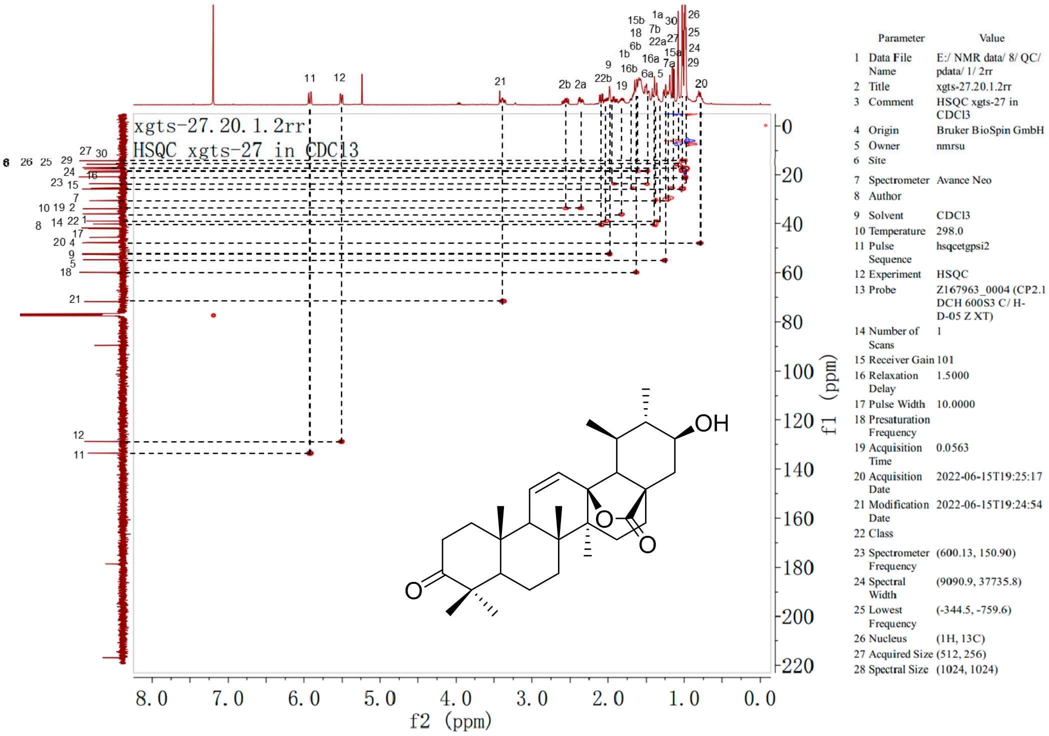


**Figure S28** HSQC spectrum (100 MHz) of compound **8** in $CDCl_3$

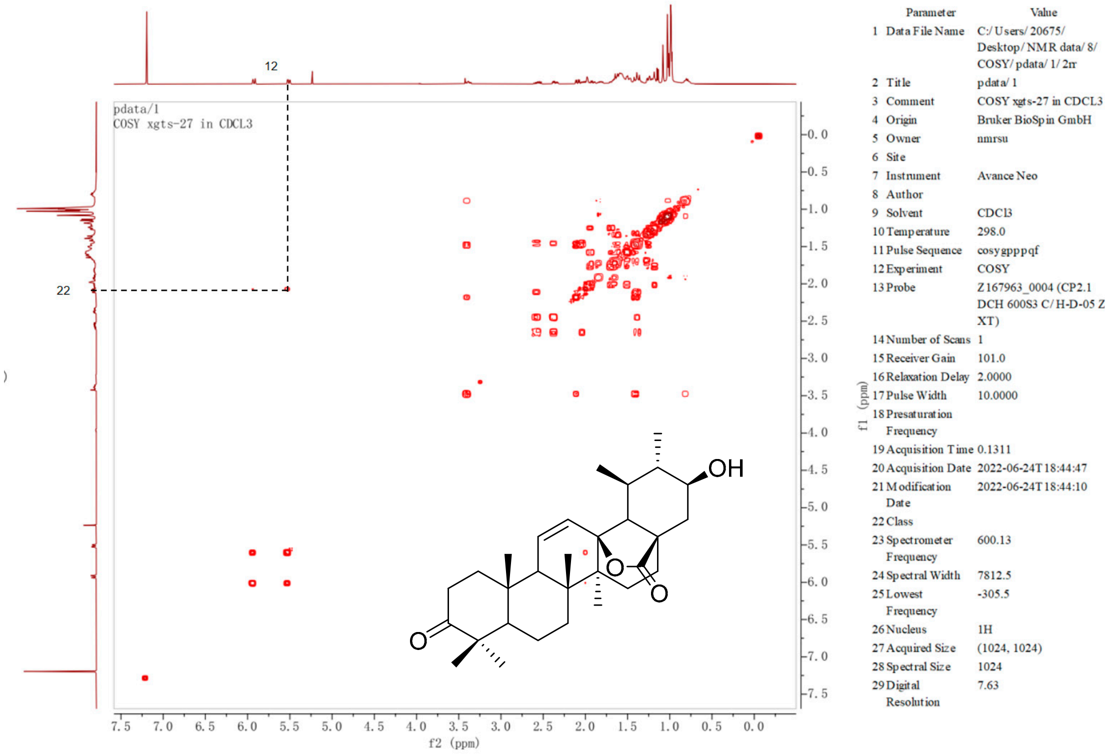


**Figure S29** $^{1}H$-$^{1}H$ COSY spectrum (400 MHz) of compound **8** in $CDCl_3$

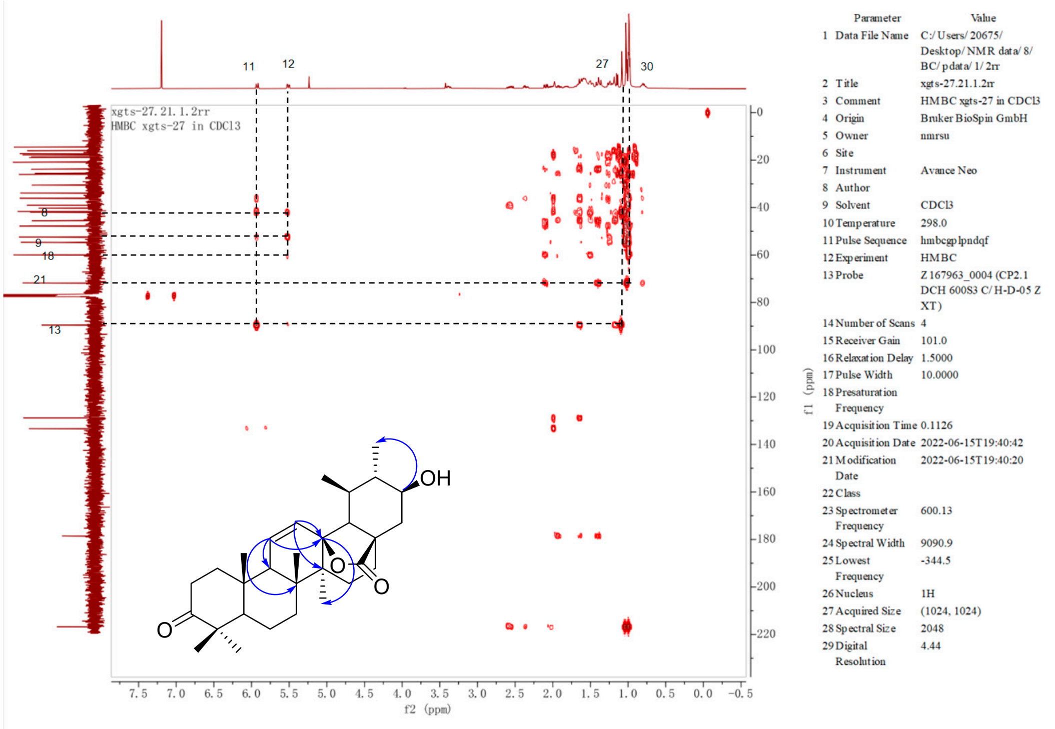


**Figure S30** HMBC spectrum (100 MHz) of compound **8** in $CDCl_3$

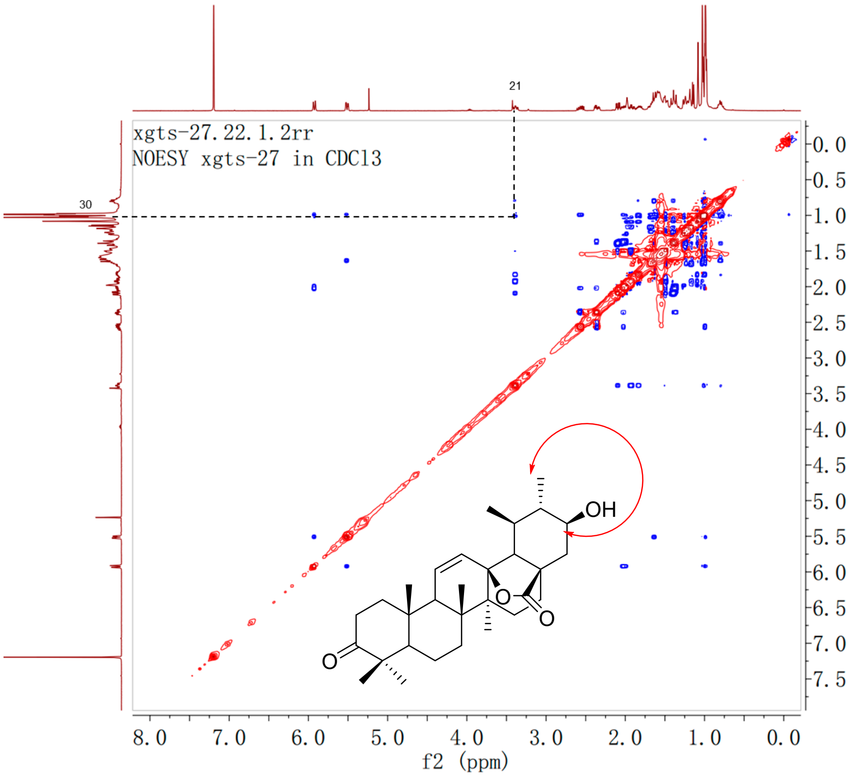


| | Parameter | Value |
|---|---|---|
| 1 | Data File Name | E:/ NMR data/ 8/ NOESY/ pdata/ 1/ 2rr |
| 2 | Title | xgts-27.22.1.2rr |
| 3 | Comment | NOESY xgts-27 in CDCl3 |
| 4 | Origin | Bruker BioSpin GmbH |
| 5 | Owner | nmrsu |
| 6 | Site | |
| 7 | Spectrometer | Avance Neo |
| 8 | Author | |
| 9 | Solvent | CDCl3 |
| 10 | Temperature | 298.0 |
| 11 | Pulse Sequence | noesygpphpp |
| 12 | Experiment | NOESY |
| 13 | Probe | Z167963_0004 (CP2.1 DCH 600S3 C/ H-D-05 Z XT) |
| 14 | Number of Scans | 2 |
| 15 | Receiver Gain | 64 |
| 16 | Relaxation Delay | 2.0000 |
| 17 | Pulse Width | 10.0000 |
| 18 | Presaturation Frequency | |
| 19 | Acquisition Time | 0.1311 |
| 20 | Acquisition Date | 2022-06-15T20:06:18 |
| 21 | Modification Date | 2022-06-15T20:05:56 |
| 22 | Class | |
| 23 | Spectrometer Frequency | (600.13, 600.13) |
| 24 | Spectral Width | (7812.5, 7801.8) |
| 25 | Lowest Frequency | (-305.5, -300.1) |
| 26 | Nucleus | (1H, 1H) |
| 27 | Acquired Size | (1024, 256) |
| 28 | Spectral Size | (1024, 1024) |

**Figure S31** NOESY spectrum (400 MHz) of compound **8** in $CDCl_3$

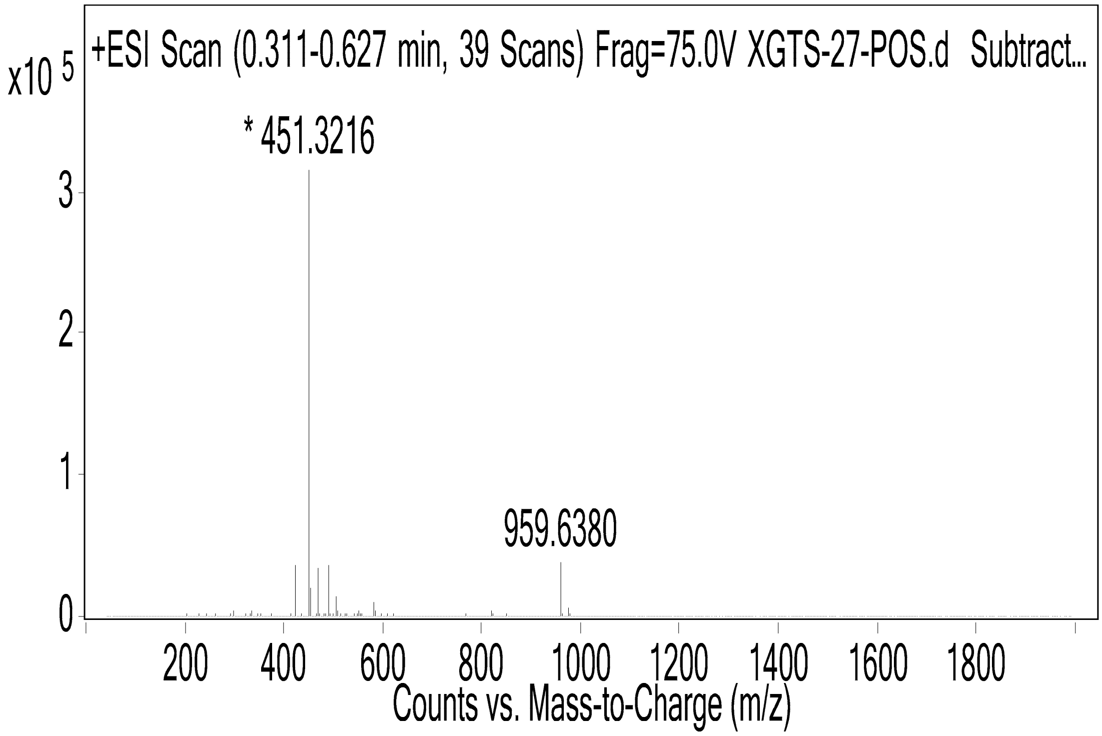


**Figure S32** HR-ESI-MS spectrum of compound **8**

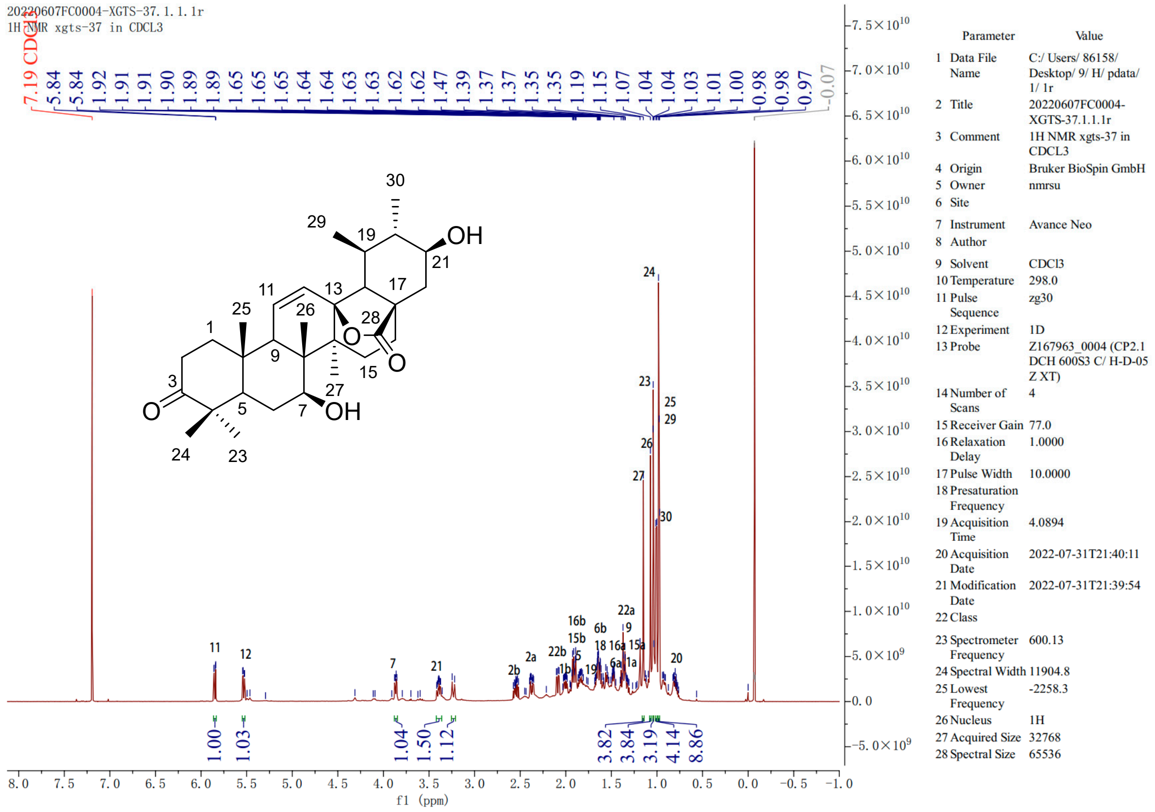

**Figure S33** $^{1}$H NMR spectrum (400 MHz) of compound **9** in $CDCl_3$

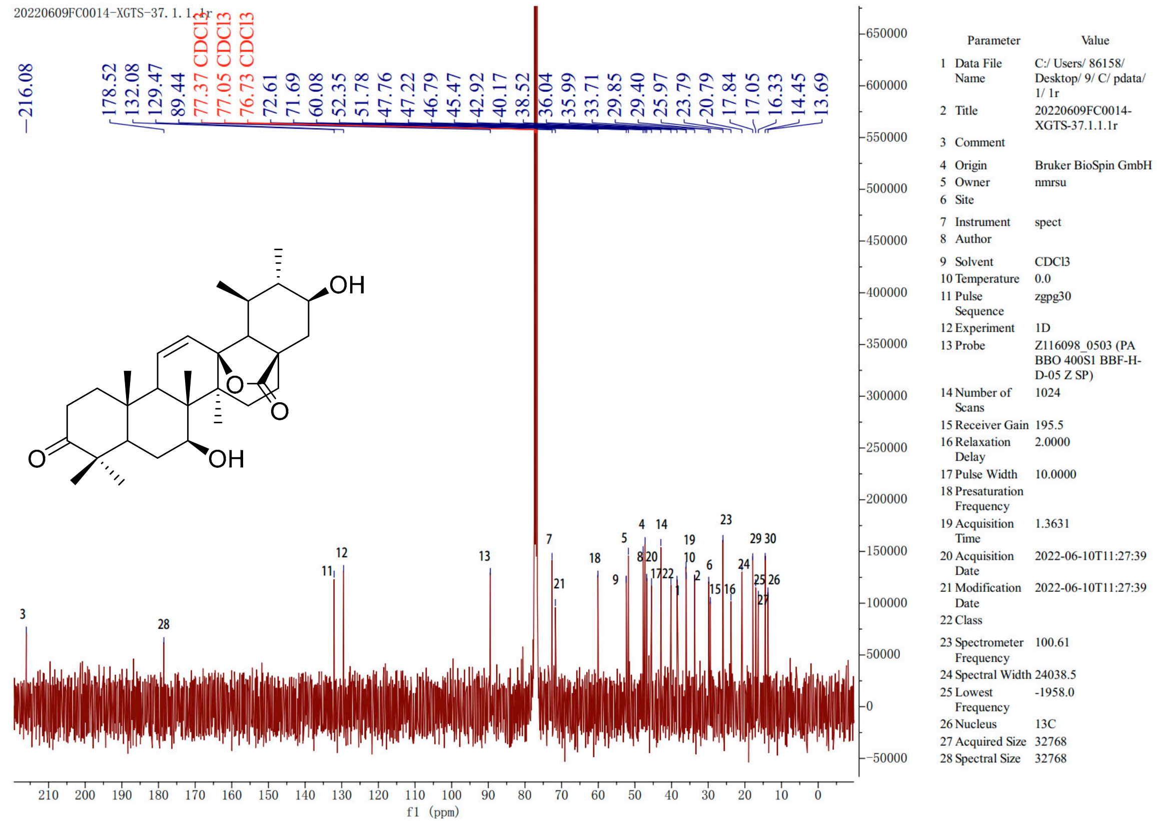

**Figure S34** $^{13}$C NMR spectrum (100 MHz) of compound **9** in $CDCl_3$

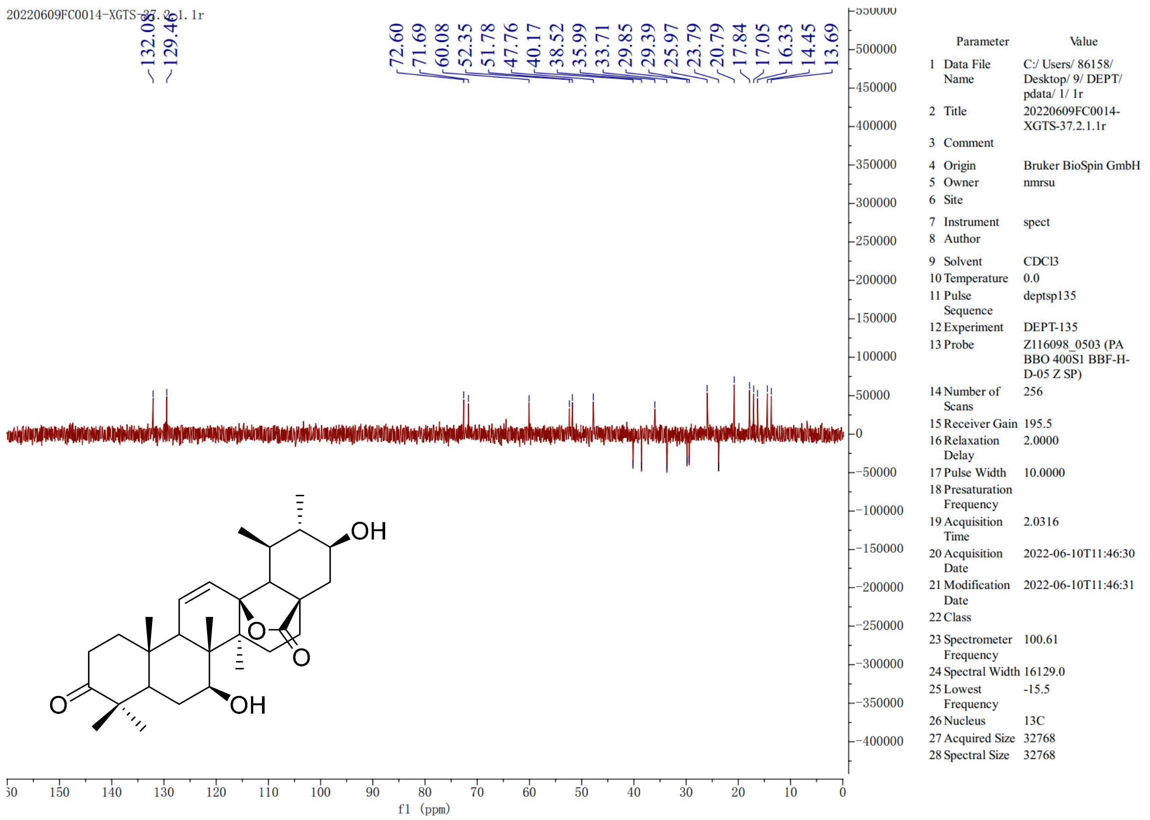


**Figure S35** DEPT-135 spectrum (100 MHz) of compound **9** in $CDCl_3$

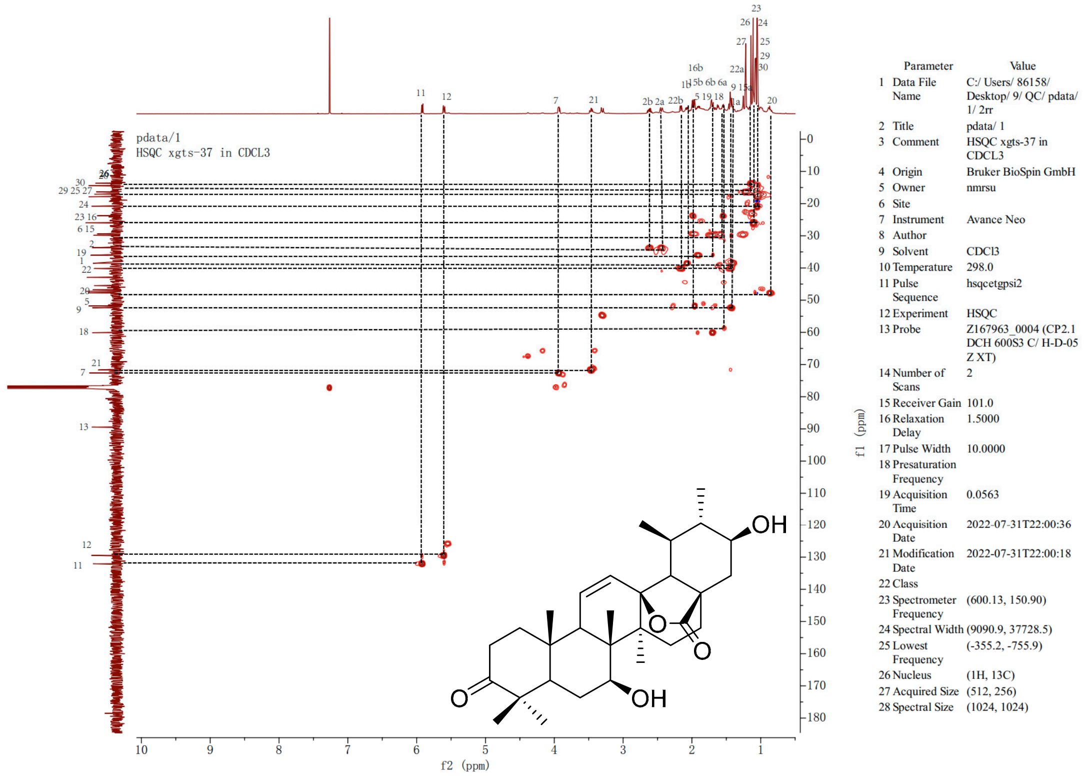


**Figure S36** HSQC spectrum (100 MHz) of compound **9** in $CDCl_3$

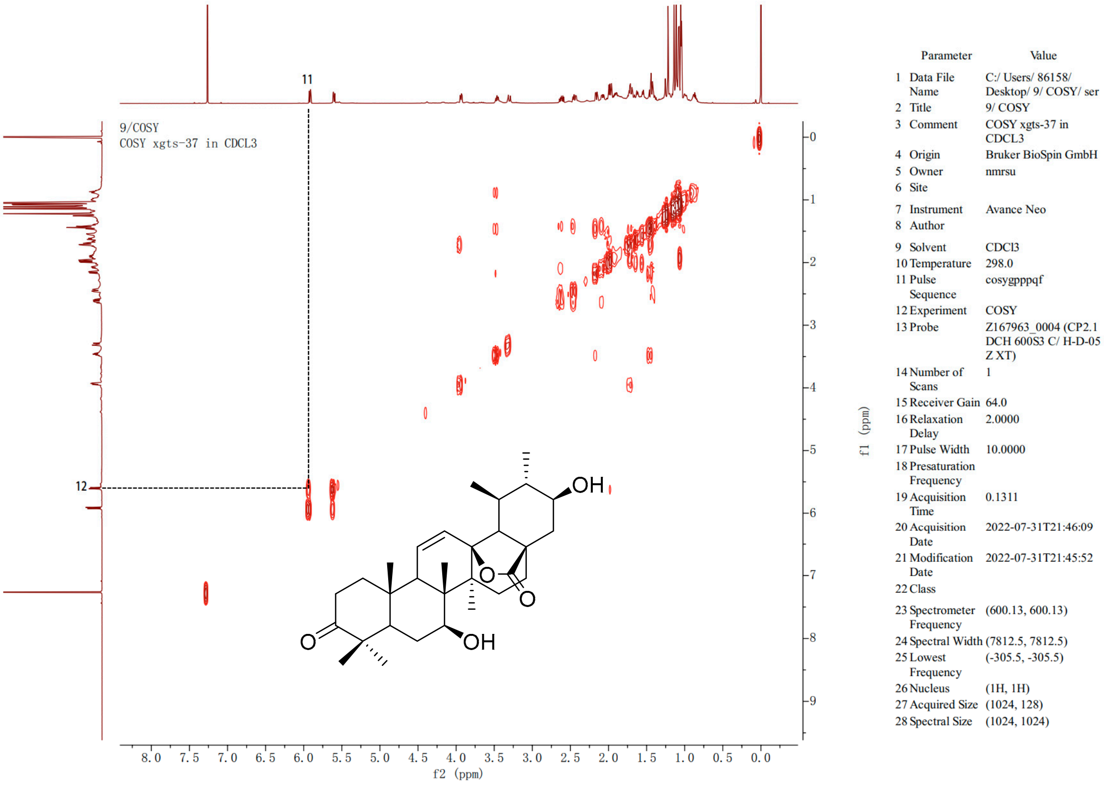


**Figure S37** $^{1}H$-$^{1}H$ COSY spectrum (400 MHz) of compound **9** in $CDCl_3$

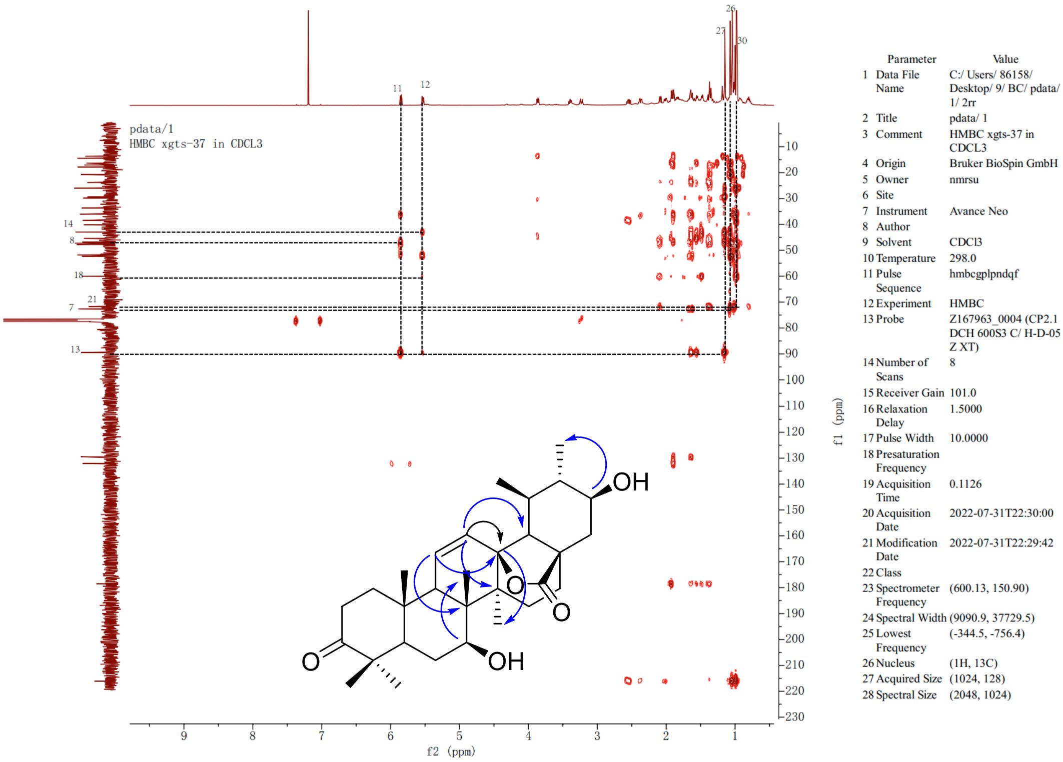


**Figure S38** HMBC spectrum (100 MHz) of compound **9** in $CDCl_3$

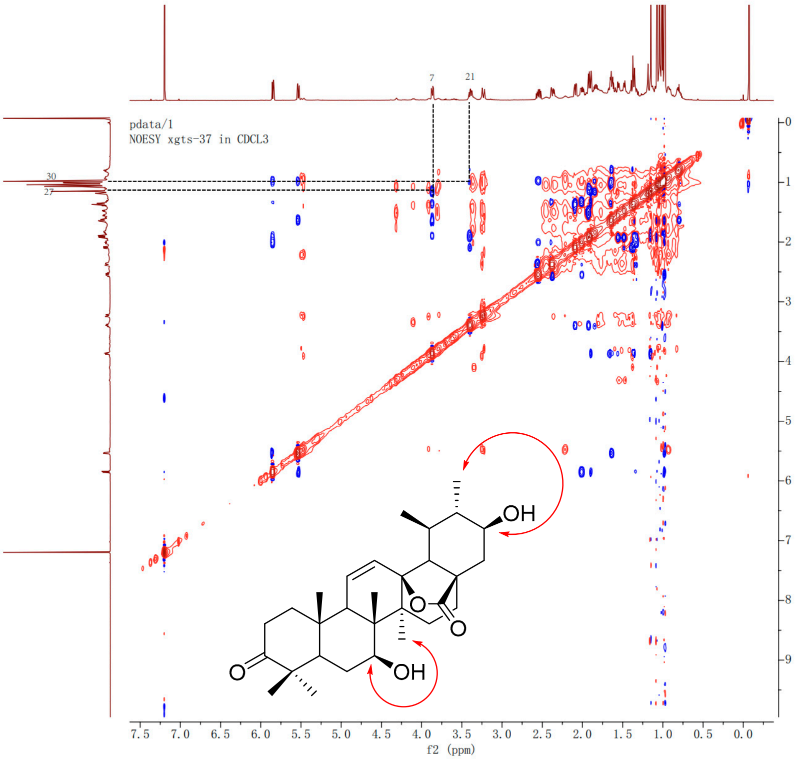


| Parameter | | Value |
|---|---|---|
| 1 | Data File Name | C:/ Users/ 86158/ Desktop/ 9/ NOESY/ pdata/ 1/ 2rr |
| 2 | Title | pdata/ 1 |
| 3 | Comment | NOESY xgts-37 in CDCL3 |
| 4 | Origin | Bruker BioSpin GmbH |
| 5 | Owner | nmrsu |
| 6 | Site | |
| 7 | Instrument | Avance Neo |
| 8 | Author | |
| 9 | Solvent | CDCl3 |
| 10 | Temperature | 298.0 |
| 11 | Pulse Sequence | noesygpphpp |
| 12 | Experiment | NOESY |
| 13 | Probe | Z167963_0004 (CP2.1 DCH 600S3 C/ H-D-05 Z XT) |
| 14 | Number of Scans | 2 |
| 15 | Receiver Gain | 64.0 |
| 16 | Relaxation Delay | 2.0000 |
| 17 | Pulse Width | 10.0000 |
| 18 | Presaturation Frequency | |
| 19 | Acquisition Time | 0.1311 |
| 20 | Acquisition Date | 2022-07-31T22:55:32 |
| 21 | Modification Date | 2022-07-31T22:55:14 |
| 22 | Class | |
| 23 | Spectrometer Frequency | (600.13, 600.13) |
| 24 | Spectral Width | (7812.5, 7801.7) |
| 25 | Lowest Frequency | (-305.5, -300.1) |
| 26 | Nucleus | (1H, 1H) |
| 27 | Acquired Size | (1024, 256) |
| 28 | Spectral Size | (1024, 1024) |

**Figure S39** NOESY spectrum (400 MHz) of compound **9** in $CDCl_3$

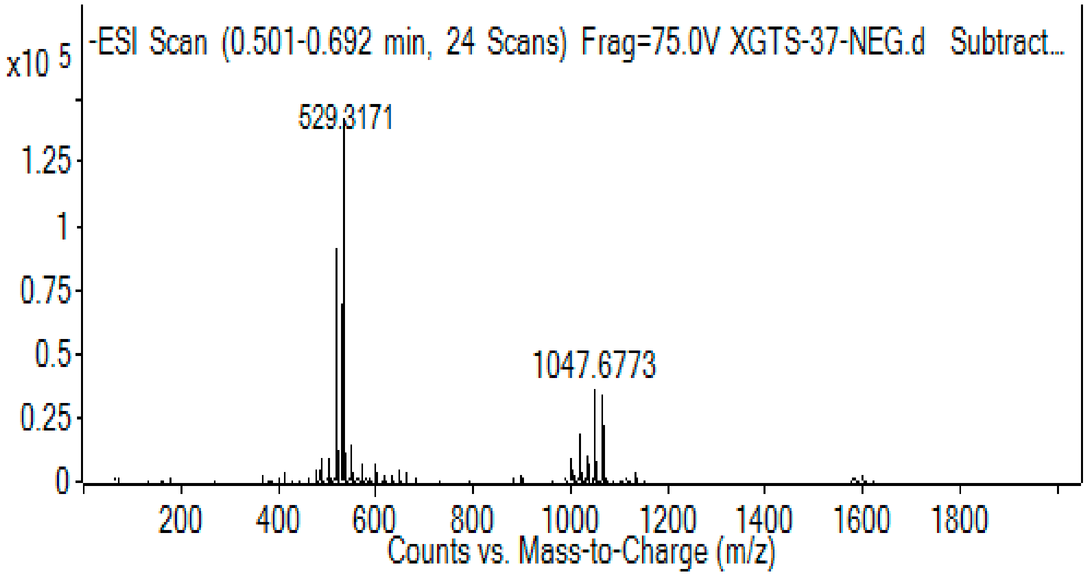


**Figure S40** HR-ESI-MS spectrum of compound **9**

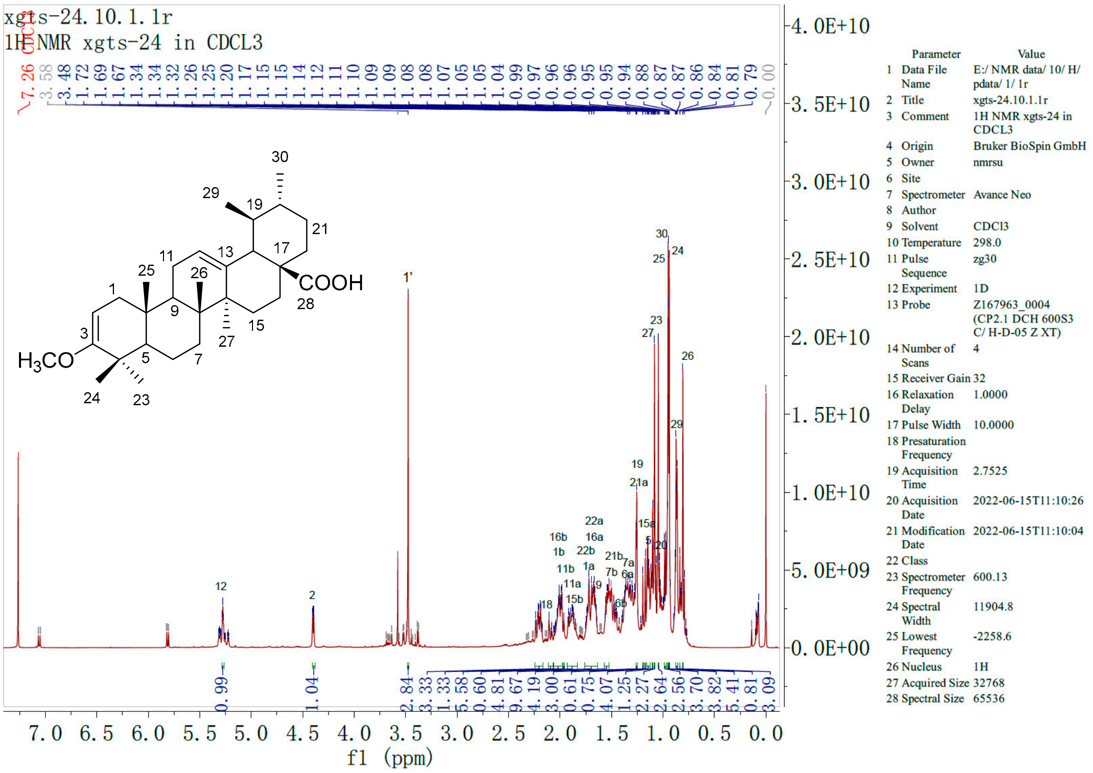


**Figure S41** $^{1}$H NMR spectrum (400 MHz) of compound **10** in $CDCl_3$

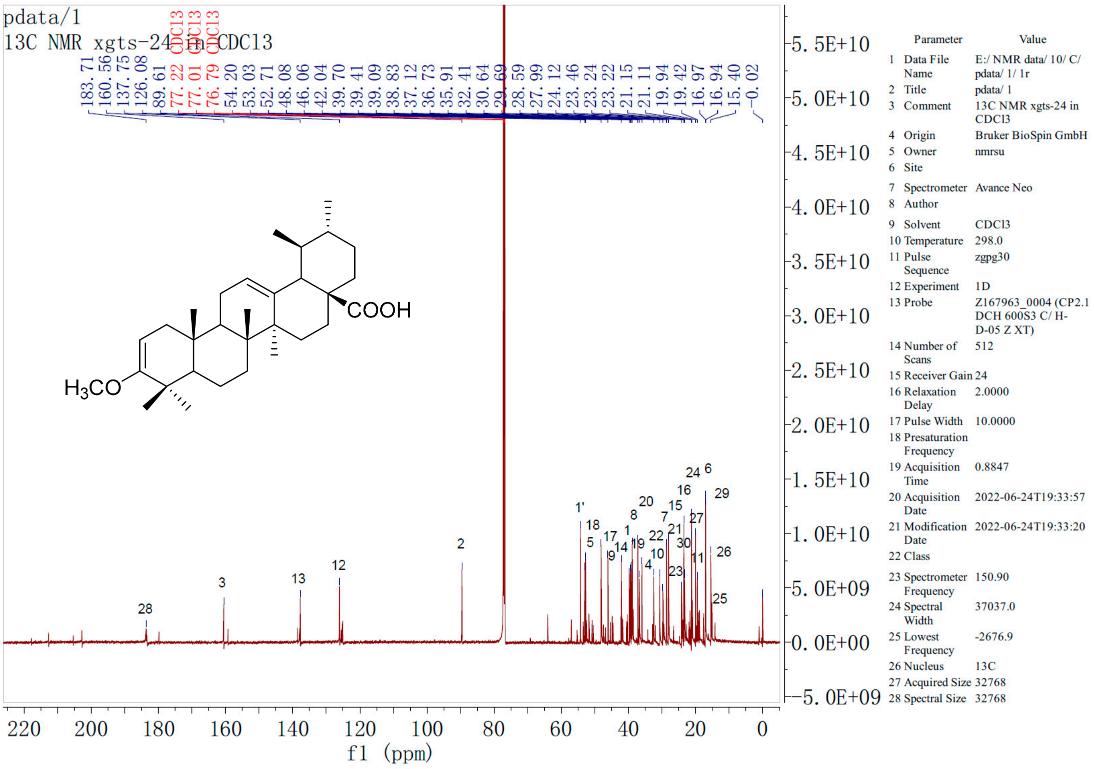


**Figure S42** $^{13}$C NMR spectrum (100 MHz) of compound **10** in $CDCl_3$

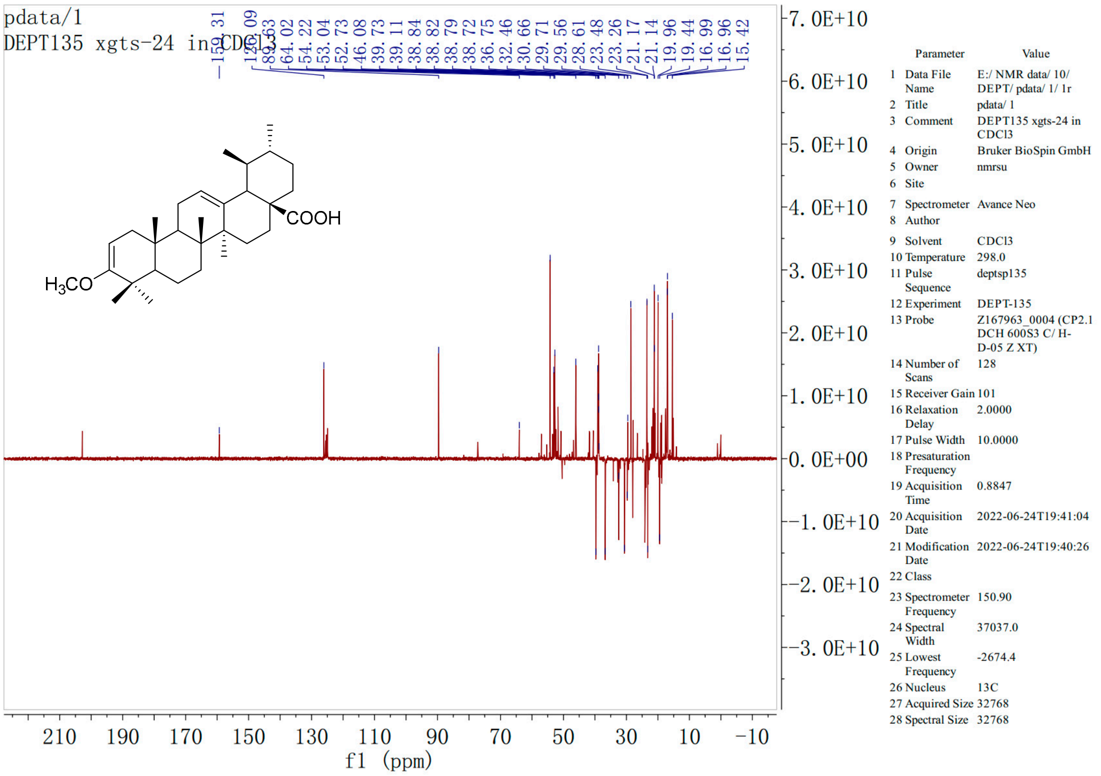


**Figure S43** DEPT-135 spectrum (100 MHz) of compound **10** in $CDCl_3$

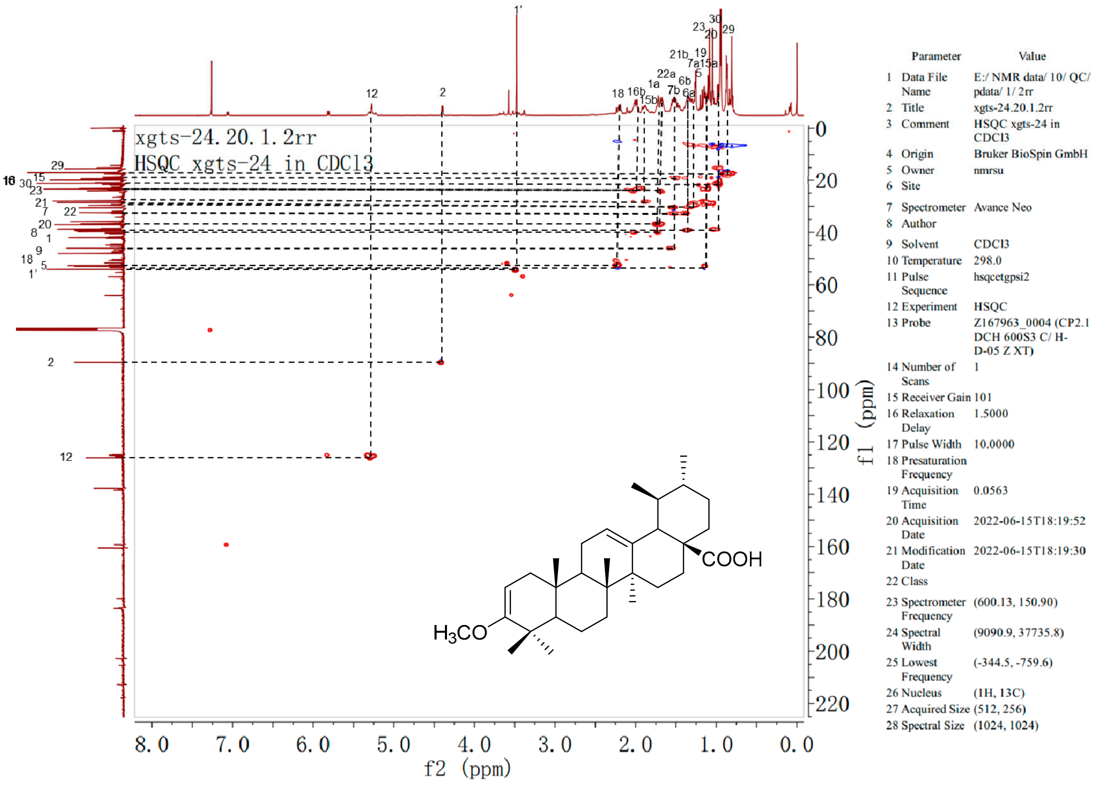


**Figure S44** HSQC spectrum (100 MHz) of compound **10** in $CDCl_3$

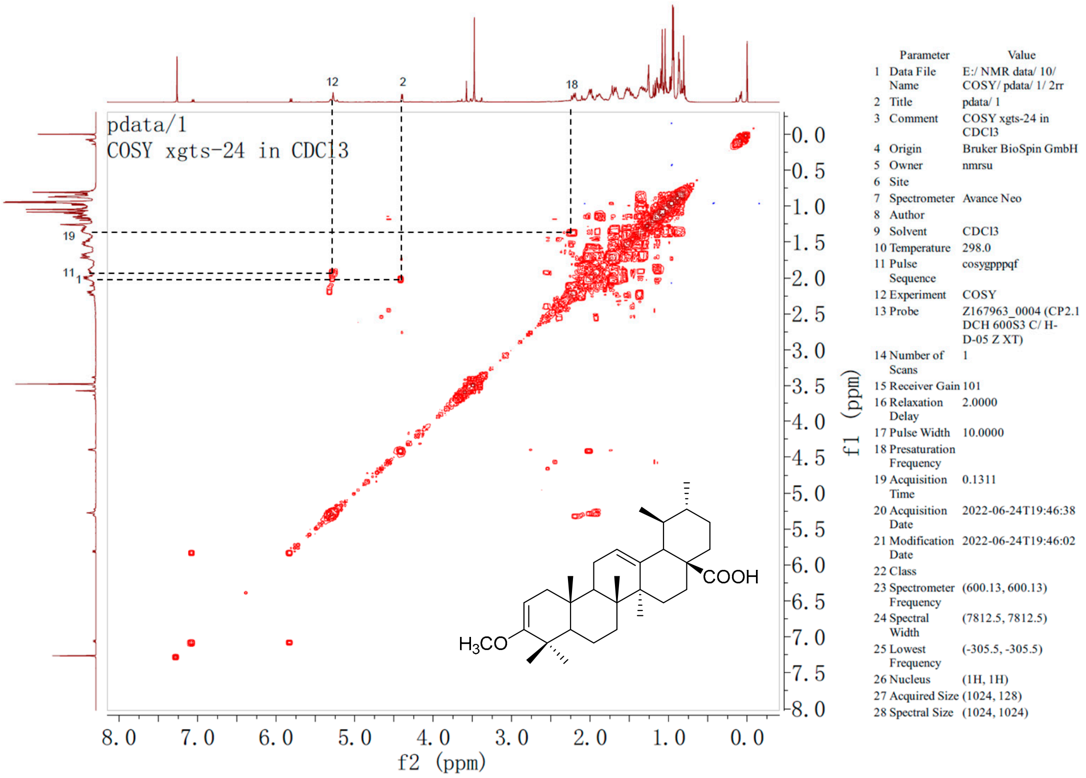


**Figure S45** $^{1}H$-$^{1}H$ COSY spectrum (400 MHz) of compound **10** in $CDCl_3$

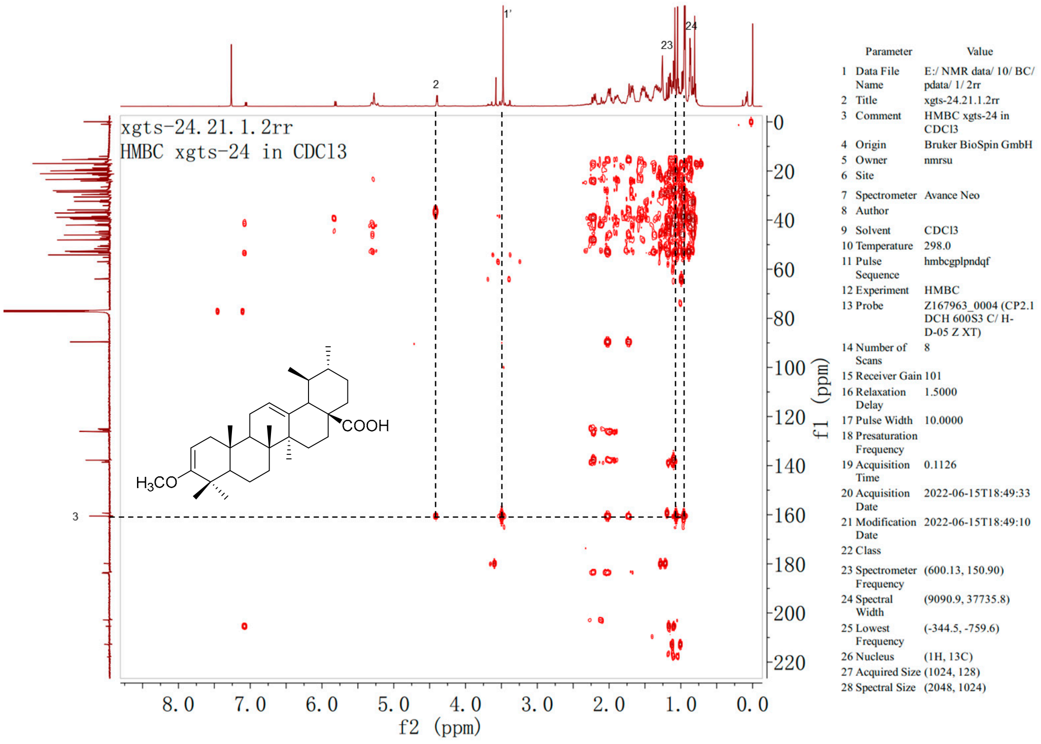


**Figure S46** HMBC spectrum (100 MHz) of compound **10** in $CDCl_3$

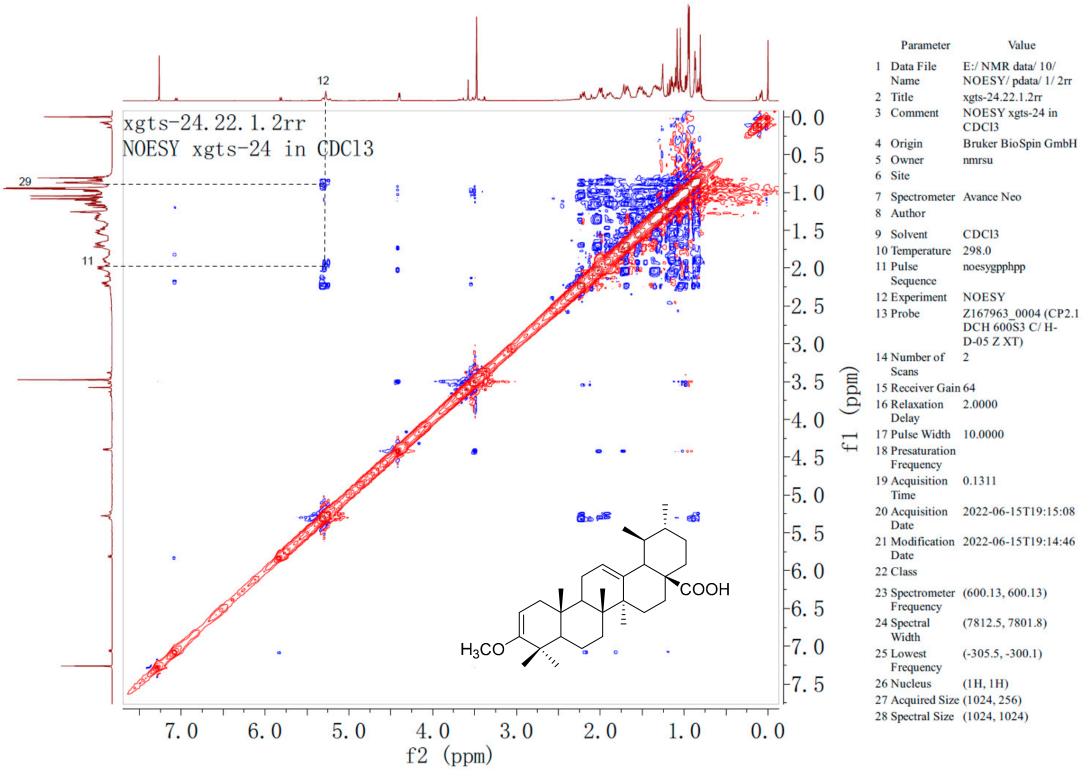


**Figure S47** NOESY spectrum (400 MHz) of compound **10** in $CDCl_3$

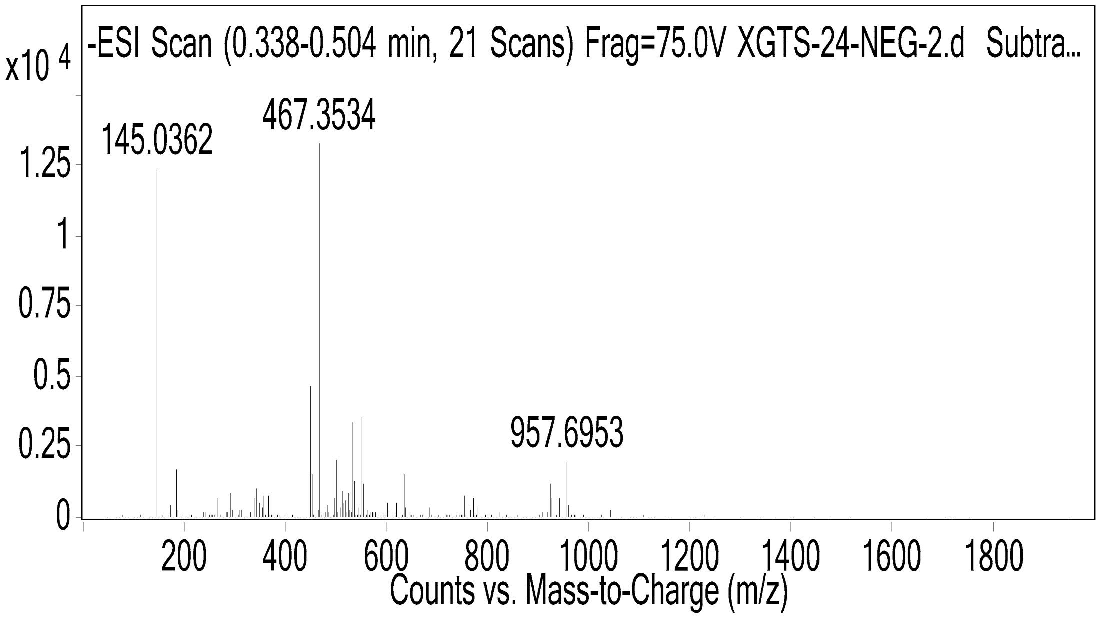


**Figure S48** HR-ESI-MS spectrum of compound **10**

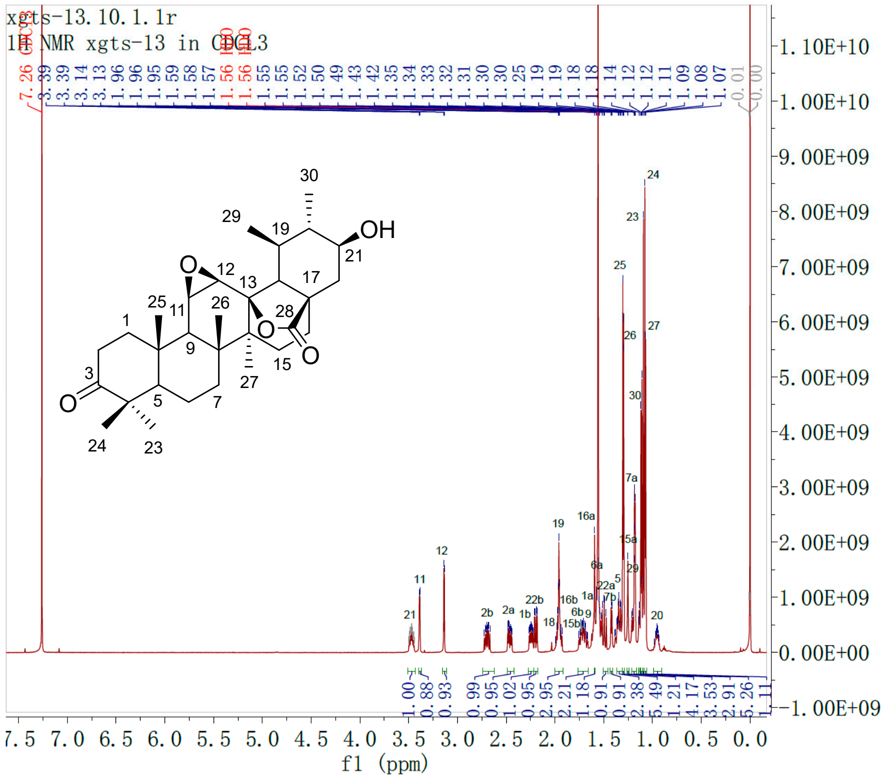


| Parameter | | Value |
|---|---|---|
| 1 | Data File Name | E:/ NMR data/ 13/ H/ pdata/ 1/ 1r |
| 2 | Title | xgts-13.10.1.1r |
| 3 | Comment | 1H NMR xgts-13 in CDCL3 |
| 4 | Origin | Bruker BioSpin GmbH |
| 5 | Owner | nmrsu |
| 6 | Site | |
| 7 | Spectrometer | Avance Neo |
| 8 | Author | |
| 9 | Solvent | CDCl3 |
| 10 | Temperature | 298.0 |
| 11 | Pulse Sequence | zg30 |
| 12 | Experiment | 1D |
| 13 | Probe | Z167963_0004 (CP2.1 DCH 600S3 C/ H-D-05 Z XT) |
| 14 | Number of Scans | 4 |
| 15 | Receiver Gain | 32 |
| 16 | Relaxation Delay | 1.0000 |
| 17 | Pulse Width | 10.0000 |
| 18 | Presaturation Frequency | |
| 19 | Acquisition Time | 2.7525 |
| 20 | Acquisition Date | 2022-06-15T11:19:55 |
| 21 | Modification Date | 2022-06-15T11:19:34 |
| 22 | Class | |
| 23 | Spectrometer Frequency | 600.13 |
| 24 | Spectral Width | 11904.8 |
| 25 | Lowest Frequency | -2259.7 |
| 26 | Nucleus | 1H |
| 27 | Acquired Size | 32768 |
| 28 | Spectral Size | 65536 |

**Figure S49** $^{1}$H NMR spectrum (400 MHz) of compound **13** in $CDCl_3$

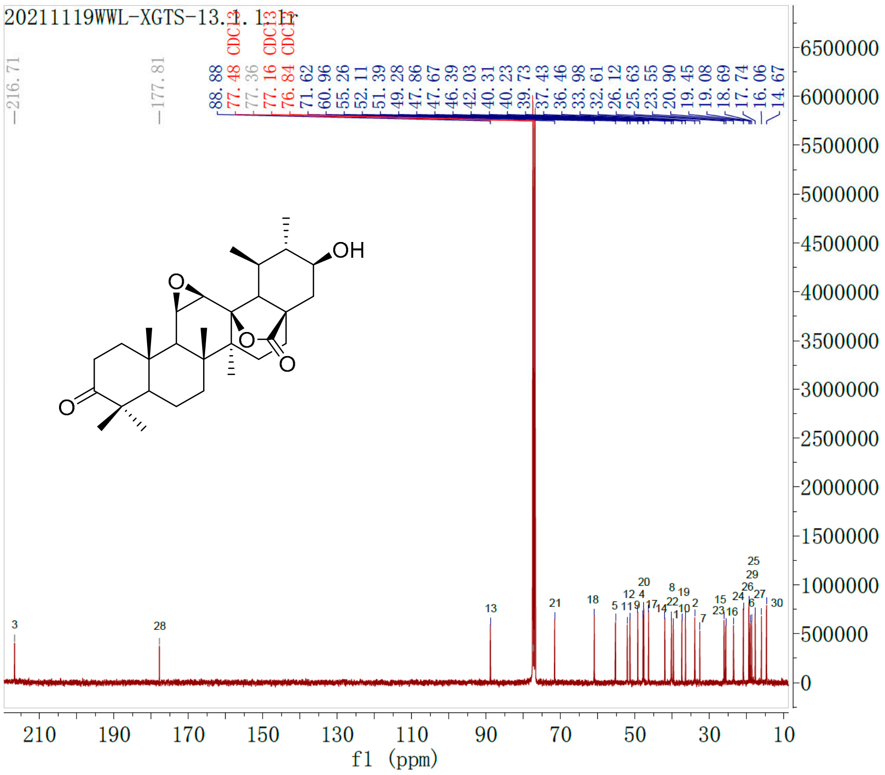


| Parameter | | Value |
|---|---|---|
| 1 | Data File Name | E:/ NMR data/ 13/ C/ pdata/ 1/ 1r |
| 2 | Title | 20211119WWL-XGTS-13.1.1.1r |
| 3 | Comment | |
| 4 | Origin | Bruker BioSpin GmbH |
| 5 | Owner | nmrsu |
| 6 | Site | |
| 7 | Spectrometer | spect |
| 8 | Author | |
| 9 | Solvent | CDCl3 |
| 10 | Temperature | 297.2 |
| 11 | Pulse Sequence | zgpg30 |
| 12 | Experiment | 1D |
| 13 | Probe | Z116098_0503 (PA BBO 400S1 BBF-H-D-05 Z SP) |
| 14 | Number of Scans | 1024 |
| 15 | Receiver Gain | 196 |
| 16 | Relaxation Delay | 2.0000 |
| 17 | Pulse Width | 10.0000 |
| 18 | Presaturation Frequency | |
| 19 | Acquisition Time | 1.3631 |
| 20 | Acquisition Date | 2021-11-20T10:09:28 |
| 21 | Modification Date | 2021-11-20T10:09:30 |
| 22 | Class | |
| 23 | Spectrometer Frequency | 100.61 |
| 24 | Spectral Width | 24038.5 |
| 25 | Lowest Frequency | -1946.1 |
| 26 | Nucleus | 13C |
| 27 | Acquired Size | 32768 |
| 28 | Spectral Size | 32768 |

**Figure S50** $^{13}$C NMR spectrum (100 MHz) of compound **13** in $CDCl_3$

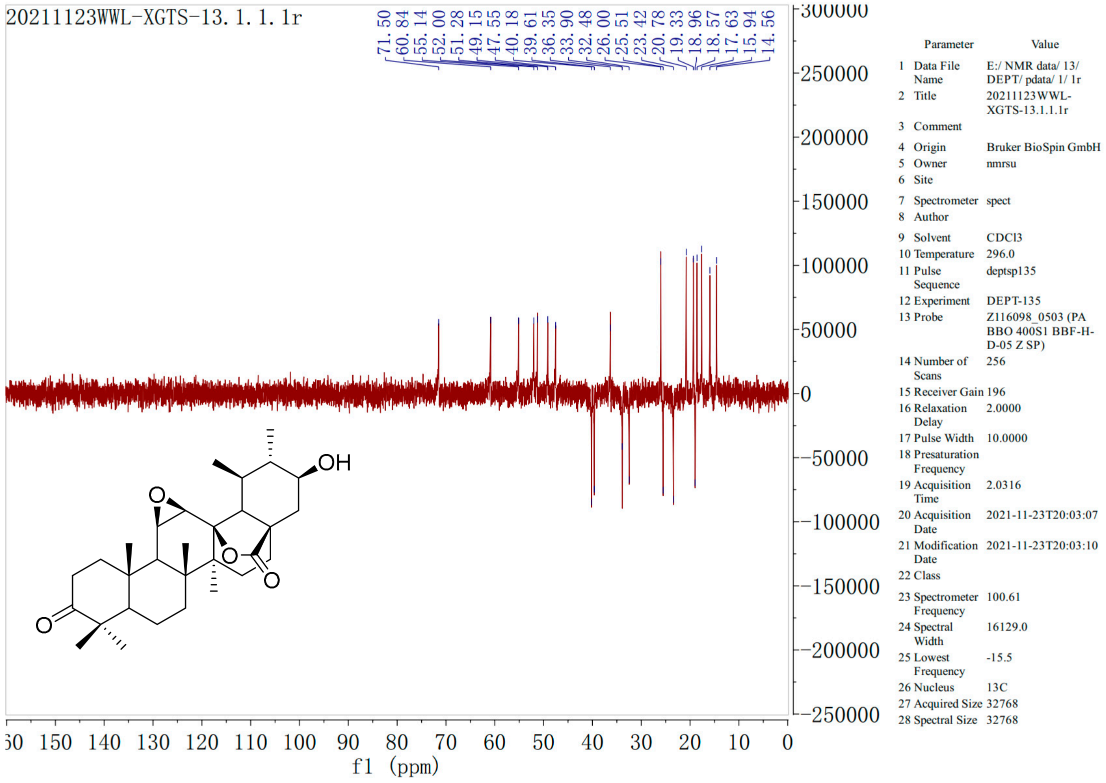


**Figure S51** DEPT-135 spectrum (100 MHz) of compound **13** in $CDCl_3$

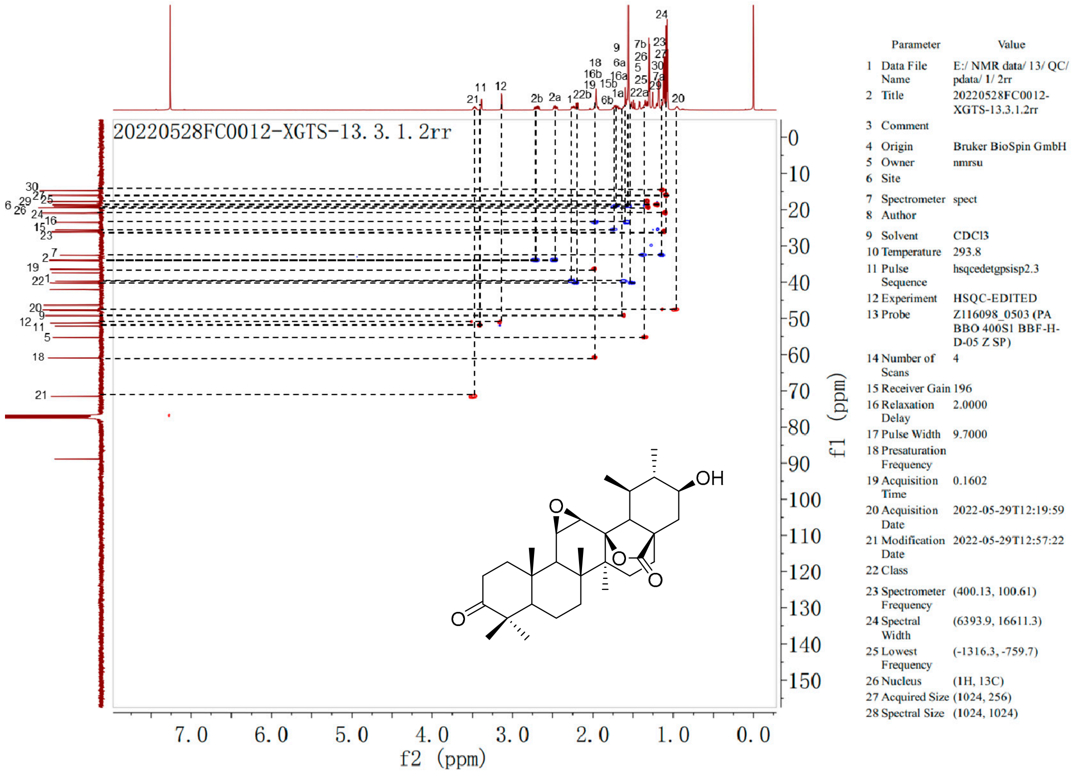


**Figure S52** HSQC spectrum (100 MHz) of compound **13** in $CDCl_3$

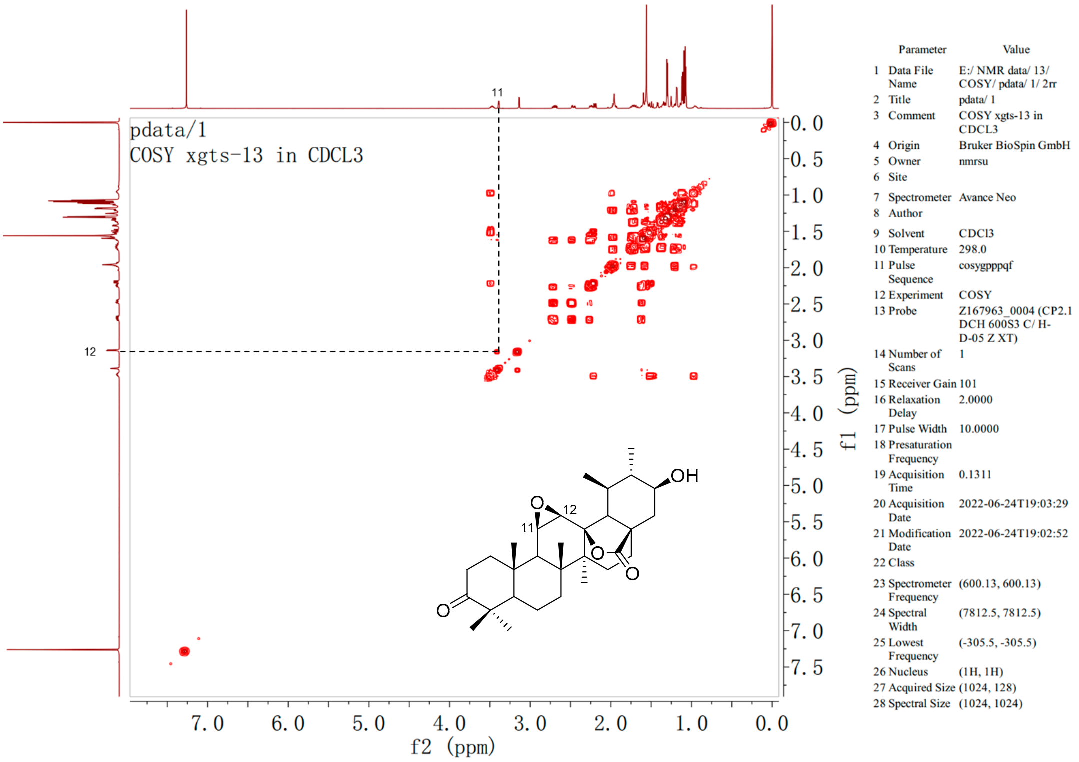


**Figure S53** $^{1}H$-$^{1}H$ COSY spectrum (400 MHz) of compound **13** in $CDCl_3$

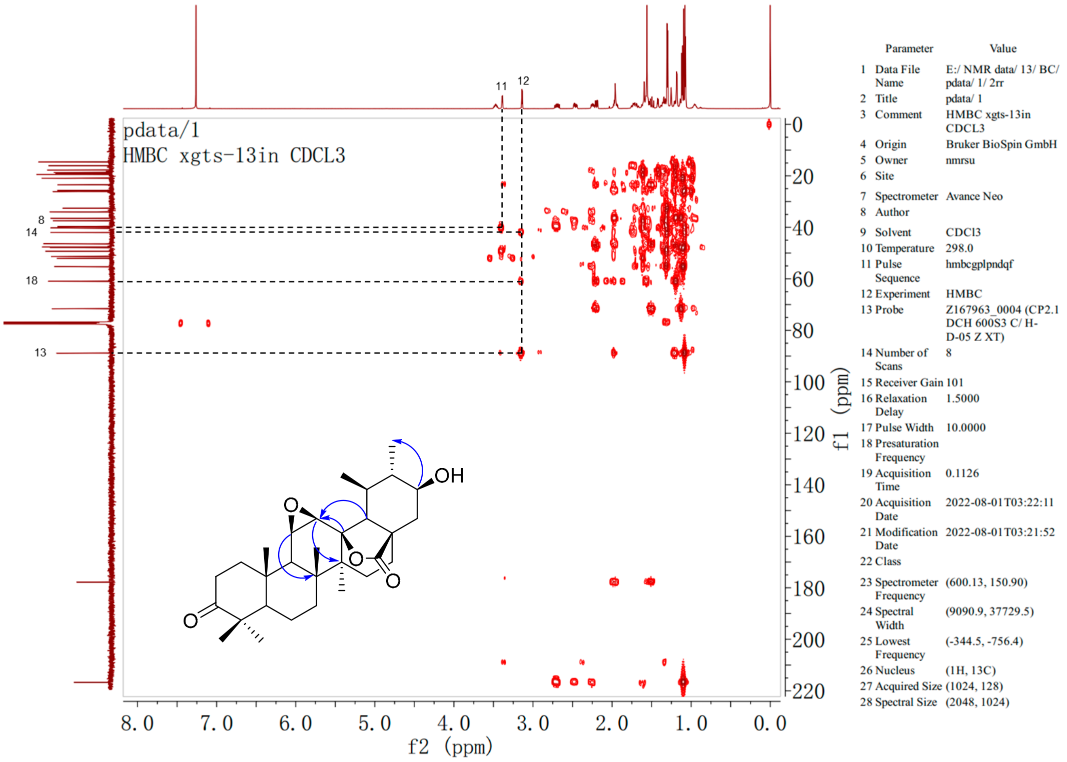


**Figure S54** HMBC spectrum (100 MHz) of compound **13** in $CDCl_3$

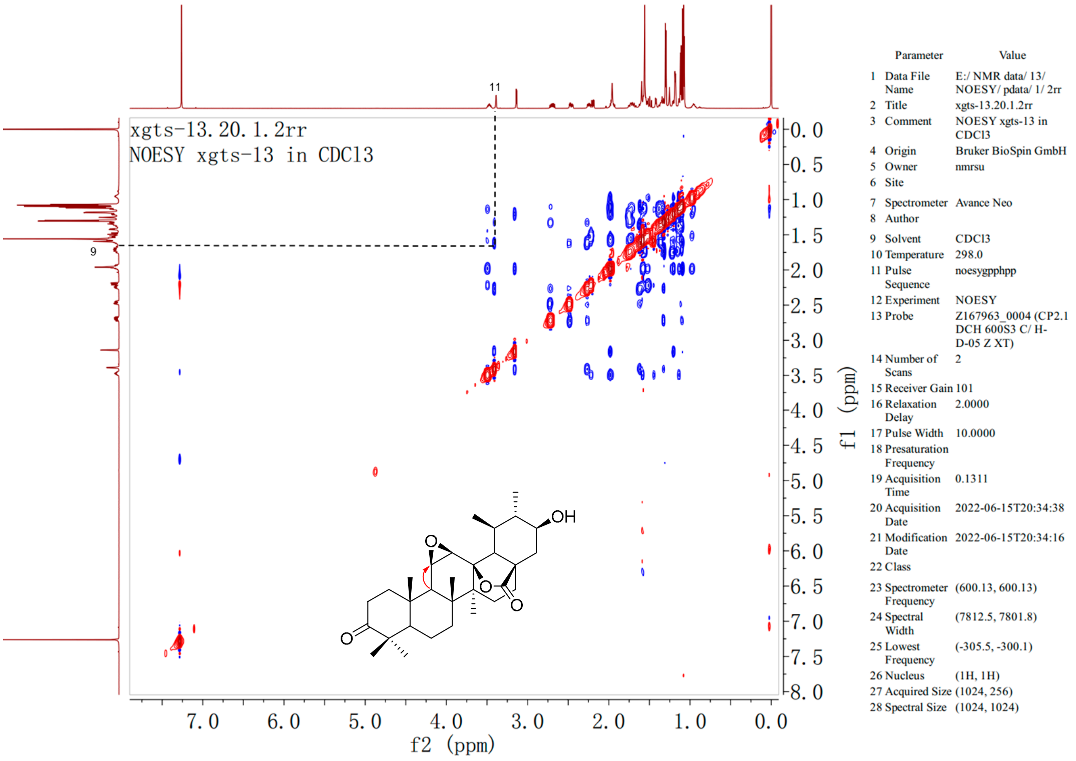


**Figure S55** NOESY spectrum (400 MHz) of compound **13** in $CDCl_3$

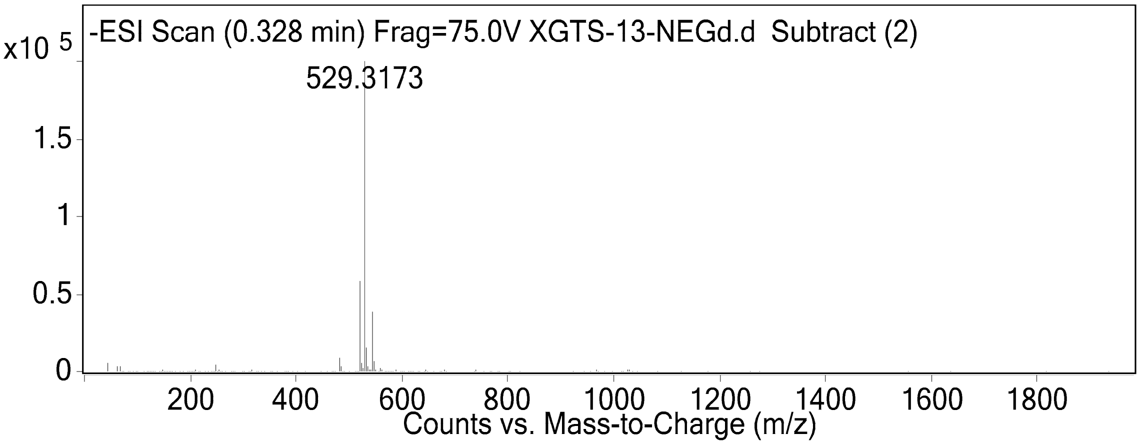


**Figure S56** HR-ESI-MS spectrum of compound **13**

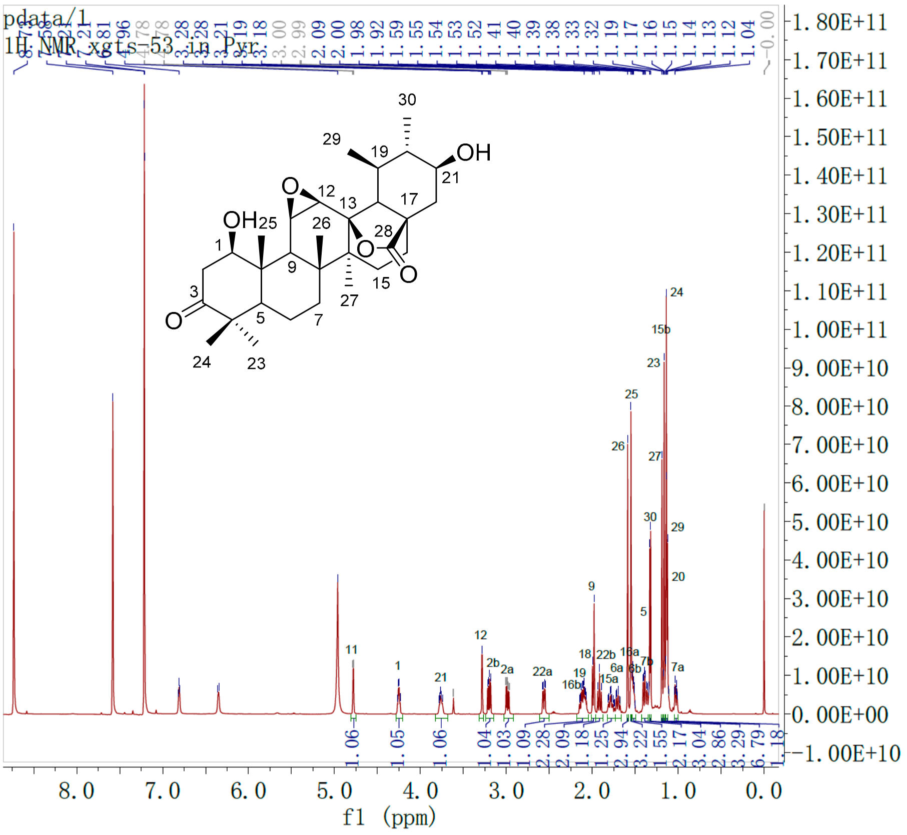


| Parameter | Value |
|---|---|
| 1 Data File Name | E:/ NMR data/ 14/ H/ pdata/ 1/ 1r |
| 2 Title | pdata/ 1 |
| 3 Comment | 1H NMR xgts-53 in Pyr |
| 4 Origin | Bruker BioSpin GmbH |
| 5 Owner | nmrsu |
| 6 Site | |
| 7 Spectrometer | Avance Neo |
| 8 Author | |
| 9 Solvent | Pyr |
| 10 Temperature | 298.0 |
| 11 Pulse Sequence | zg30 |
| 12 Experiment | 1D |
| 13 Probe | Z167963_0004 (CP2.1 DCH 600S3 C/ H-D-05 Z XT) |
| 14 Number of Scans | 8 |
| 15 Receiver Gain | 52 |
| 16 Relaxation Delay | 1.0000 |
| 17 Pulse Width | 10.0000 |
| 18 Presaturation Frequency | |
| 19 Acquisition Time | 2.7525 |
| 20 Acquisition Date | 2022-09-13T10:41:37 |
| 21 Modification Date | 2022-09-13T10:40:34 |
| 22 Class | |
| 23 Spectrometer Frequency | 600.13 |
| 24 Spectral Width | 11904.8 |
| 25 Lowest Frequency | -2226.0 |
| 26 Nucleus | 1H |
| 27 Acquired Size | 32768 |
| 28 Spectral Size | 65536 |

**Figure S57** $^{1}$H NMR spectrum (600 MHz) of compound **14** in Pyridine-$d_5$

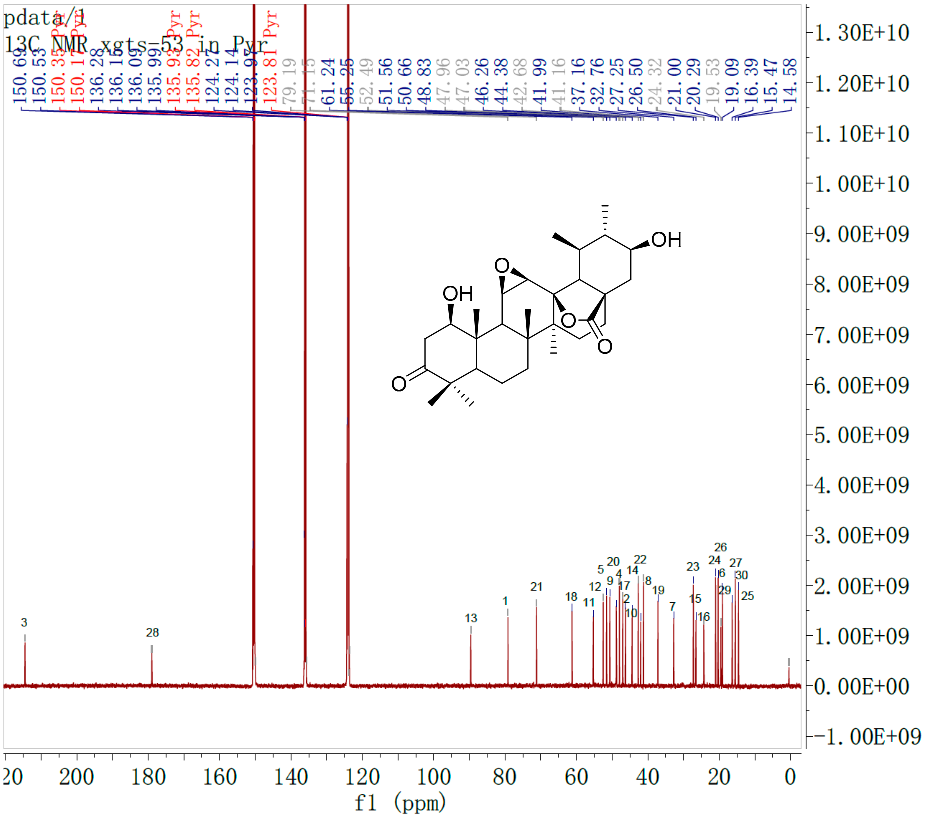


| Parameter | Value |
|---|---|
| 1 Data File Name | E:/ NMR data/ 14/ C/ pdata/ 1/ 1r |
| 2 Title | pdata/ 1 |
| 3 Comment | 13C NMR xgts-53 in Pyr |
| 4 Origin | Bruker BioSpin GmbH |
| 5 Owner | nmrsu |
| 6 Site | |
| 7 Spectrometer | Avance Neo |
| 8 Author | |
| 9 Solvent | Pyr |
| 10 Temperature | 298.0 |
| 11 Pulse Sequence | zgpg30 |
| 12 Experiment | 1D |
| 13 Probe | Z167963_0004 (CP2.1 DCH 600S3 C/ H-D-05 Z XT) |
| 14 Number of Scans | 56 |
| 15 Receiver Gain | 20 |
| 16 Relaxation Delay | 2.0000 |
| 17 Pulse Width | 10.0000 |
| 18 Presaturation Frequency | |
| 19 Acquisition Time | 0.8847 |
| 20 Acquisition Date | 2022-09-15T14:51:49 |
| 21 Modification Date | 2022-09-15T14:50:34 |
| 22 Class | |
| 23 Spectrometer Frequency | 150.90 |
| 24 Spectral Width | 37037.0 |
| 25 Lowest Frequency | -2567.3 |
| 26 Nucleus | 13C |
| 27 Acquired Size | 32768 |
| 28 Spectral Size | 32768 |

**Figure S58** $^{13}$C NMR spectrum (150 MHz) of compound **14** in Pyridine-$d_5$

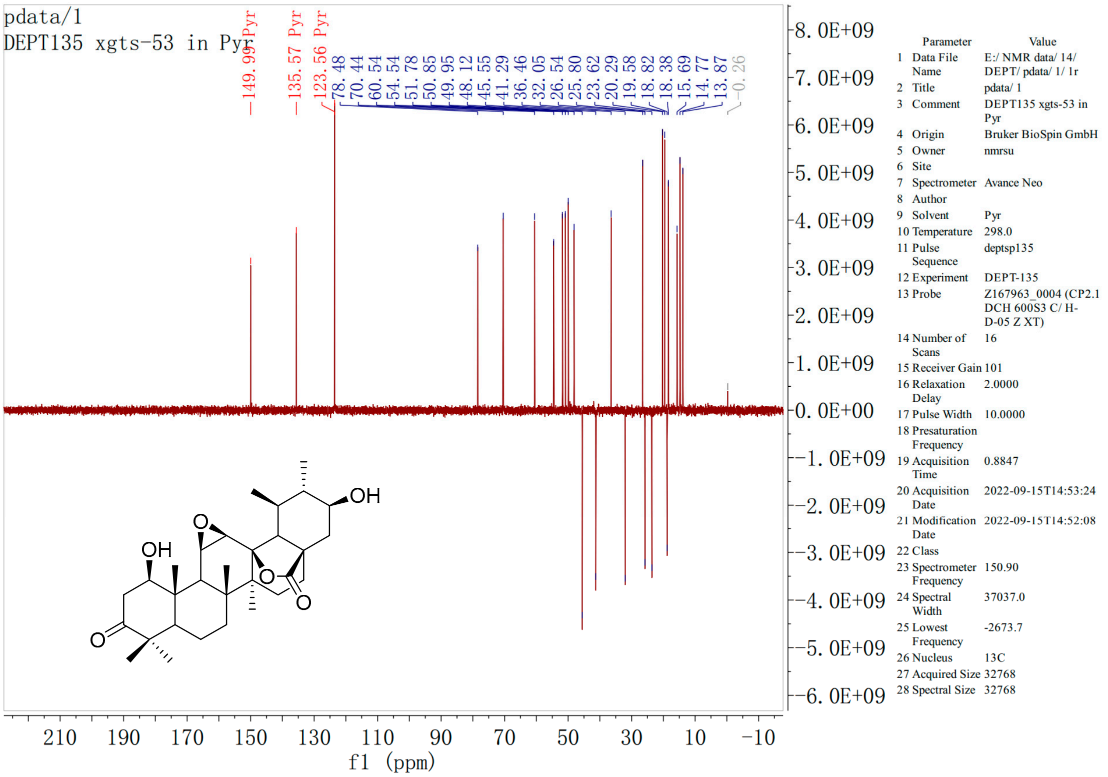


**Figure S59** DEPT-135 spectrum (150 MHz) of compound **14** in Pyridine-$d_5$

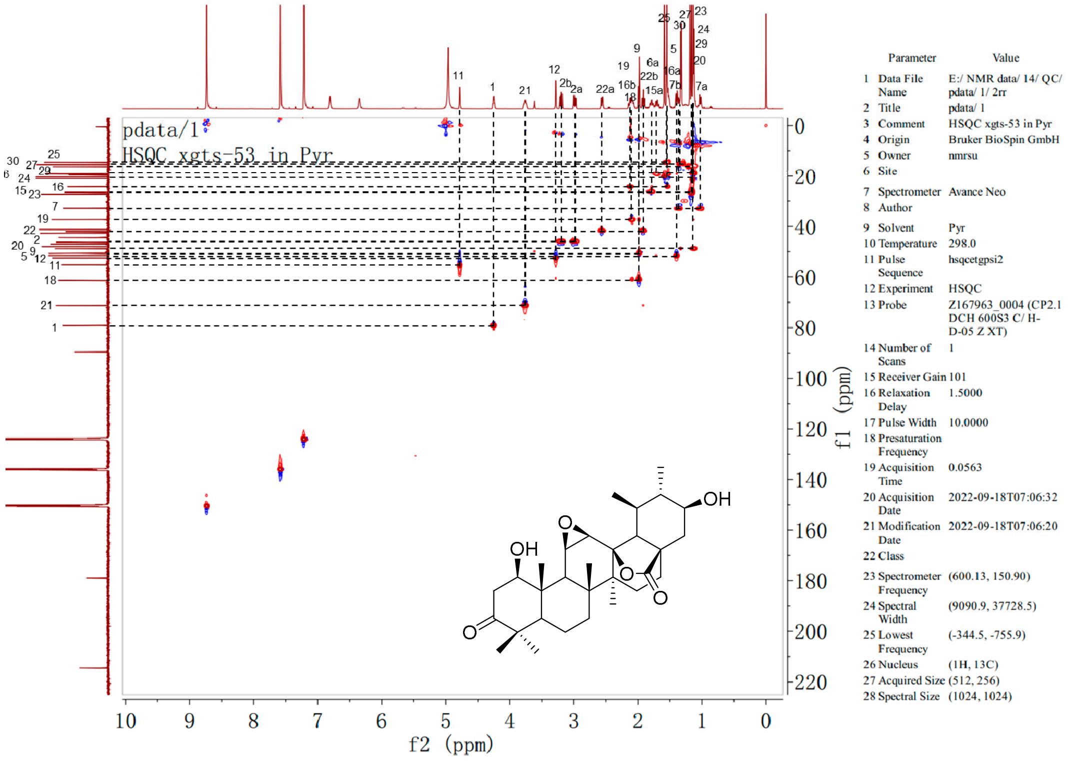


**Figure S60** HSQC spectrum (150 MHz) of compound **14** in Pyridine-$d_5$

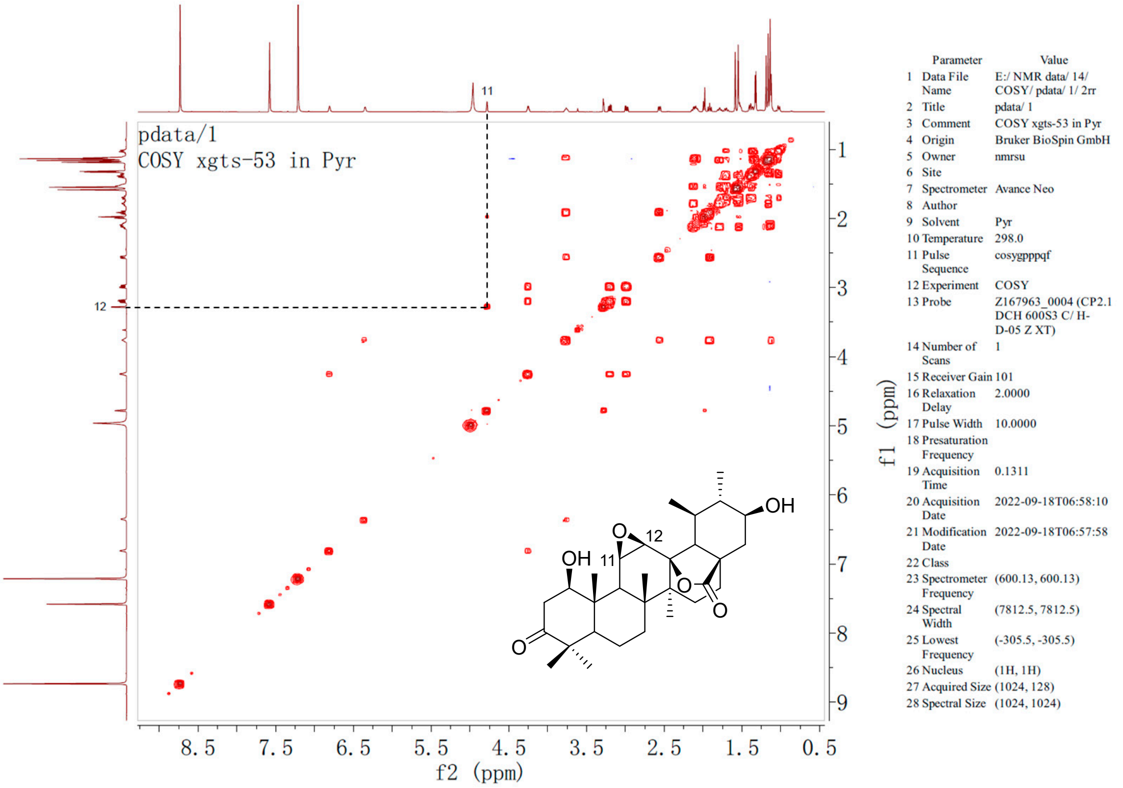


**Figure S61** $^{1}$H-$^{1}$H COSY spectrum (600 MHz) of compound **14** in Pyridine-$d_5$

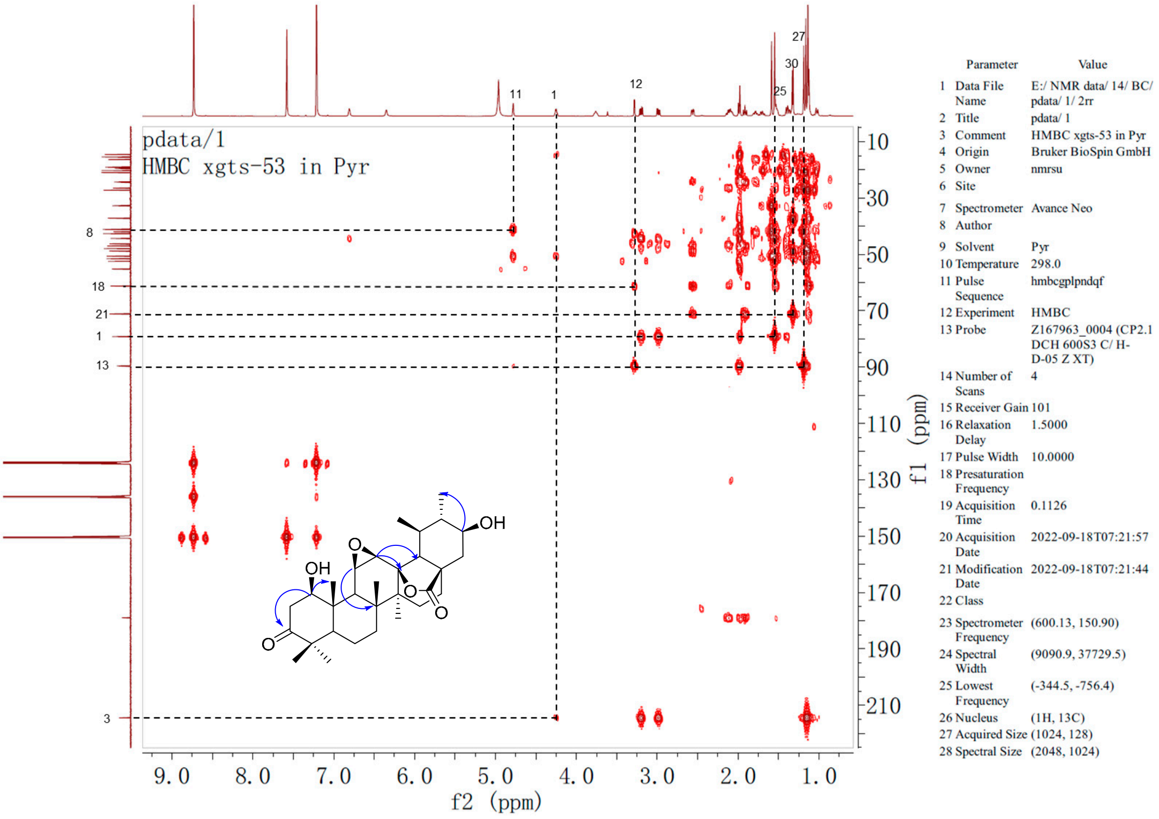


**Figure S62** HMBC spectrum (150 MHz) of compound **14** in Pyridine-$d_5$

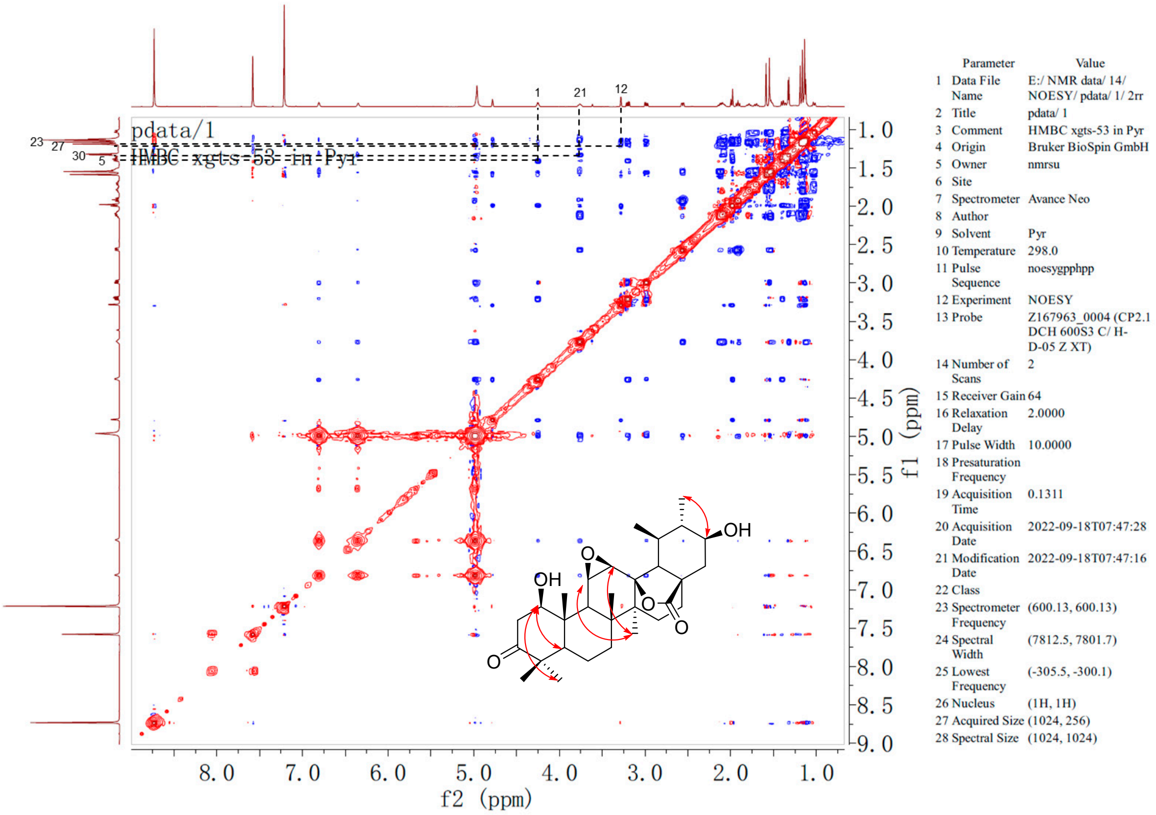


**Figure S63** NOESY spectrum (600 MHz) of compound **14** in Pyridine-$d_5$

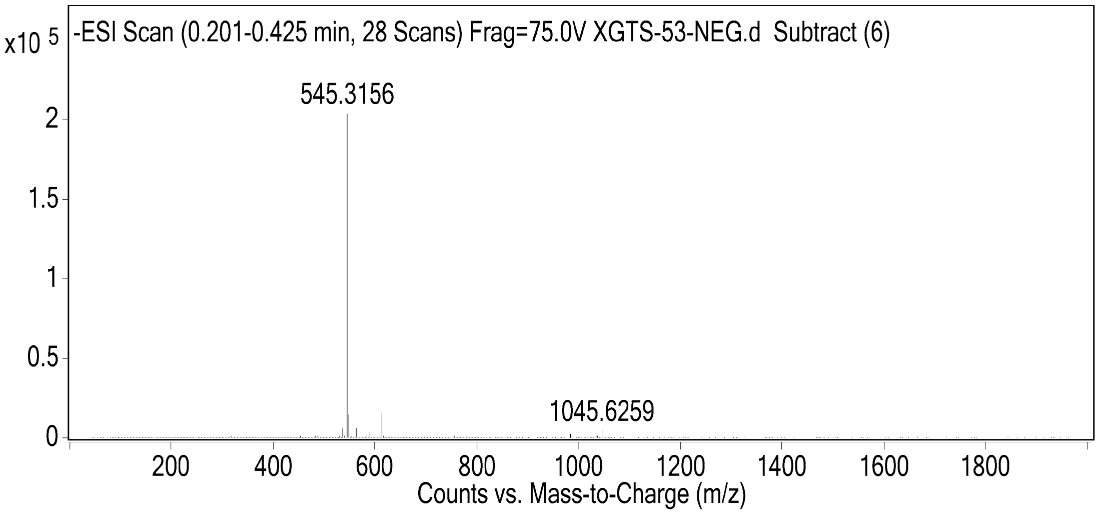


**Figure S64** HR-ESI-MS spectrum of compound **14**

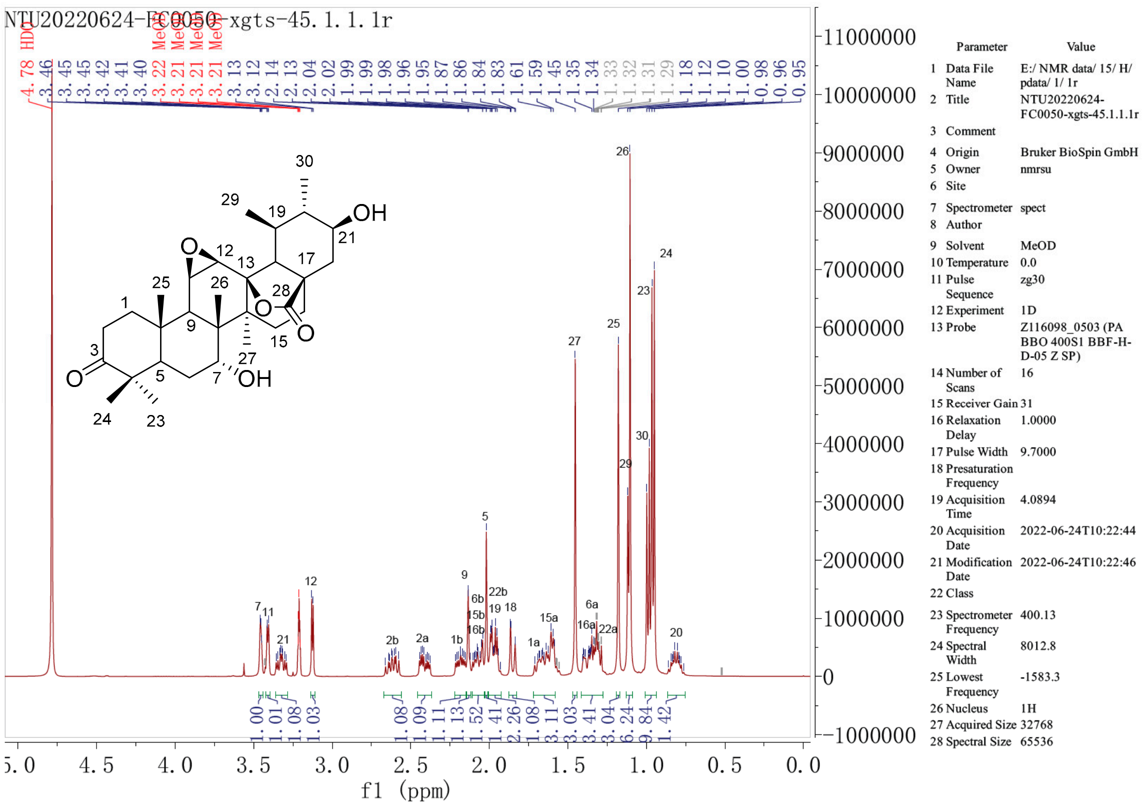

**Figure S65** $^{1}$H NMR spectrum (400 MHz) of compound **15** in $CD_3OD$

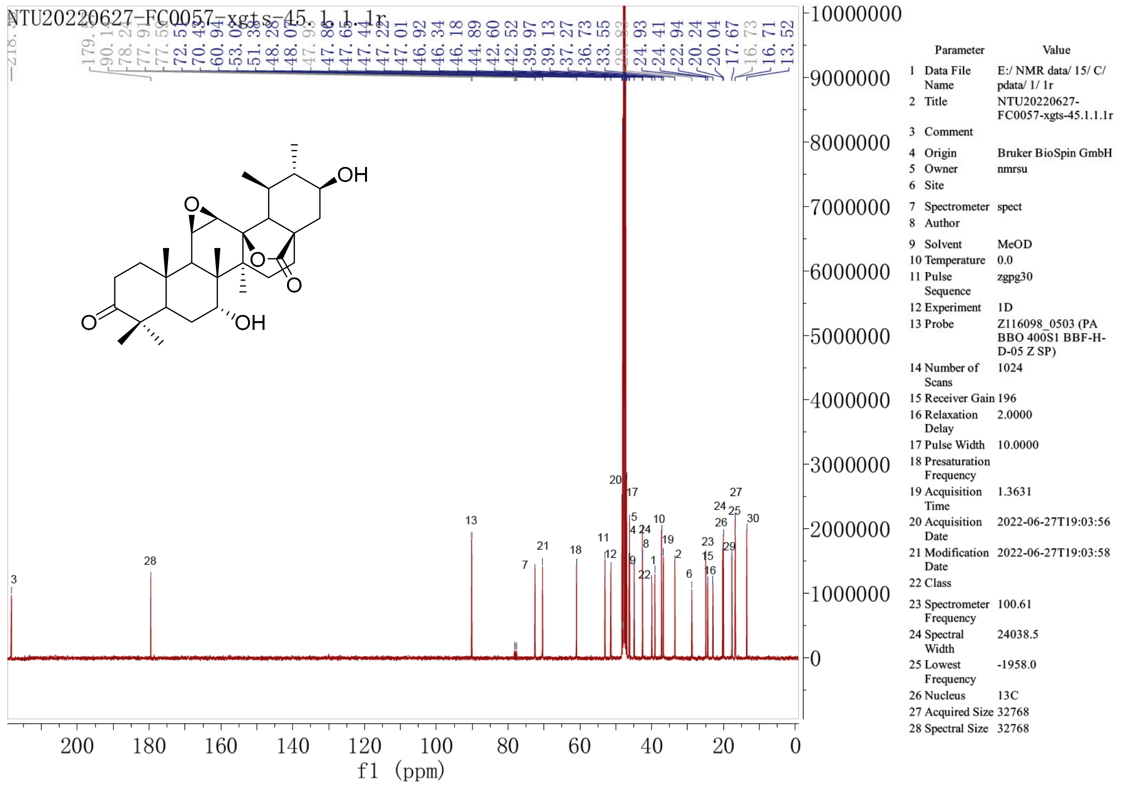

**Figure S66** $^{13}$C NMR spectrum (100 MHz) of compound **15** in $CD_3OD$

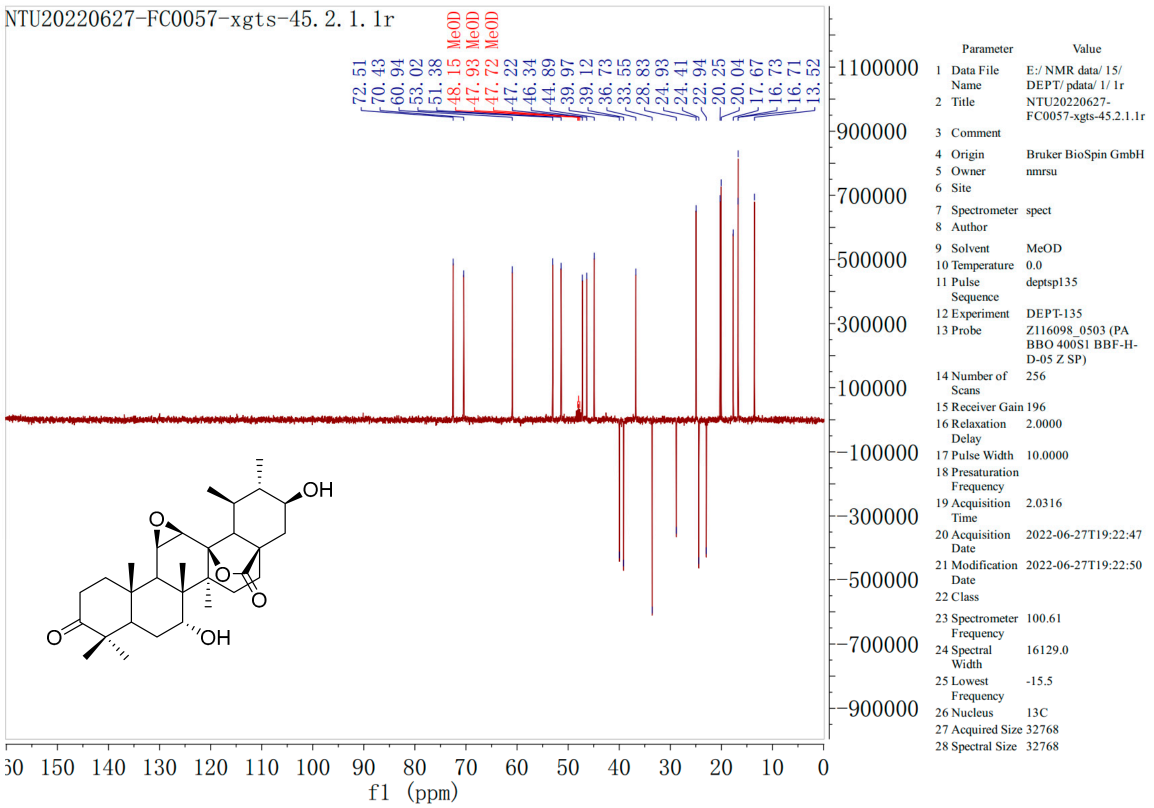


**Figure S67** DEPT-135 spectrum (100 MHz) of compound **15** in $CD_3OD$

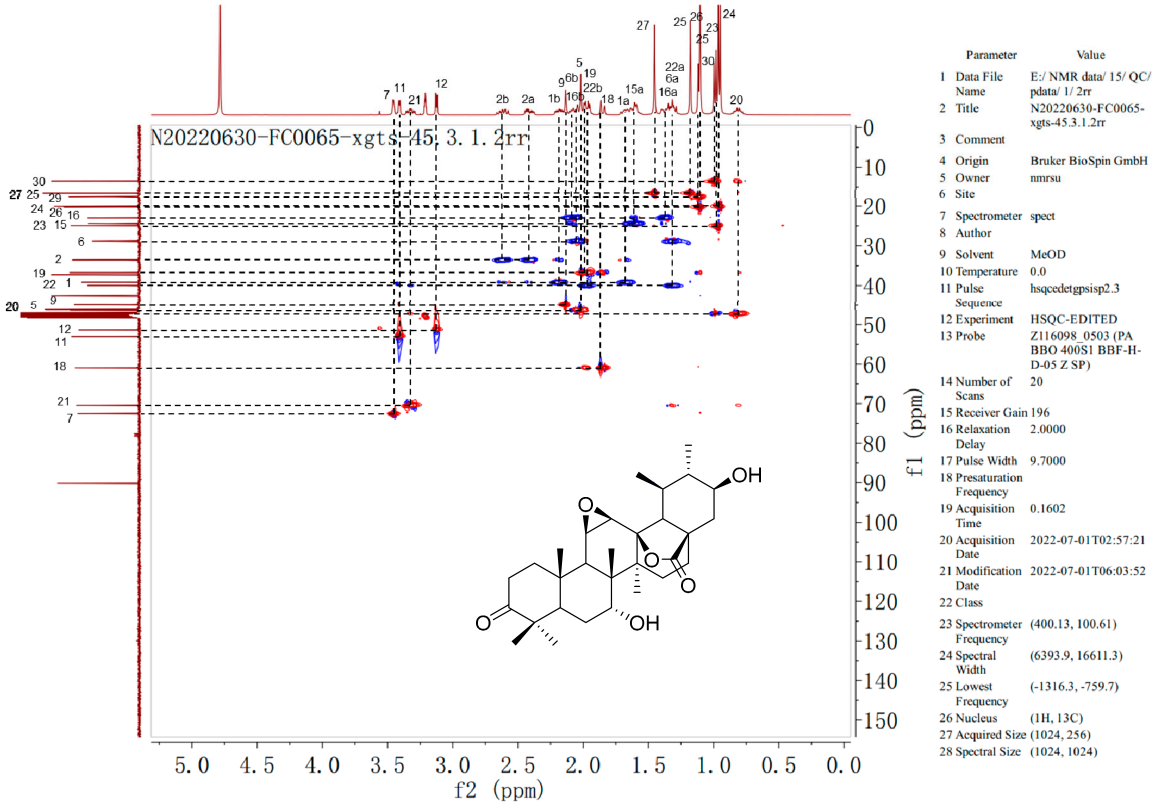


**Figure S68** HSQC spectrum (100 MHz) of compound **15** in $CD_3OD$

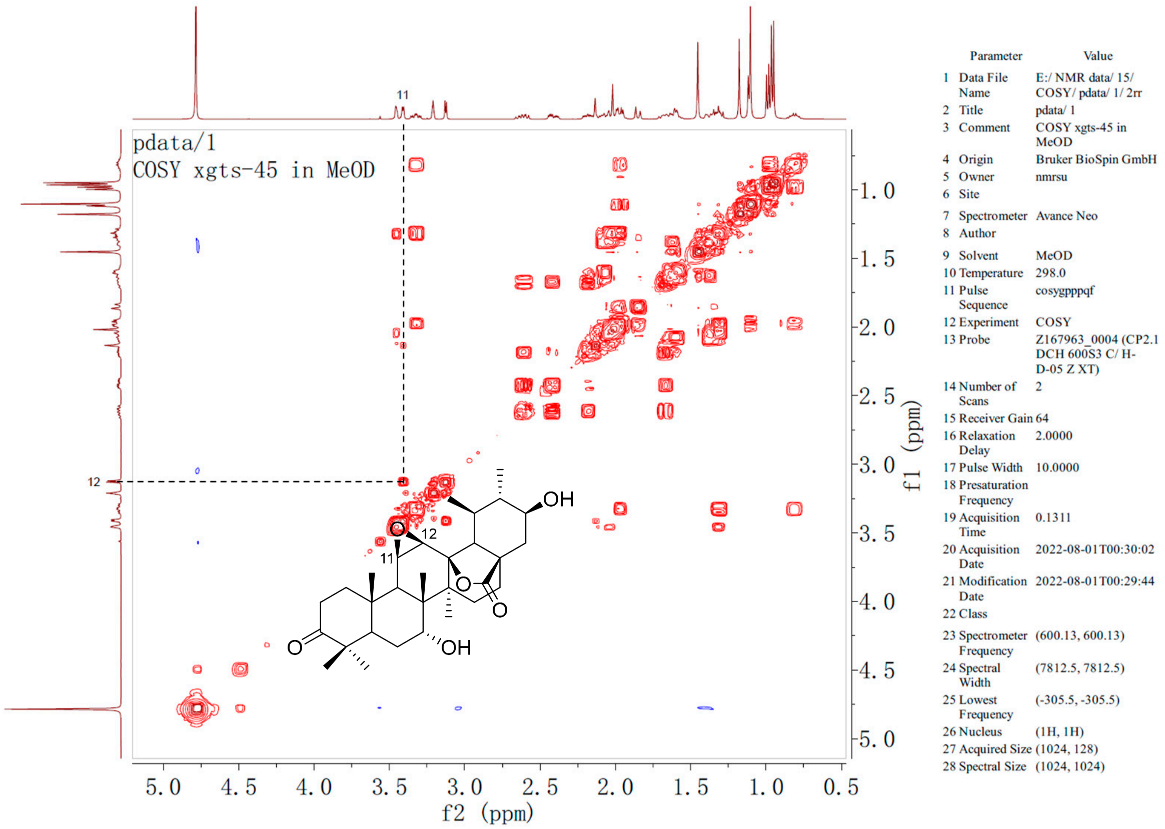


**Figure S69** $^{1}$H-$^{1}$H COSY spectrum (400 MHz) of compound **15** in $CD_3OD$

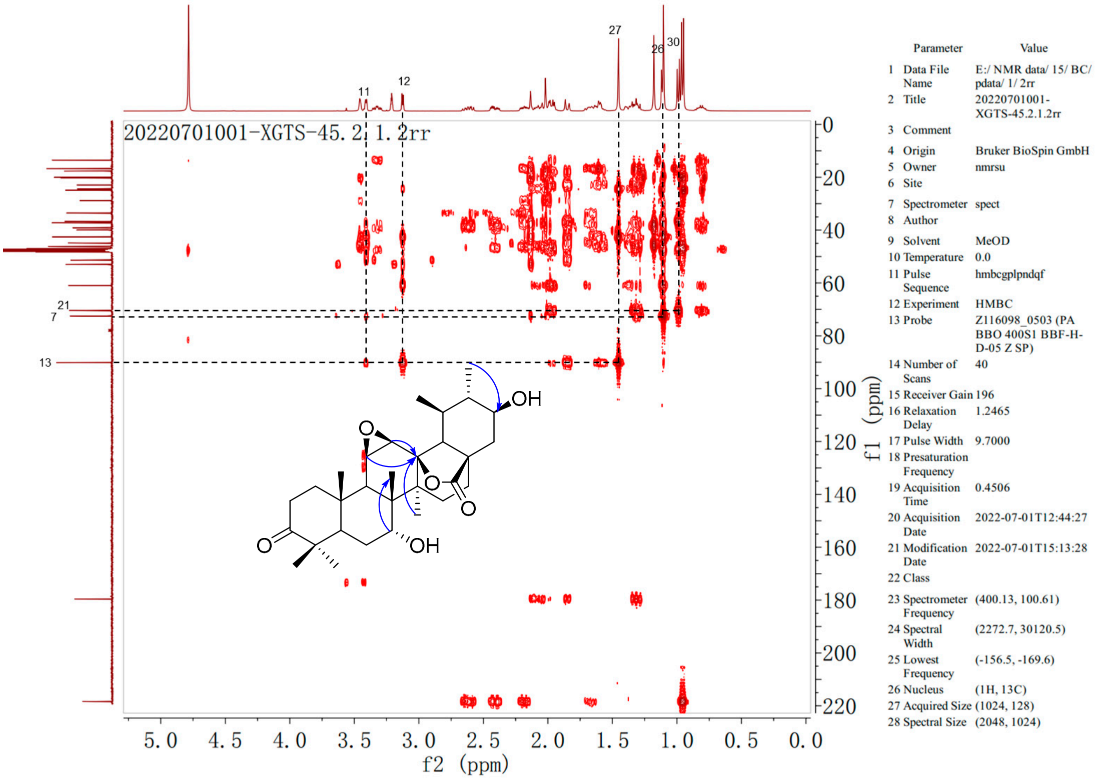


**Figure S70** HMBC spectrum (100 MHz) of compound **15** in $CD_3OD$

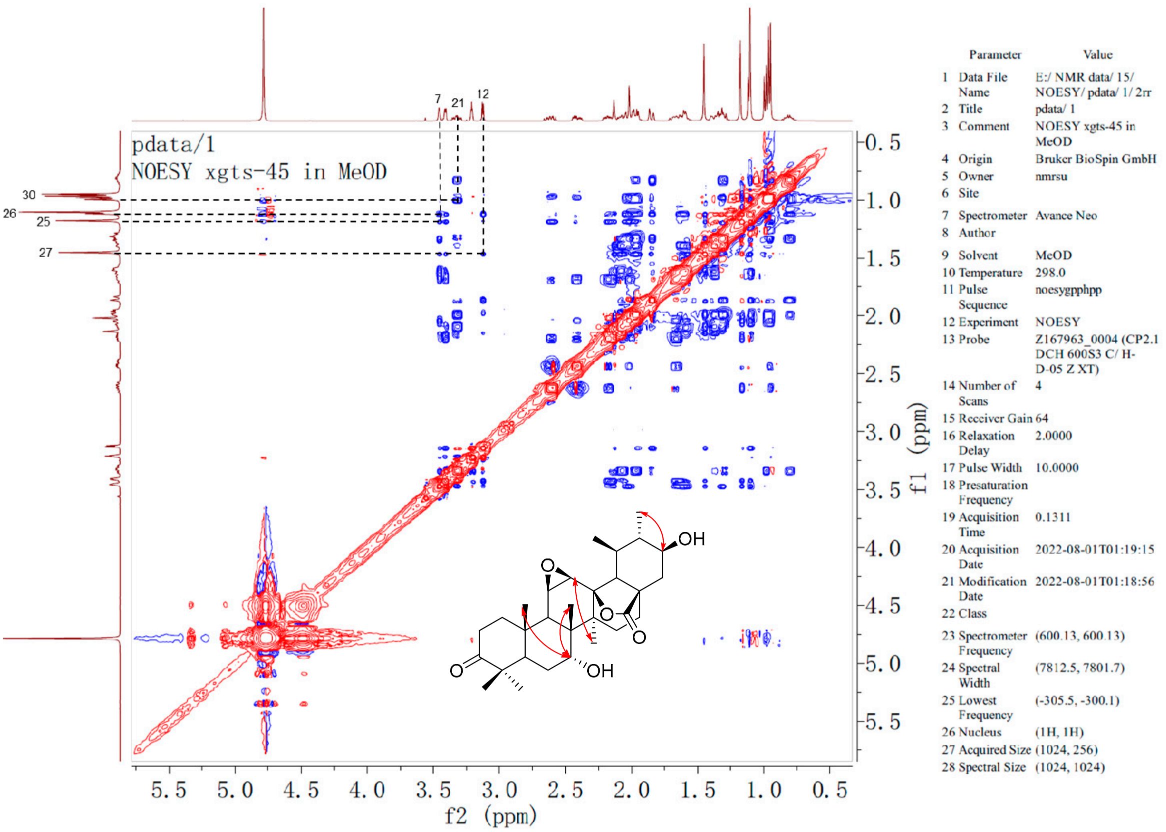


**Figure S71** NOESY spectrum (400 MHz) of compound **15** in $CD_3OD$

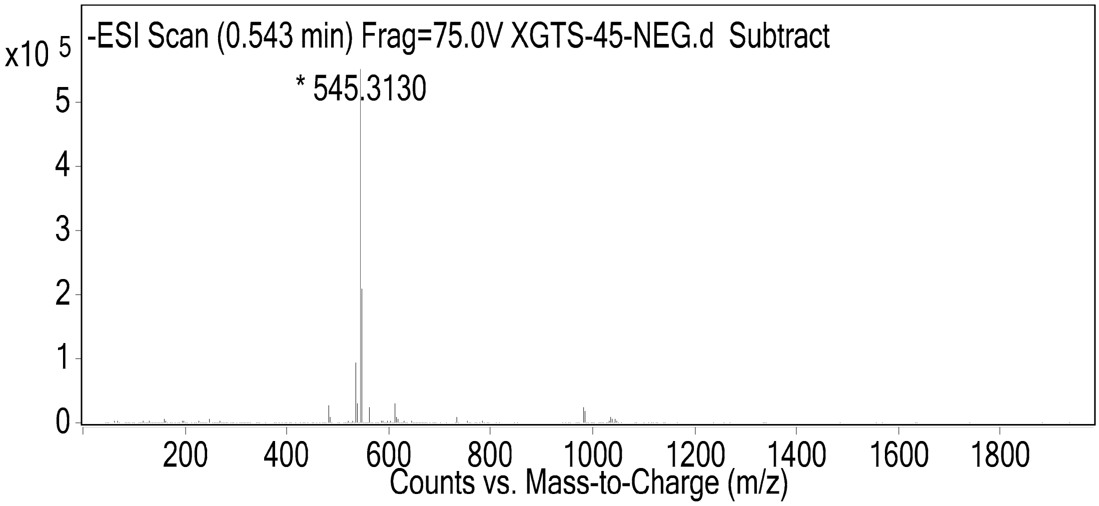


**Figure S72** HR-ESI-MS spectrum of compound **15**

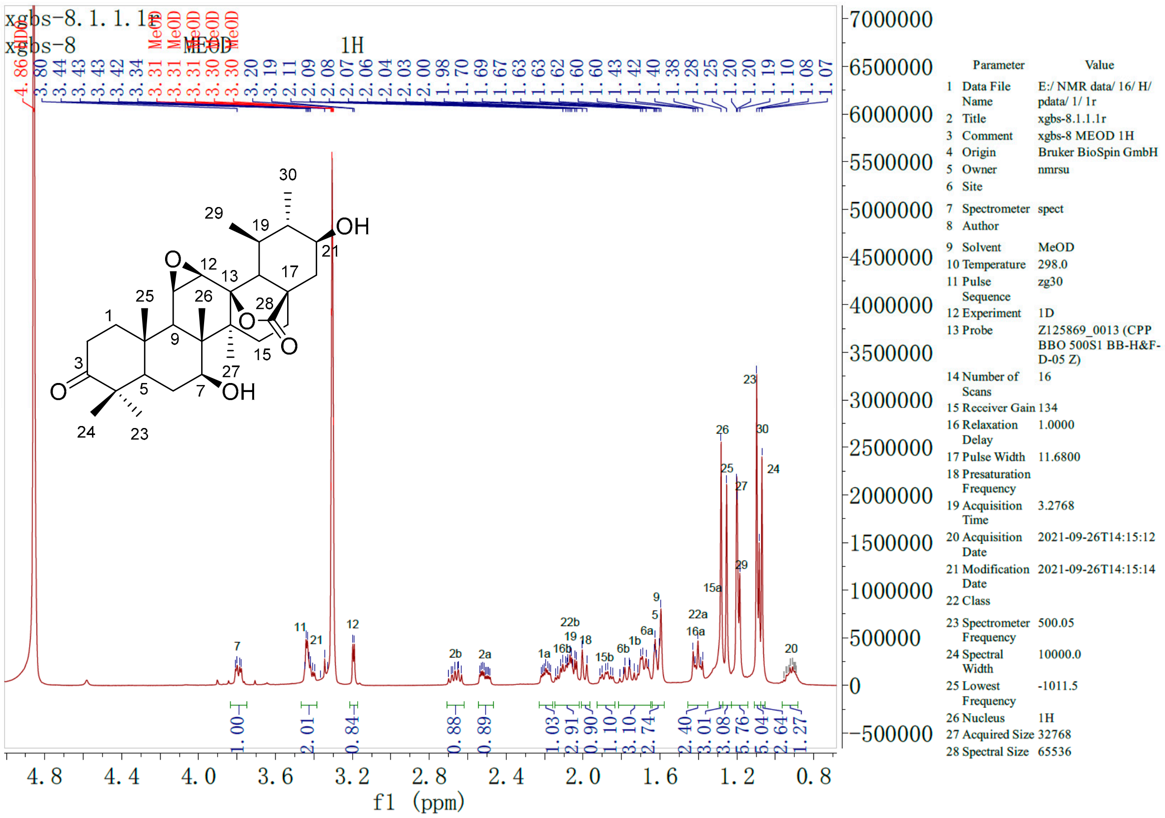


**Figure S73** $^{1}$H NMR spectrum (500 MHz) of compound **16** in $CD_3OD$

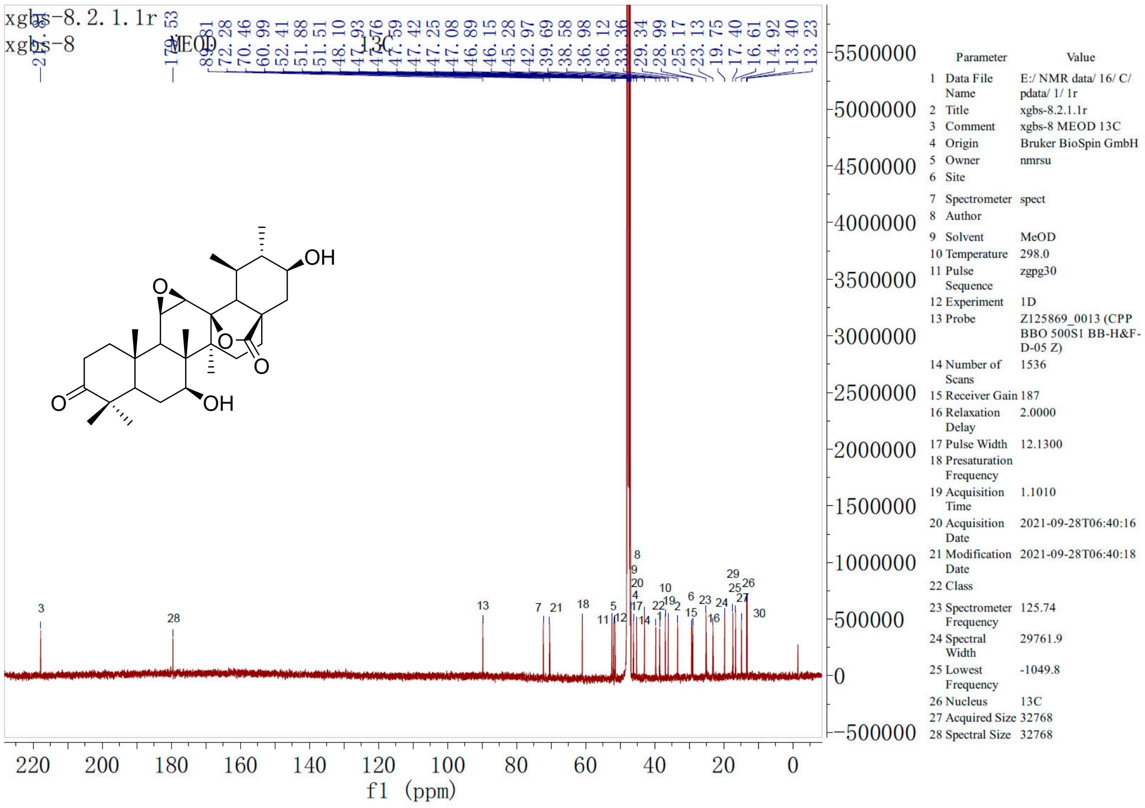


**Figure S74** $^{13}$C NMR spectrum (125 MHz) of compound **16** in $CD_3OD$

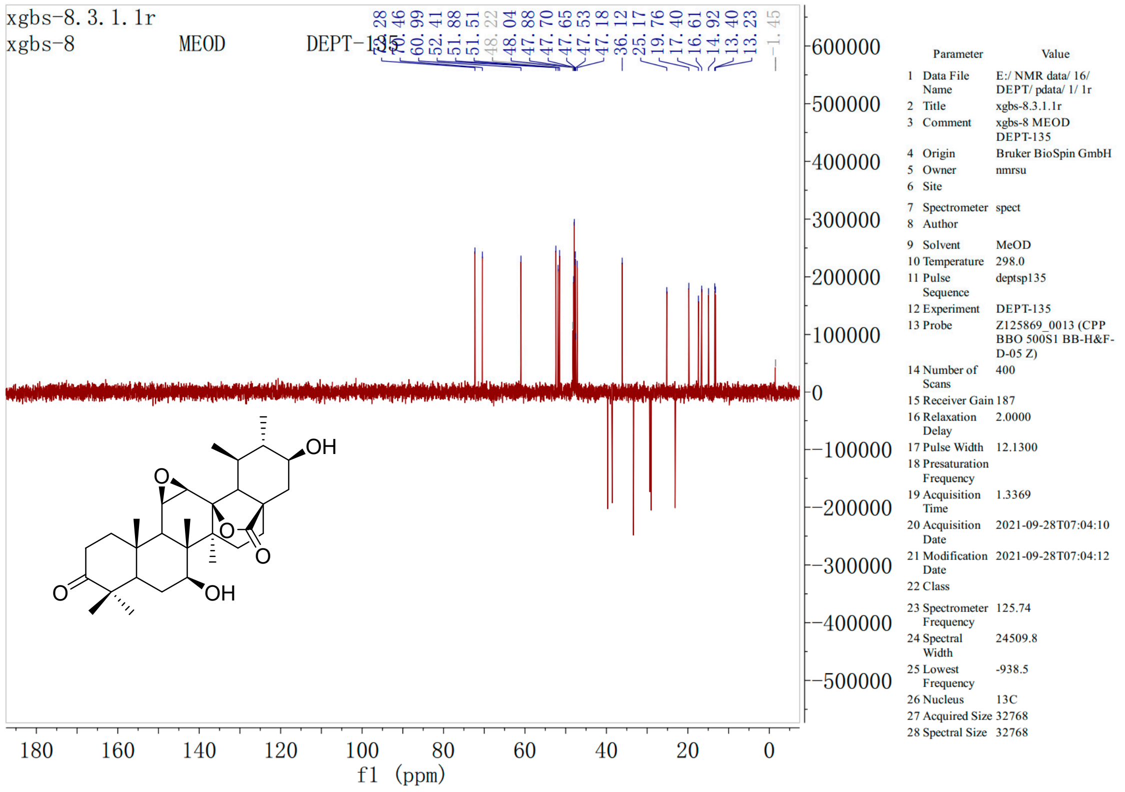


**Figure S75** DEPT-135 spectrum (125 MHz) of compound **16** in $CD_3OD$

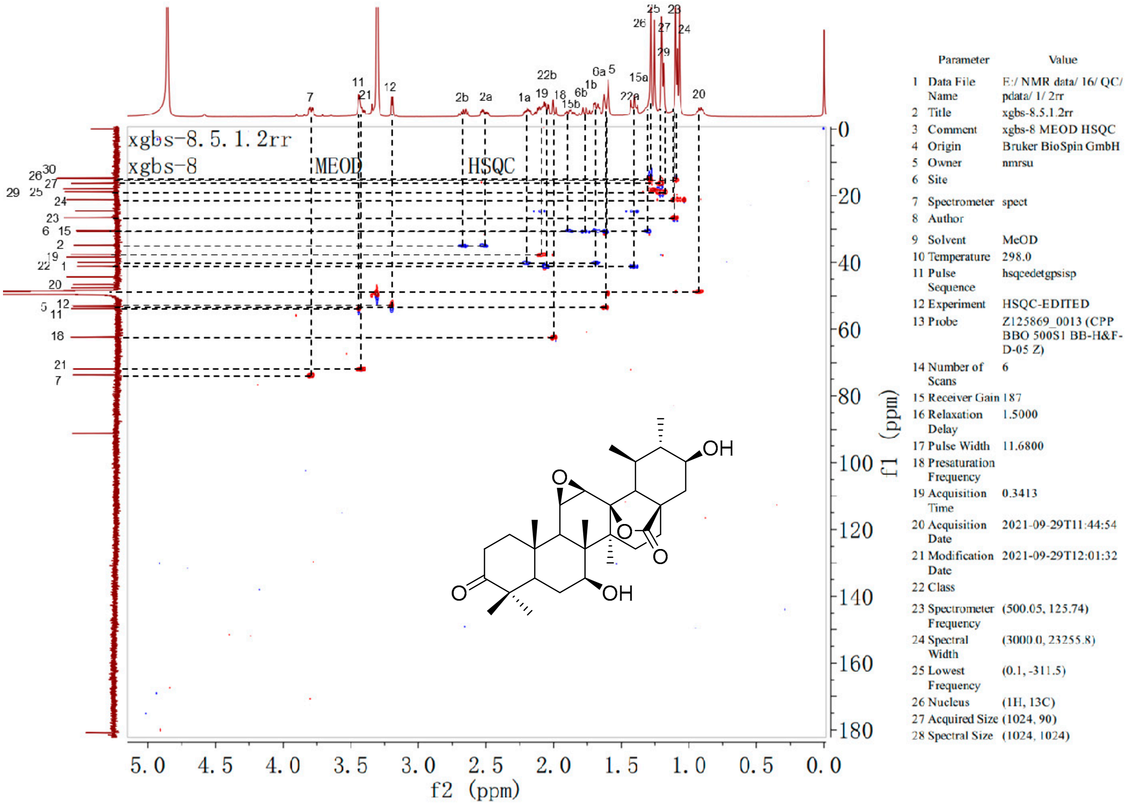


**Figure S76** HSQC spectrum (125 Mz) of compound **16** in $CD_3OD$

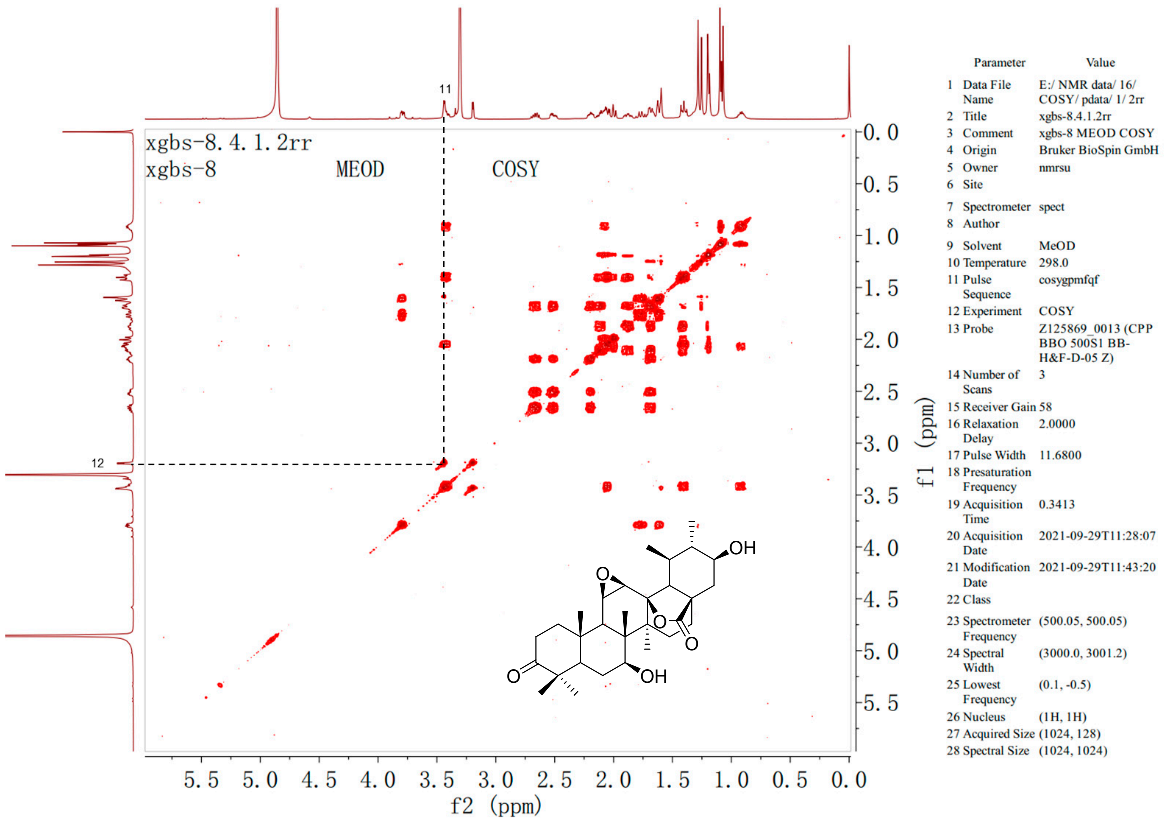


**Figure S77** $^{1}H$-$^{1}H$ COSY spectrum (400 MHz) of compound **16** in $CD_3OD$

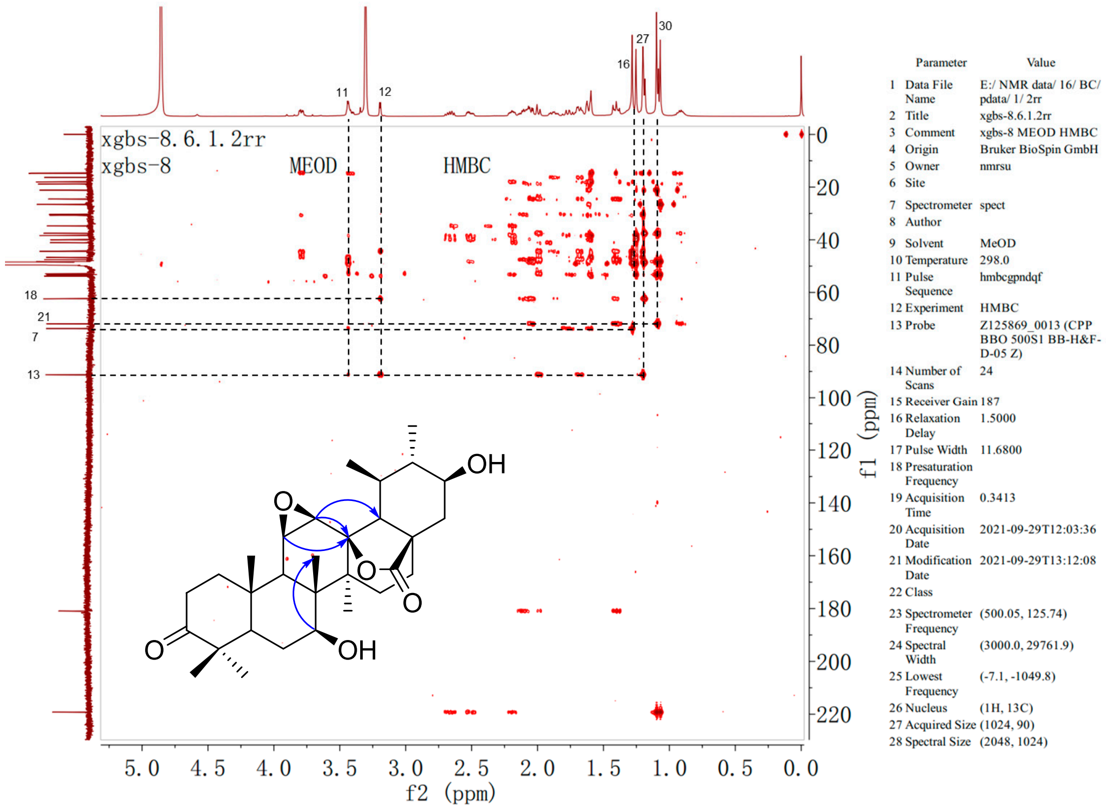


**Figure S78** HMBC spectrum (125 MHz) of compound **16** in $CD_3OD$

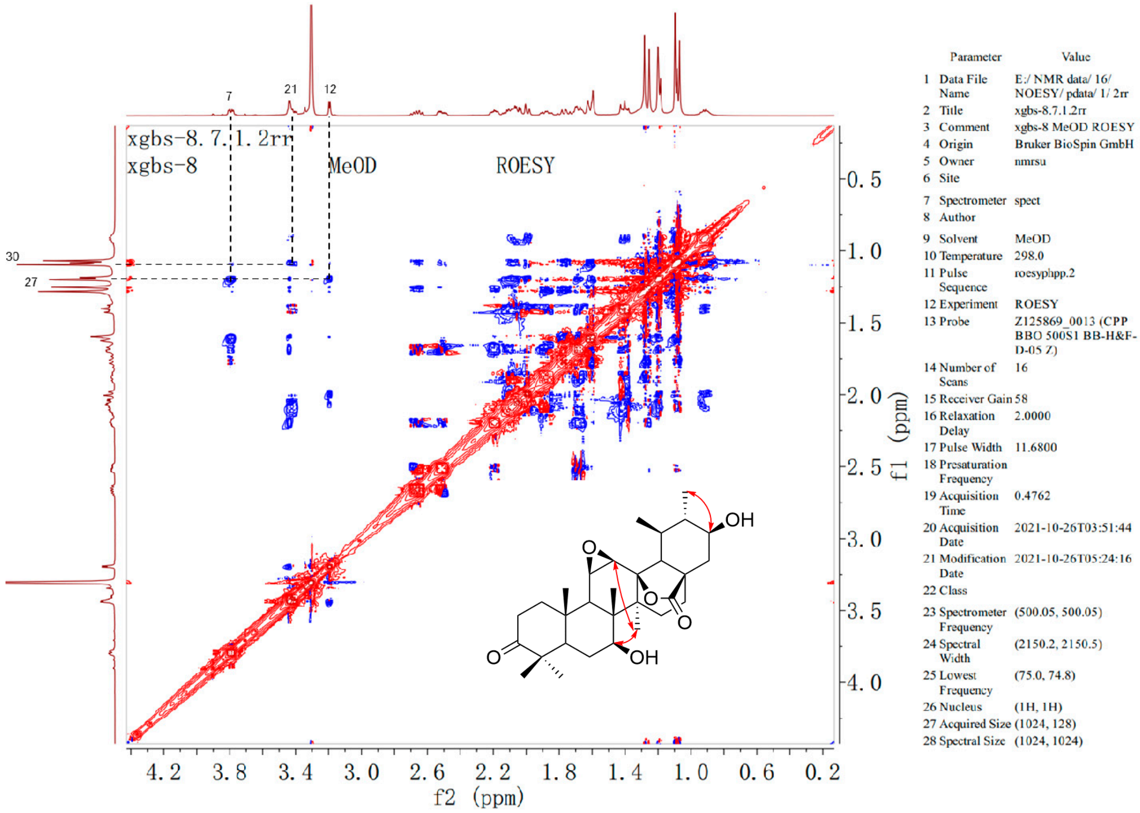


**Figure S79** NOESY spectrum (500 MHz) of compound **16** in $CD_3OD$

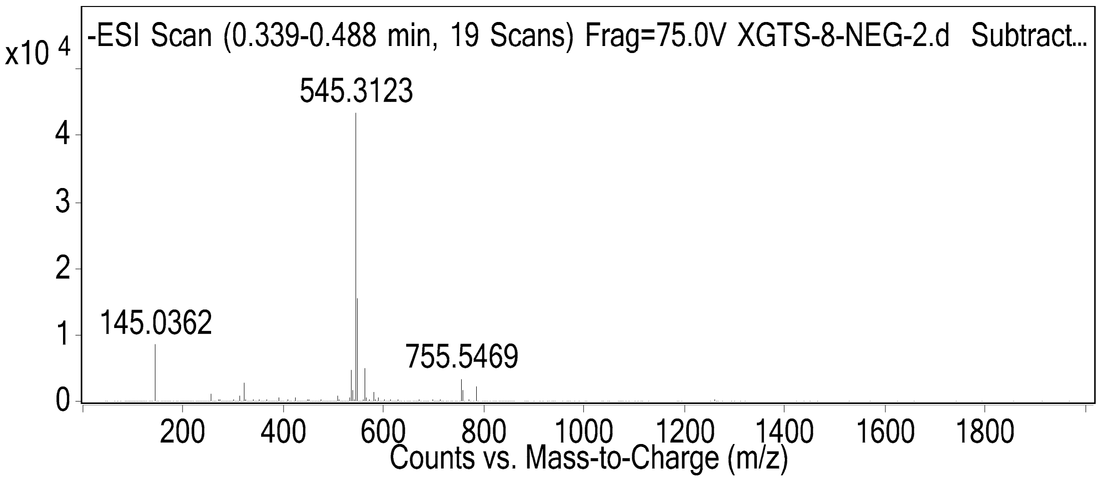


**Figure S80** HR-ESI-MS spectrum of compound **16**

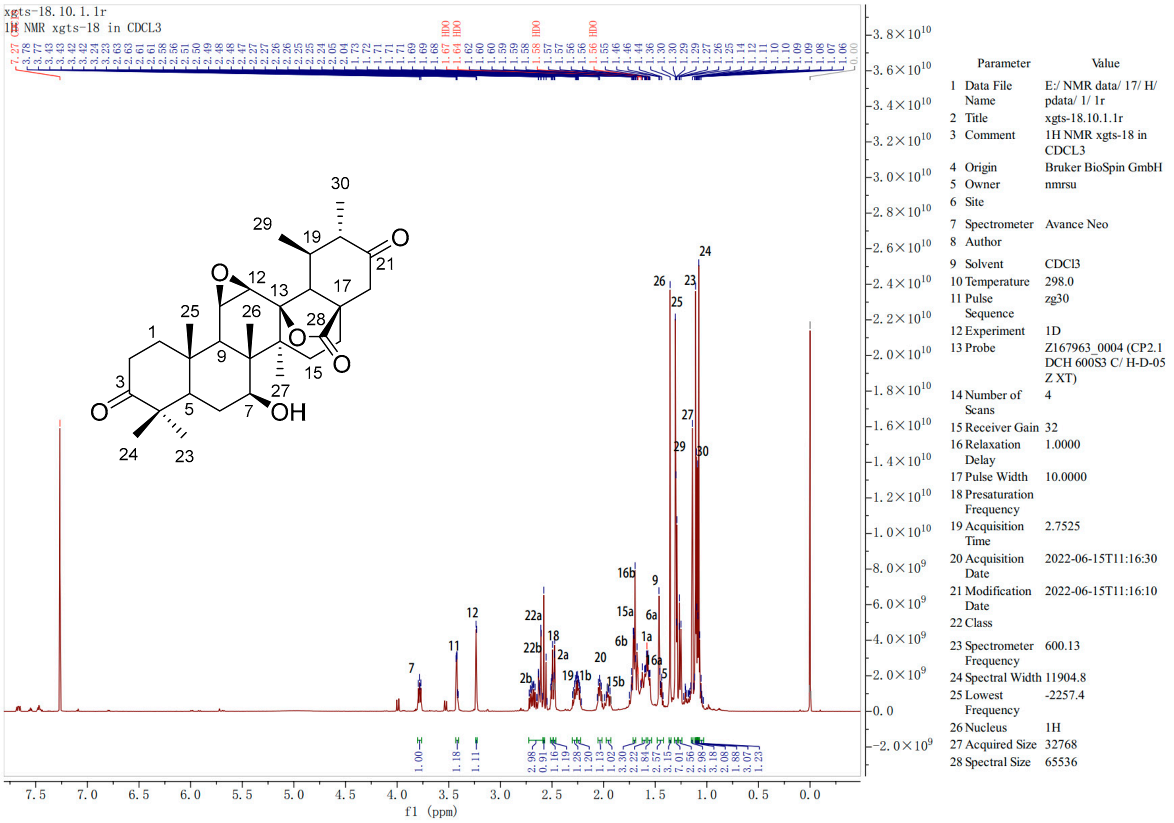

**Figure S81** $^{1}$H NMR spectrum (400 MHz) of compound **17** in $CDCl_3$

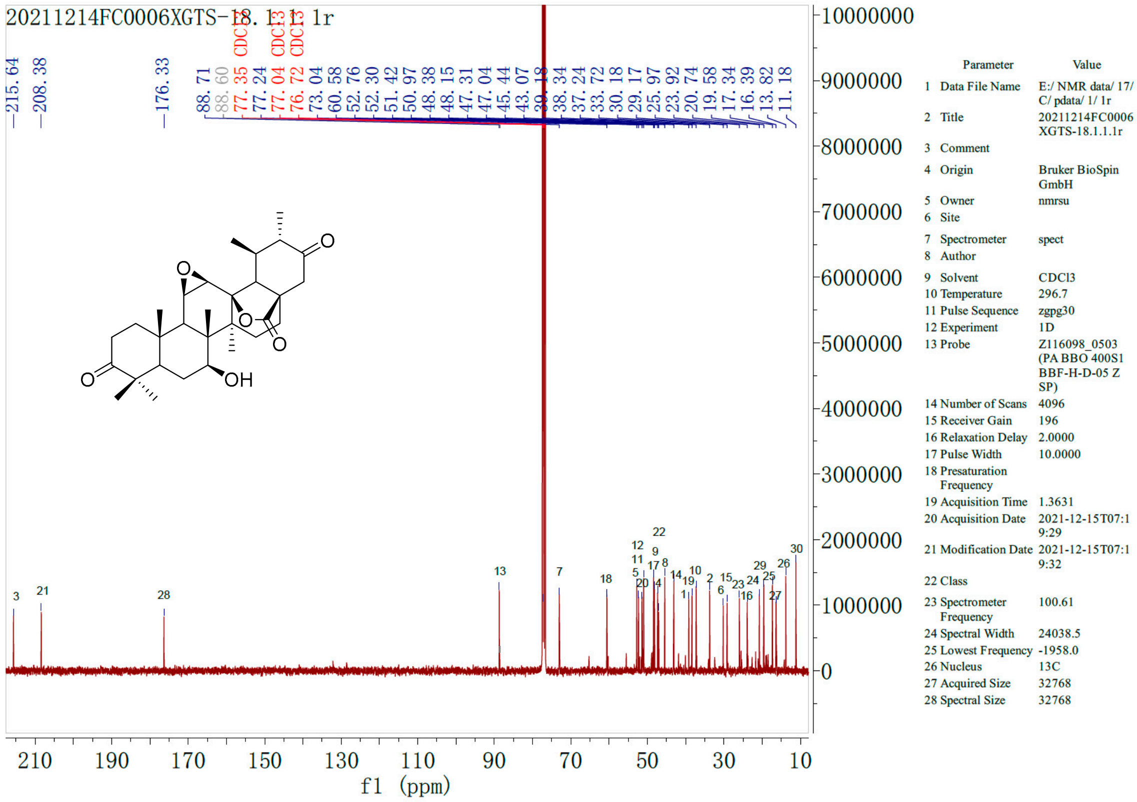

**Figure S82** $^{13}$C NMR spectrum (100 MHz) of compound **17** in $CDCl_3$

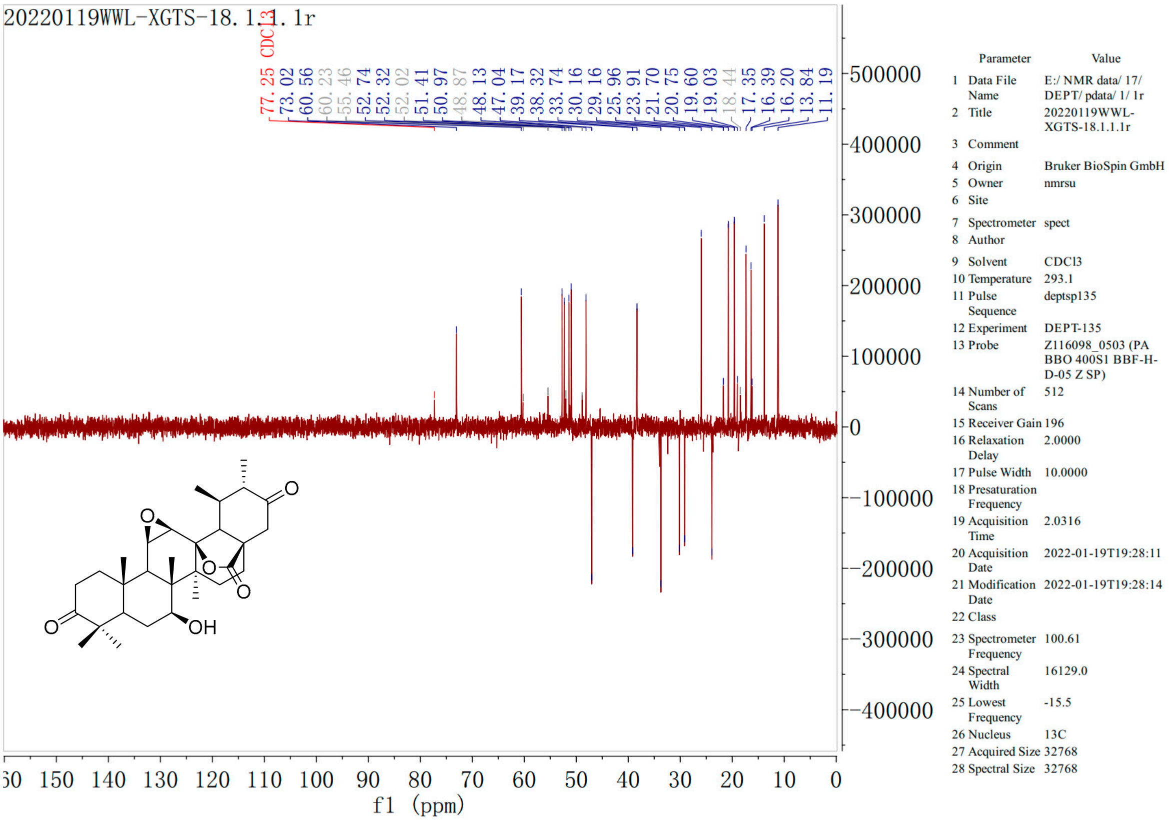


**Figure S83** DEPT-135 spectrum (100 MHz) of compound **17** in $CDCl_3$

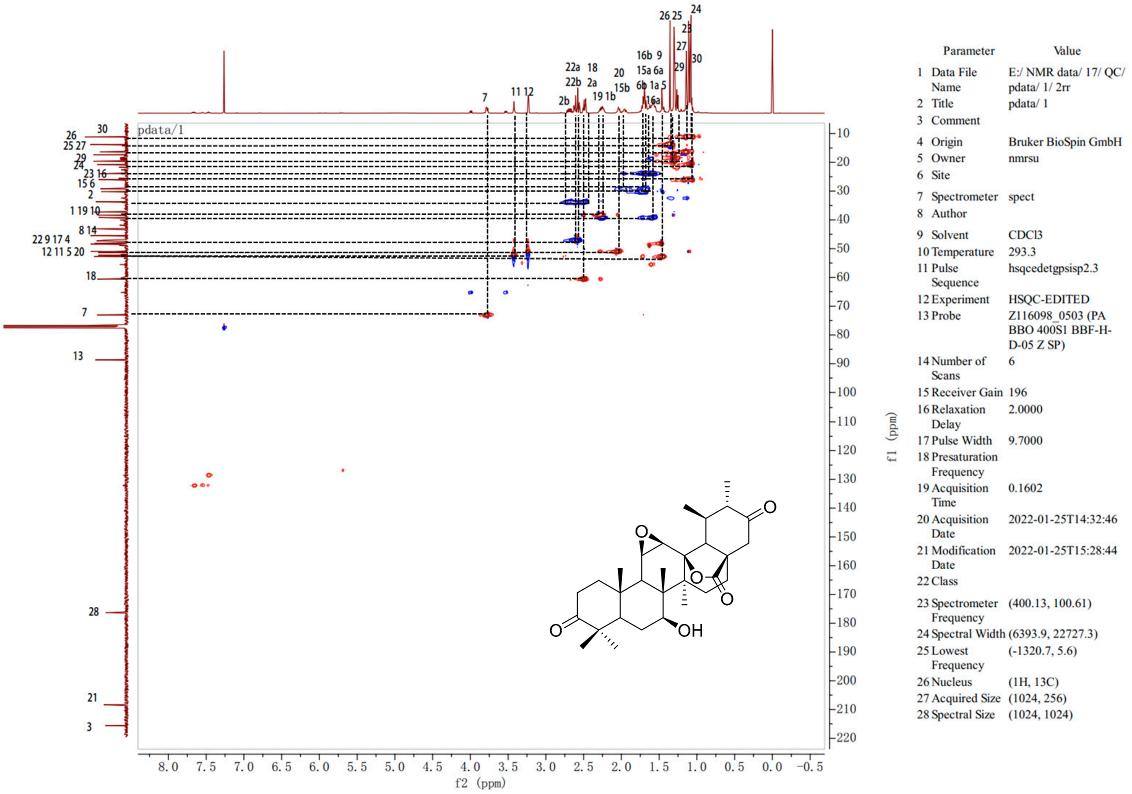


**Figure S84** HSQC spectrum (100 MHz) of compound **17** in $CDCl_3$

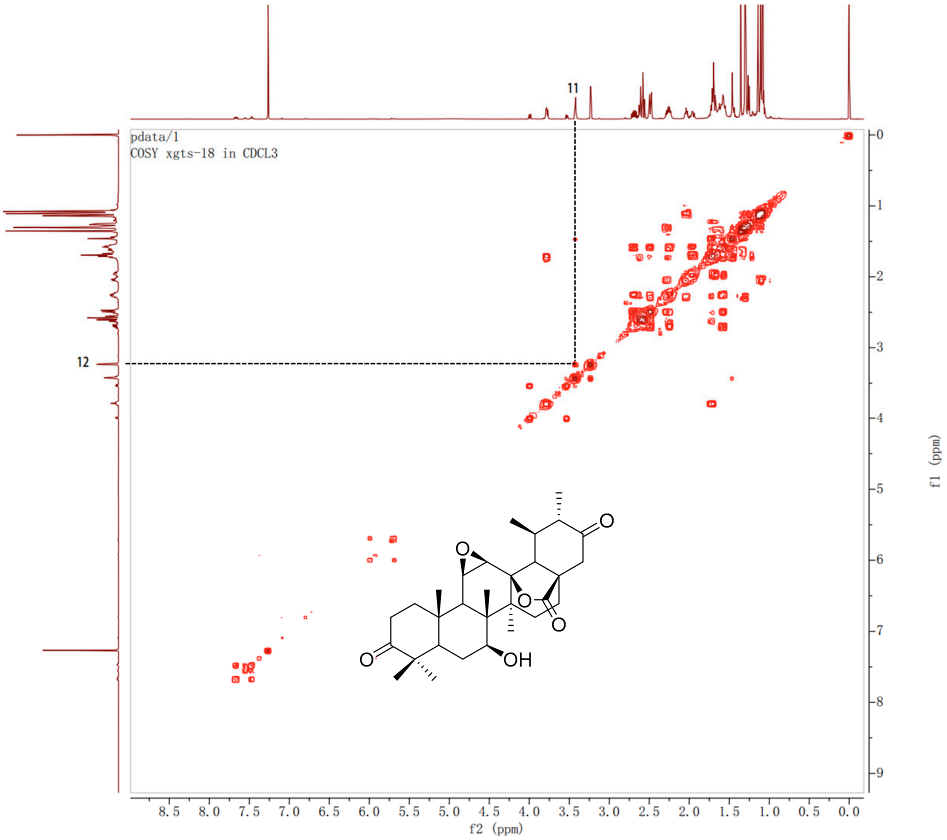


| Parameter | Value |
|---|---|
| 1 Data File Name | E:/ NMR data/ 17/ COSY/ pdata/ 1/ 2rr |
| 2 Title | pdata/ 1 |
| 3 Comment | COSY xgts-18 in CDCL3 |
| 4 Origin | Bruker BioSpin GmbH |
| 5 Owner | nmrsu |
| 6 Site | |
| 7 Spectrometer | Avance Neo |
| 8 Author | |
| 9 Solvent | CDCl3 |
| 10 Temperature | 298.0 |
| 11 Pulse Sequence | cosygpppqf |
| 12 Experiment | COSY |
| 13 Probe | Z167963_0004 (CP2.1 DCH 600S3 C/ H-D-05 Z XT) |
| 14 Number of Scans | 1 |
| 15 Receiver Gain | 101 |
| 16 Relaxation Delay | 2.0000 |
| 17 Pulse Width | 10.0000 |
| 18 Presaturation Frequency | |
| 19 Acquisition Time | 0.1311 |
| 20 Acquisition Date | 2022-06-24T18:35:05 |
| 21 Modification Date | 2022-06-24T18:34:28 |
| 22 Class | |
| 23 Spectrometer Frequency | (600.13, 600.13) |
| 24 Spectral Width | (7812.5, 7812.5) |
| 25 Lowest Frequency | (-313.3, -309.7) |
| 26 Nucleus | (1H, 1H) |
| 27 Acquired Size | (1024, 128) |
| 28 Spectral Size | (1024, 1024) |

**Figure S85** $^{1}$H-$^{1}$H COSY spectrum (400 MHz) of compound **17** in $CDCl_3$

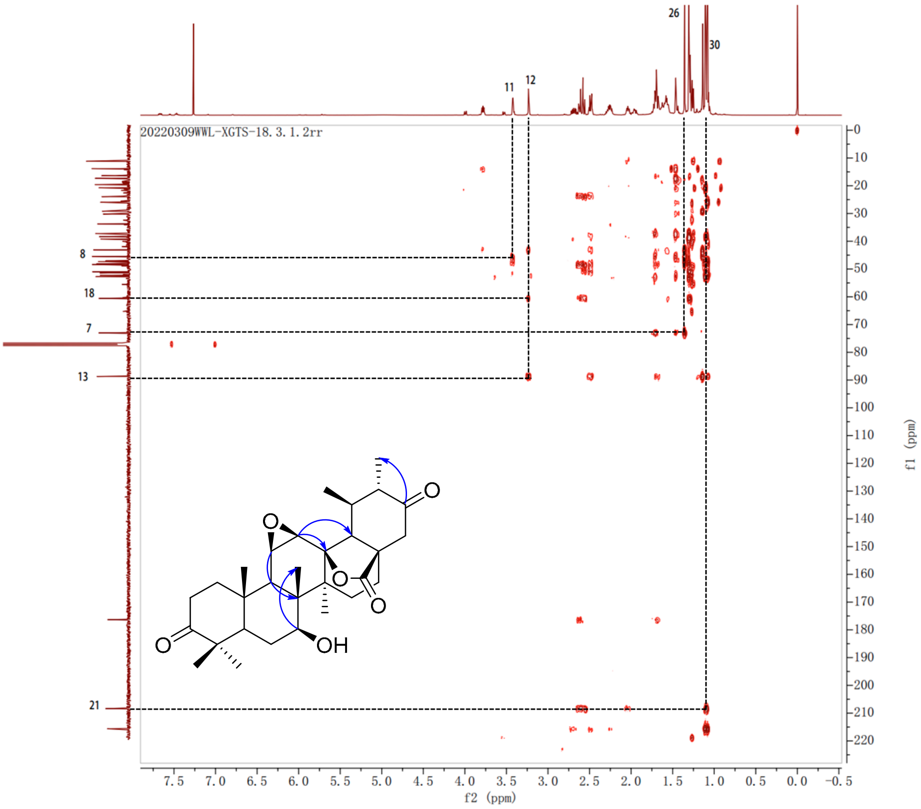


| Parameter | Value |
|---|---|
| 1 Data File Name | E:/ NMR data/ 17/ BC/ pdata/ 1/ 2rr |
| 2 Title | 20220309WWL-XGTS-18.3.1.2rr |
| 3 Comment | |
| 4 Origin | Bruker BioSpin GmbH |
| 5 Owner | nmrsu |
| 6 Site | |
| 7 Spectrometer | spect |
| 8 Author | |
| 9 Solvent | CDCl3 |
| 10 Temperature | 293.8 |
| 11 Pulse Sequence | hmbcgplpndqf |
| 12 Experiment | HMBC |
| 13 Probe | Z116098_0503 (PA BBO 400S1 BBF-H-D-05 Z SP) |
| 14 Number of Scans | 4 |
| 15 Receiver Gain | 196 |
| 16 Relaxation Delay | 1.3939 |
| 17 Pulse Width | 9.7000 |
| 18 Presaturation Frequency | |
| 19 Acquisition Time | 0.3031 |
| 20 Acquisition Date | 2022-03-10T09:48:28 |
| 21 Modification Date | 2022-03-10T10:03:26 |
| 22 Class | |
| 23 Spectrometer Frequency | (400.13, 100.61) |
| 24 Spectral Width | (3378.4, 23148.1) |
| 25 Lowest Frequency | (-215.8, -204.8) |
| 26 Nucleus | (1H, 13C) |
| 27 Acquired Size | (1024, 128) |
| 28 Spectral Size | (2048, 1024) |

**Figure S86** HMBC spectrum (100 MHz) of compound **17** in $CDCl_3$

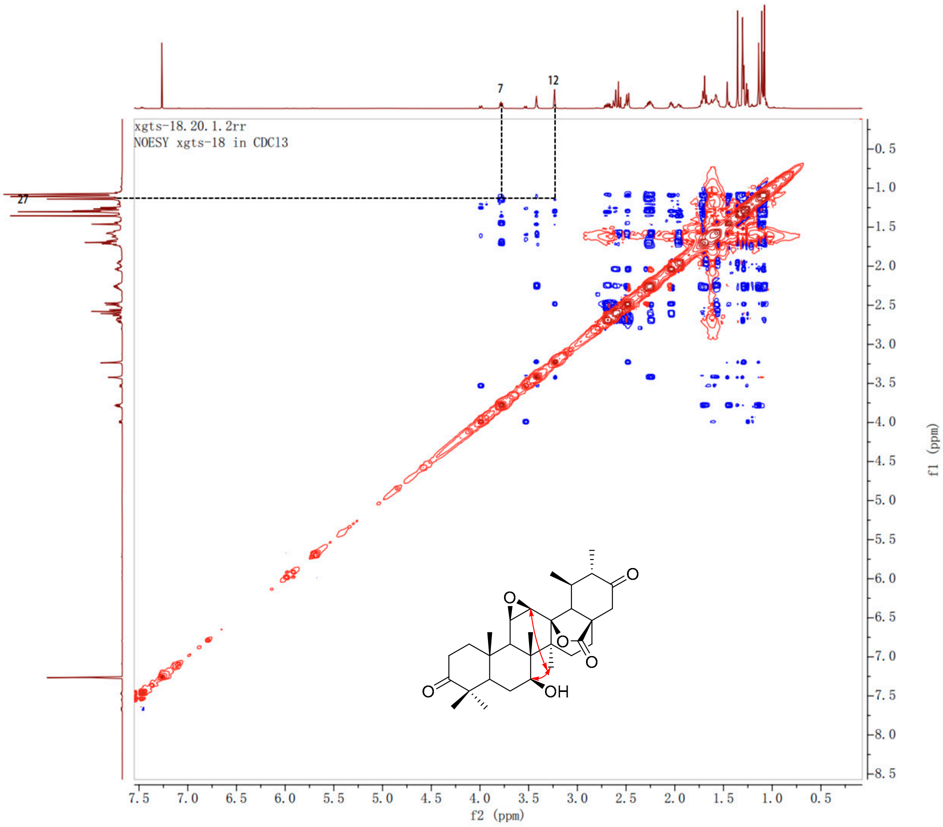


| | Parameter | Value |
|---|---|---|
| 1 | Data File Name | E:/ NMR data/ 17/ NOESY/ pdata/ 1/ 2rr |
| 2 | Title | xgts-18.20.1.2rr |
| 3 | Comment | NOESY xgts-18 in CDCl3 |
| 4 | Origin | Bruker BioSpin GmbH |
| 5 | Owner | nmrsu |
| 6 | Site | |
| 7 | Spectrometer | Avance Neo |
| 8 | Author | |
| 9 | Solvent | CDCl3 |
| 10 | Temperature | 298.0 |
| 11 | Pulse Sequence | noesygpphpp |
| 12 | Experiment | NOESY |
| 13 | Probe | Z167963_0004 (CP2.1 DCH 600S3 C/ H-D-05 Z XT) |
| 14 | Number of Scans | 2 |
| 15 | Receiver Gain | 101 |
| 16 | Relaxation Delay | 2.0000 |
| 17 | Pulse Width | 10.0000 |
| 18 | Presaturation Frequency | |
| 19 | Acquisition Time | 0.1311 |
| 20 | Acquisition Date | 2022-06-15T23:41:34 |
| 21 | Modification Date | 2022-06-15T23:41:10 |
| 22 | Class | |
| 23 | Spectrometer Frequency | (600.13, 600.13) |
| 24 | Spectral Width | (7812.5, 7801.8) |
| 25 | Lowest Frequency | (-317.5, -314.5) |
| 26 | Nucleus | (1H, 1H) |
| 27 | Acquired Size | (1024, 256) |
| 28 | Spectral Size | (1024, 1024) |

**Figure S87** NOESY spectrum (400 MHz) of compound **17** in $CDCl_3$

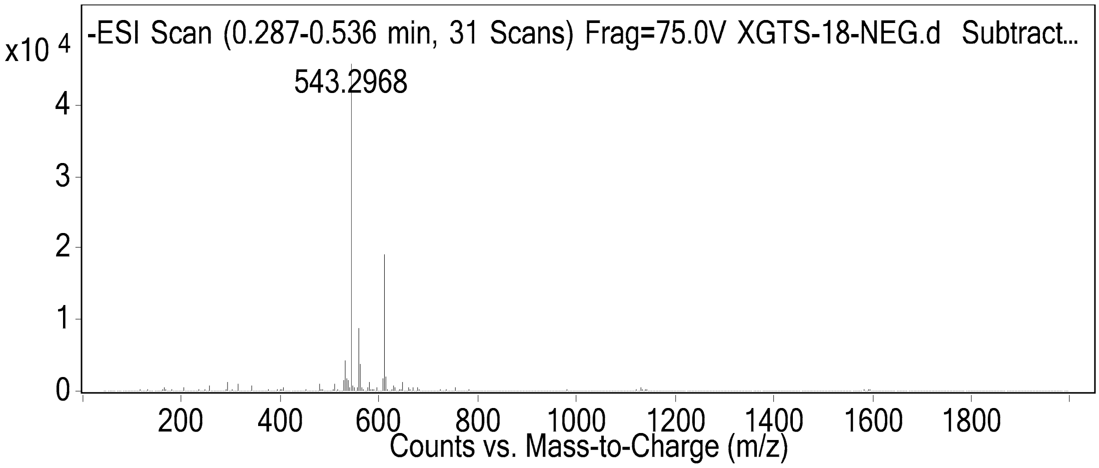


**Figure S88** HR-ESI-MS spectrum of compound **17**

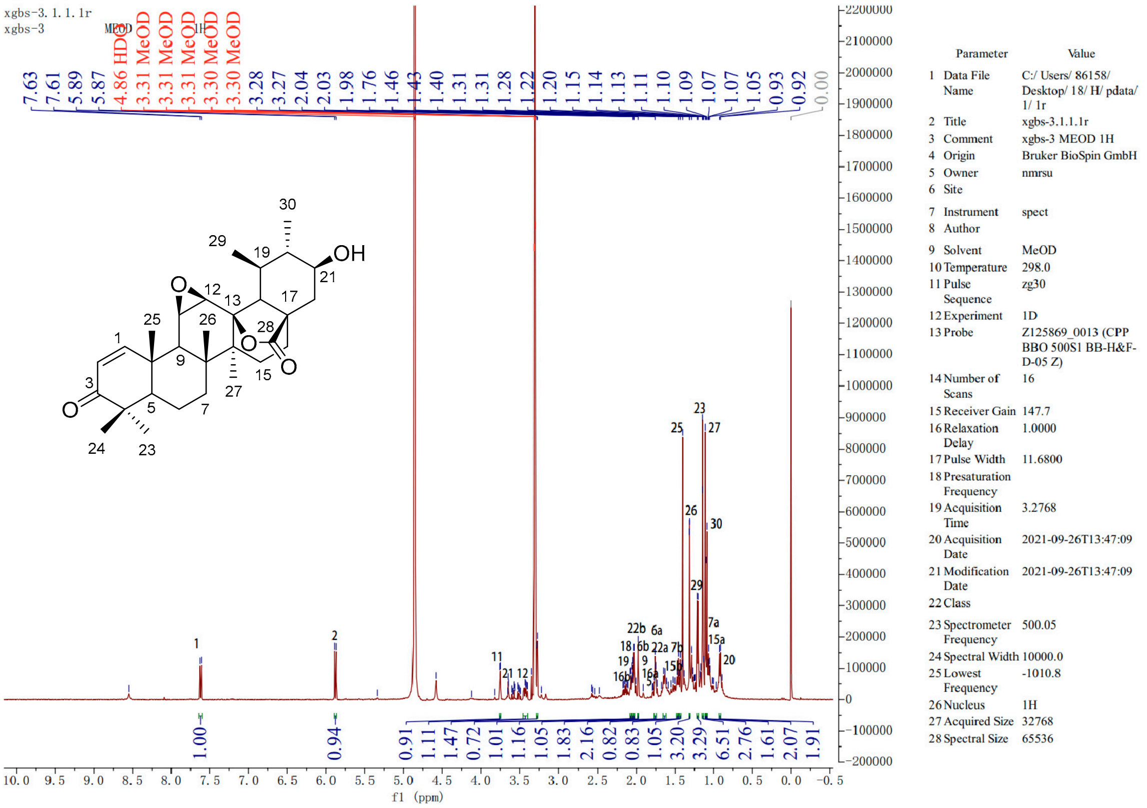

**Figure S89** $^{1}$H NMR spectrum (500 MHz) of compound **18** in $CD_3OD$

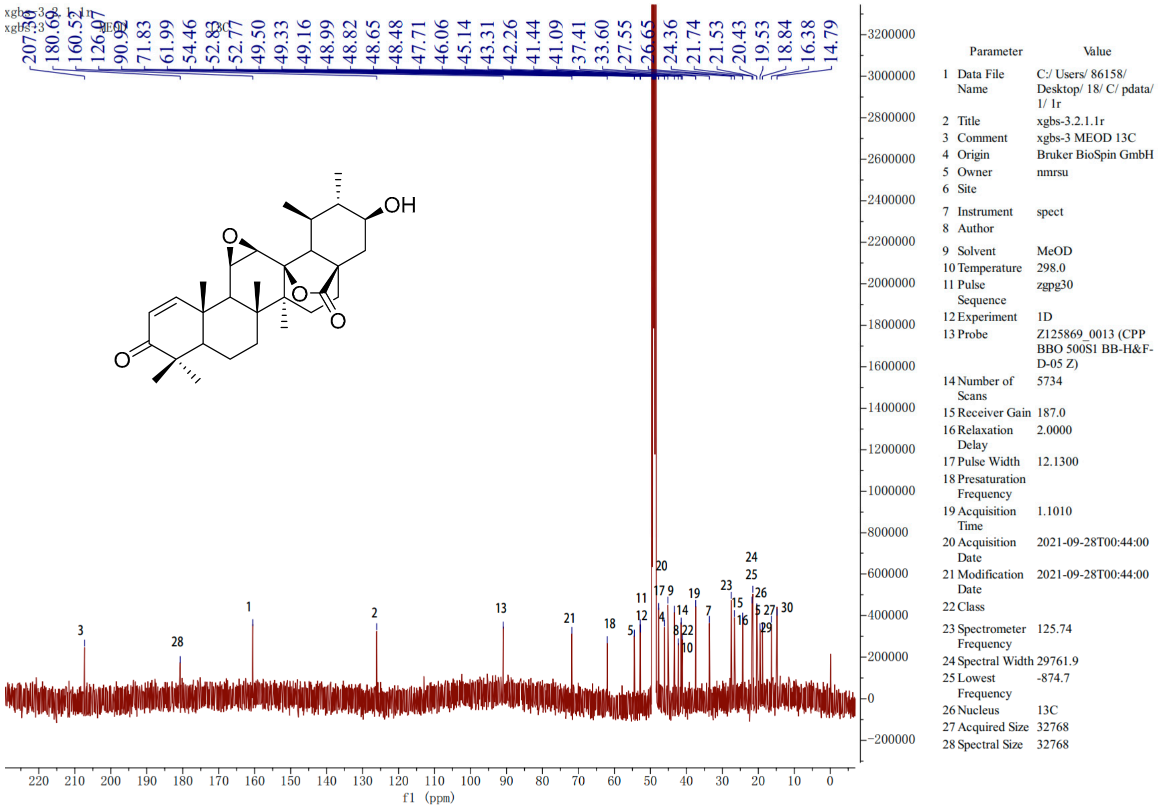

**Figure S90** $^{13}$C NMR spectrum (125 MHz) of compound **18** in $CD_3OD$

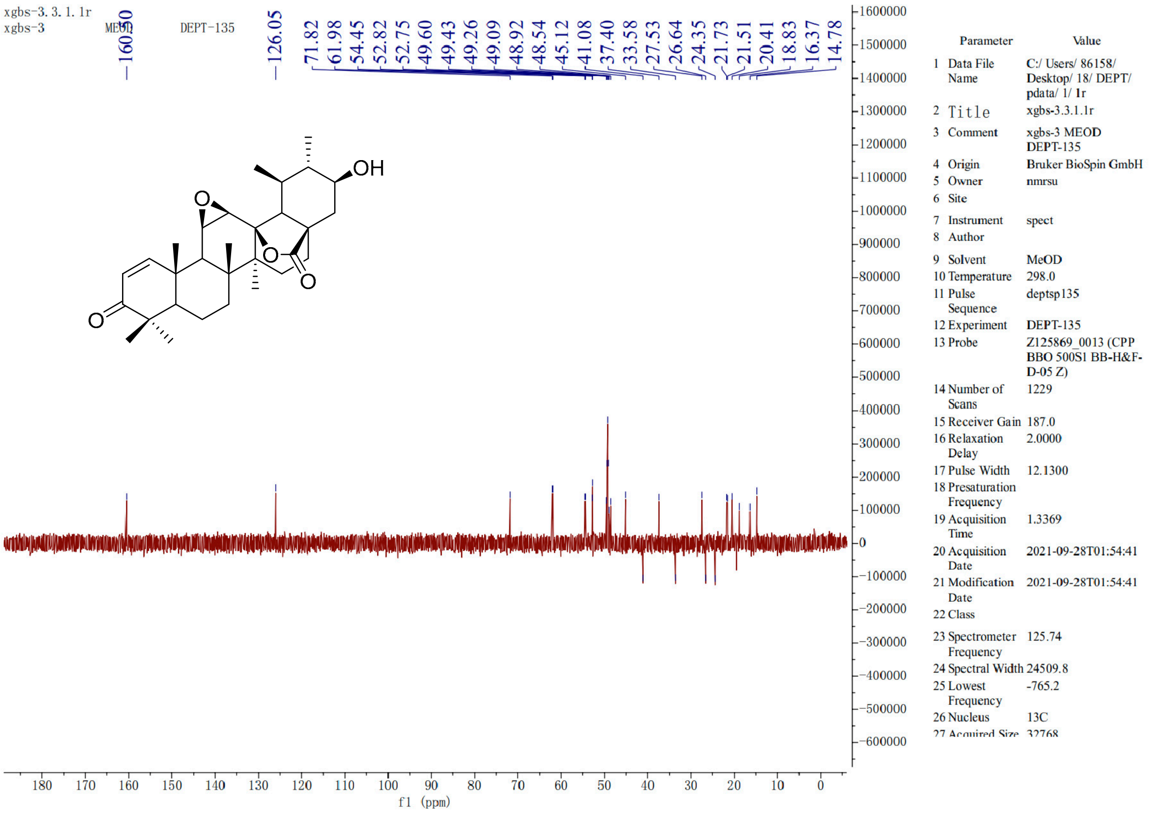

**Figure S91** DEPT-135 spectrum (125 MHz) of compound **18** in $CD_3OD$

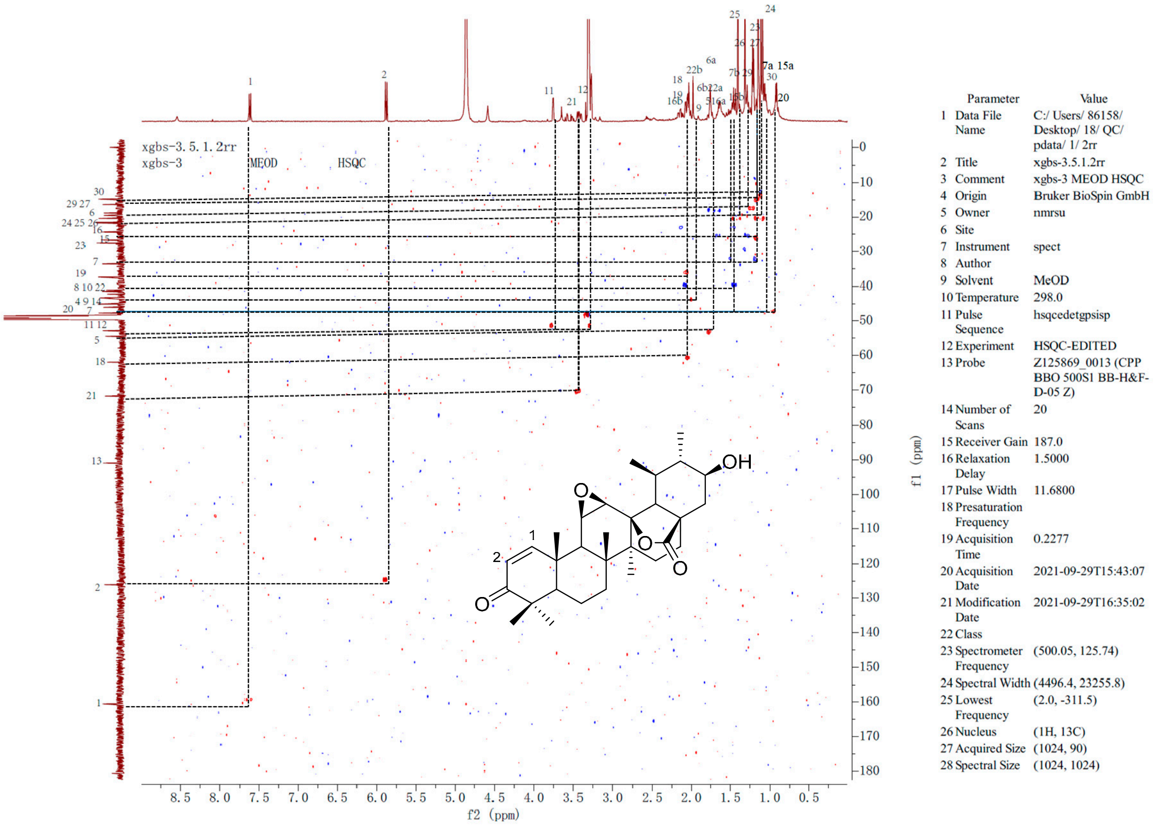

**Figure S92** HSQC spectrum (125 MHz) of compound **18** in $CD_3OD$

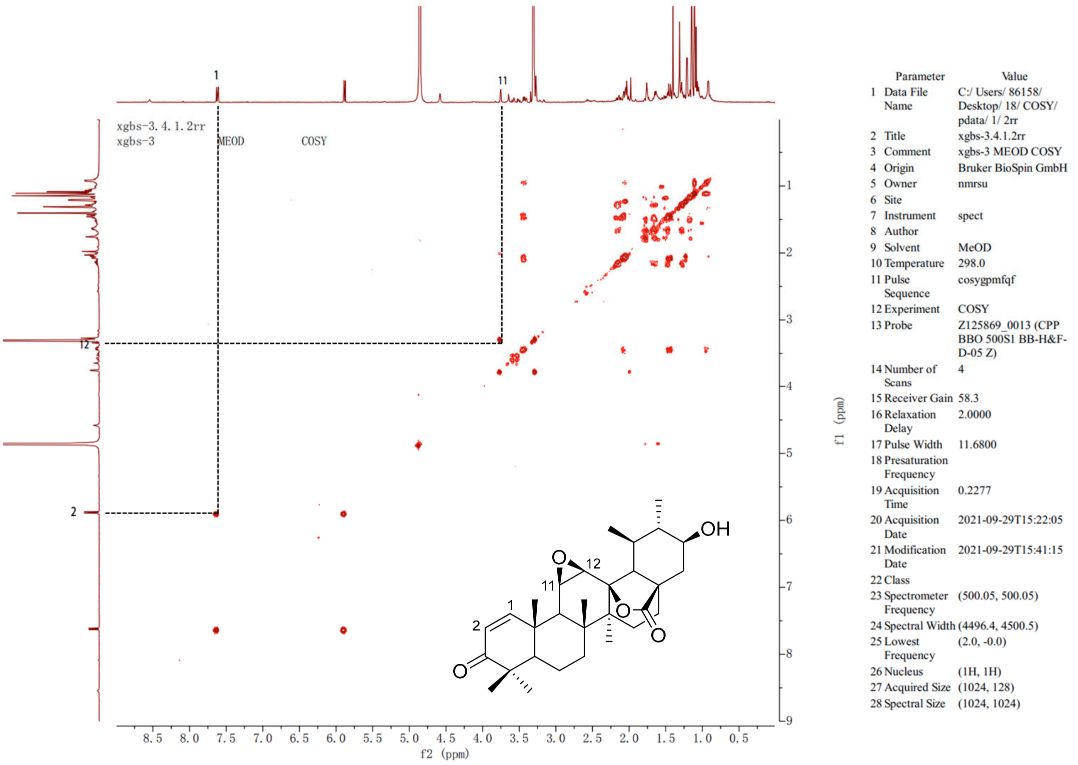


**Figure S93** $^{1}H$-$^{1}H$ COSY spectrum (500 MHz) of compound **18** in $CD_3OD$

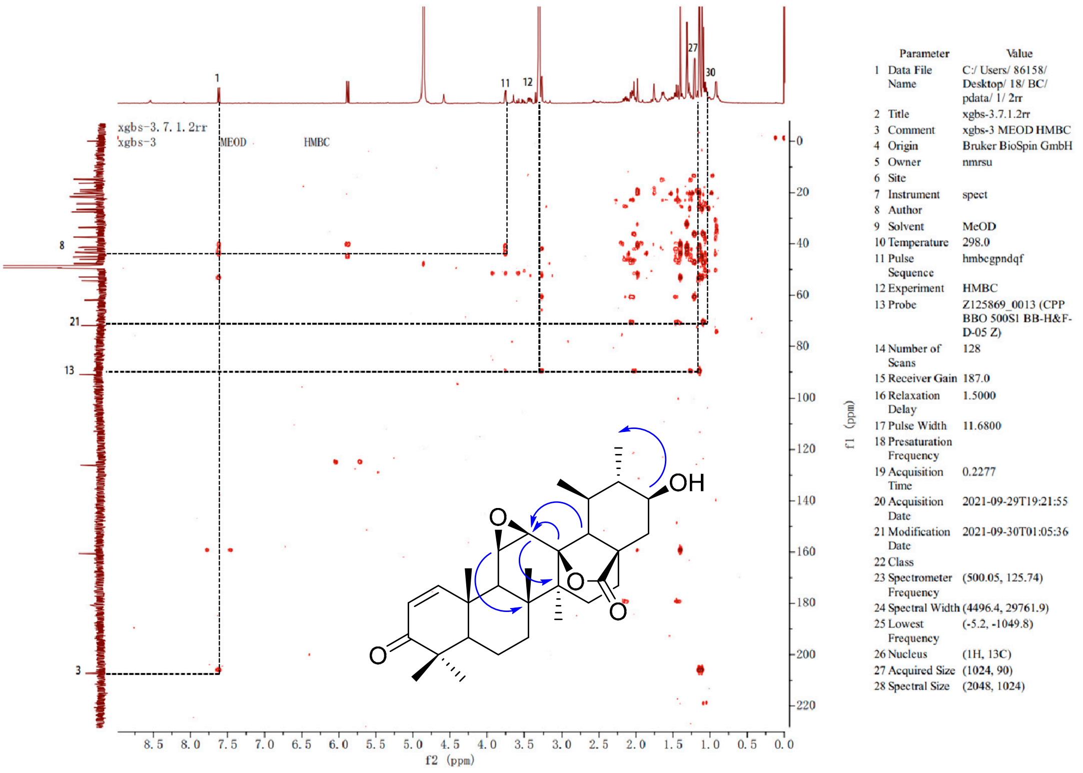


**Figure S94** HMBC spectrum (125 MHz) of compound **18** in $CD_3OD$

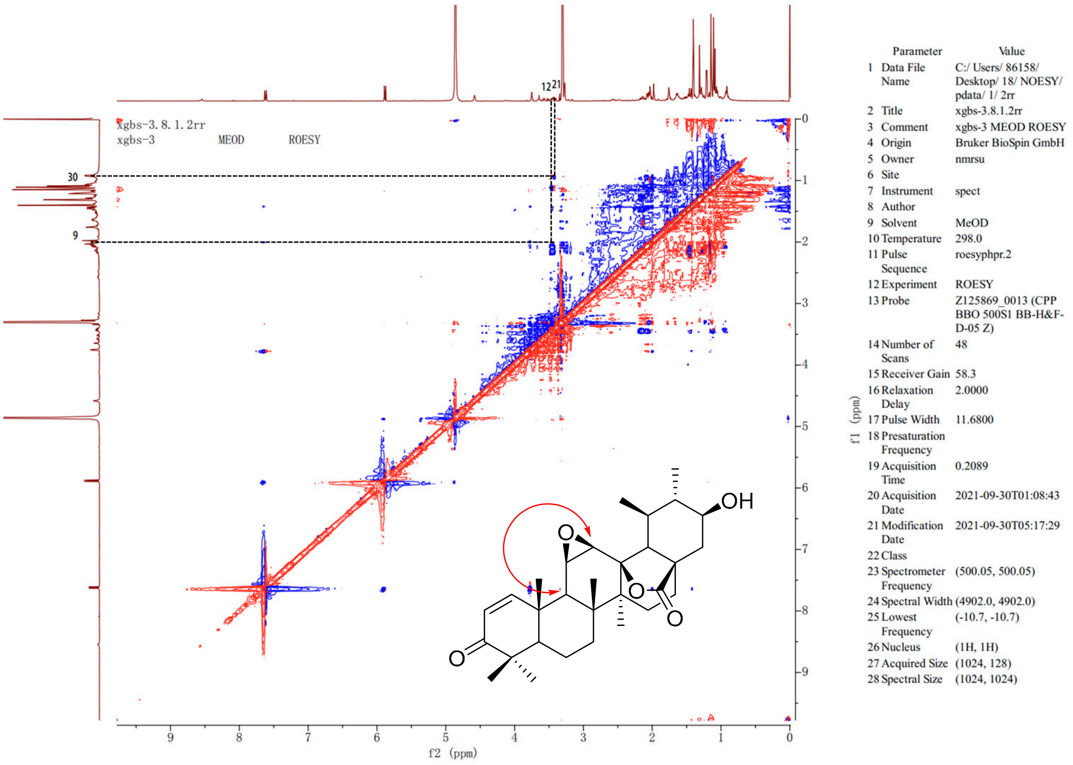


**Figure S95** NOESY spectrum (500 MHz) of compound **18** in $CD_3OD$

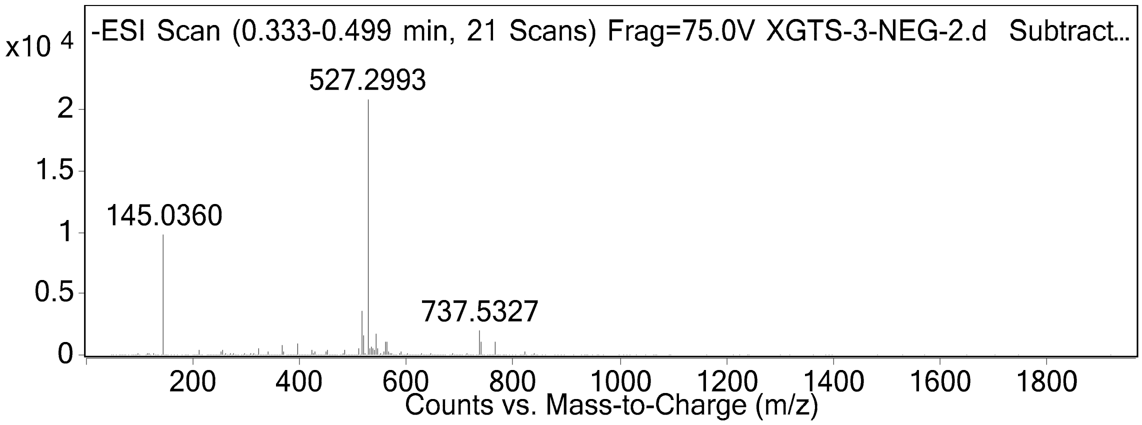


**Figure S96** HR-ESI-MS spectrum of compound **18**

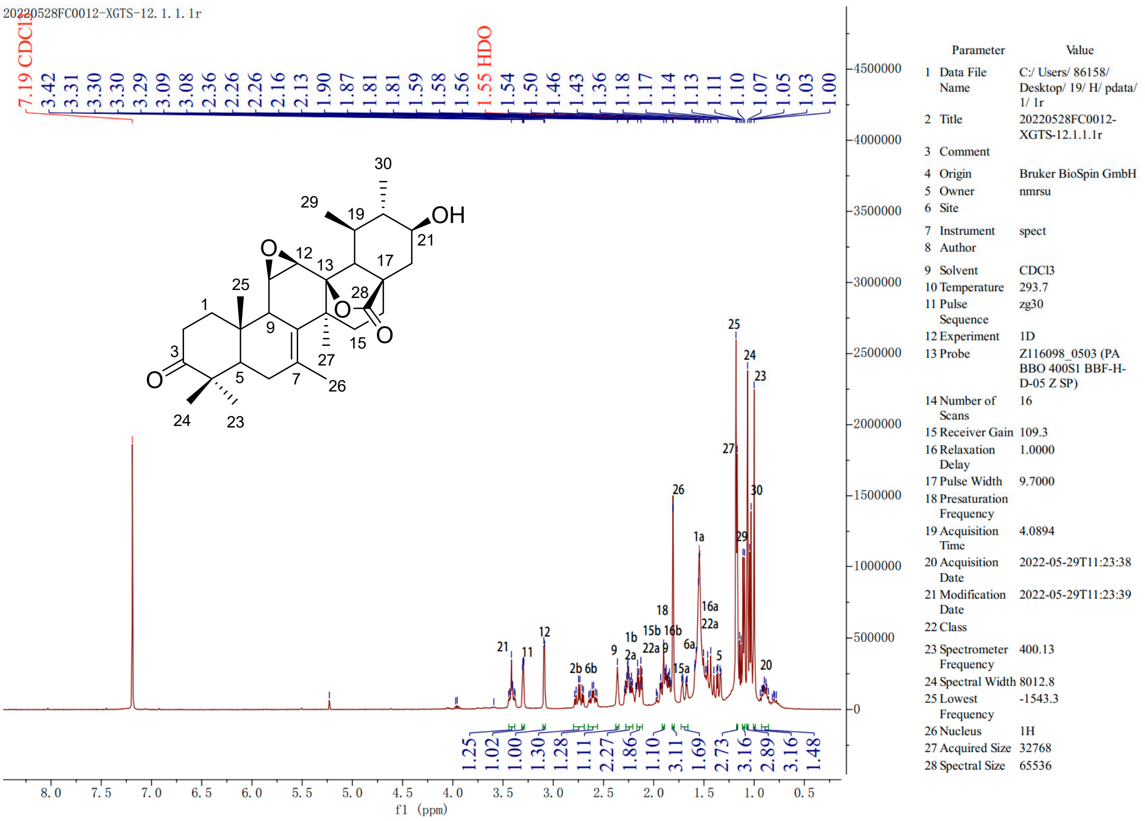


**Figure S97** $^{1}H$ NMR spectrum (400 MHz) of compound **19** in $CDCl_3$

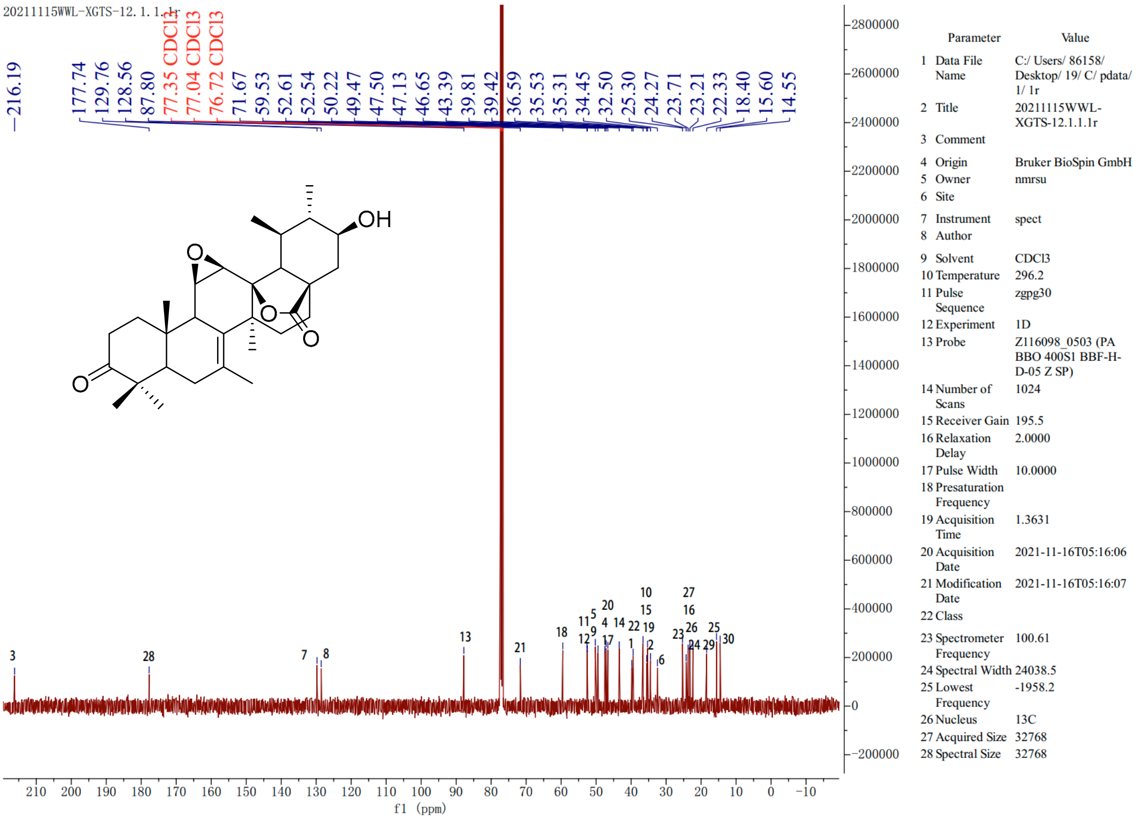


**Figure S98** $^{13}C$ NMR spectrum (100 MHz) of compound **19** in $CDCl_3$

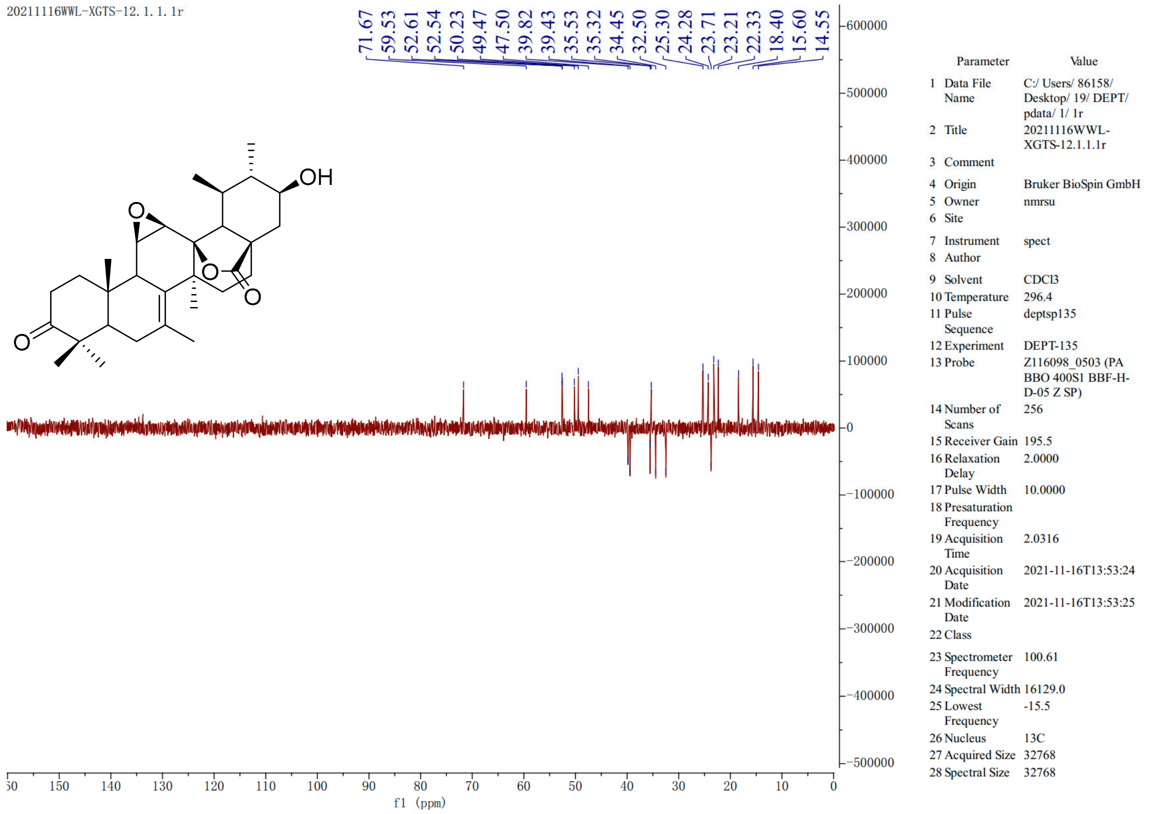


**Figure S99** DEPT-135 spectrum (100 MHz) of compound **19** in $CDCl_3$

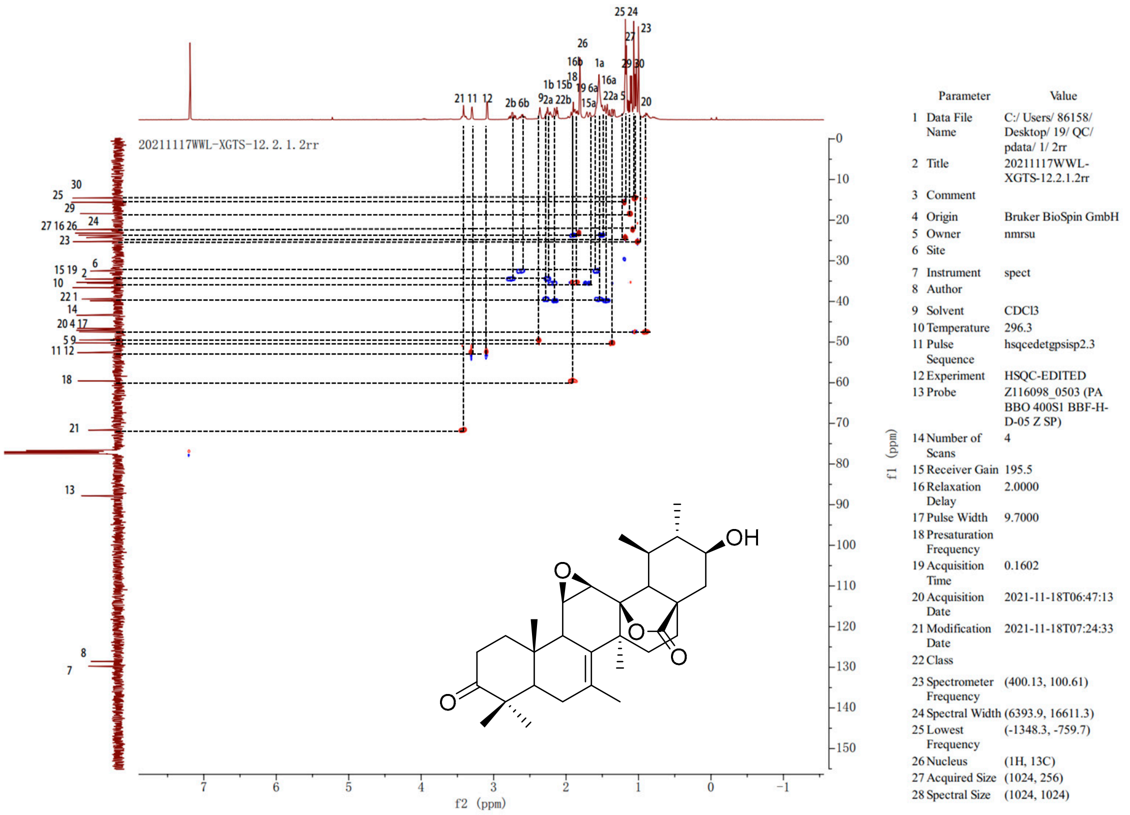


**Figure S100** HSQC spectrum (100 MHz) of compound **19** in $CDCl_3$

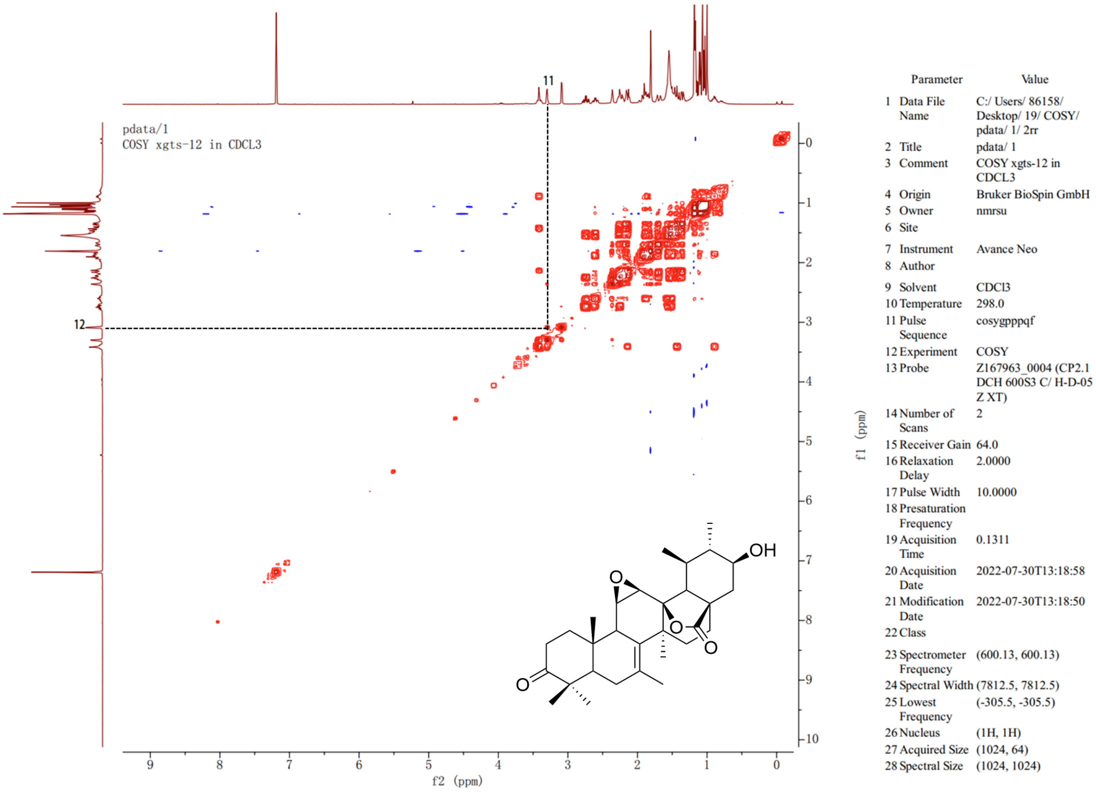


**Figure S101** $^{1}H$-$^{1}H$ COSY spectrum (400 MHz) of compound **19** in $CDCl_3$

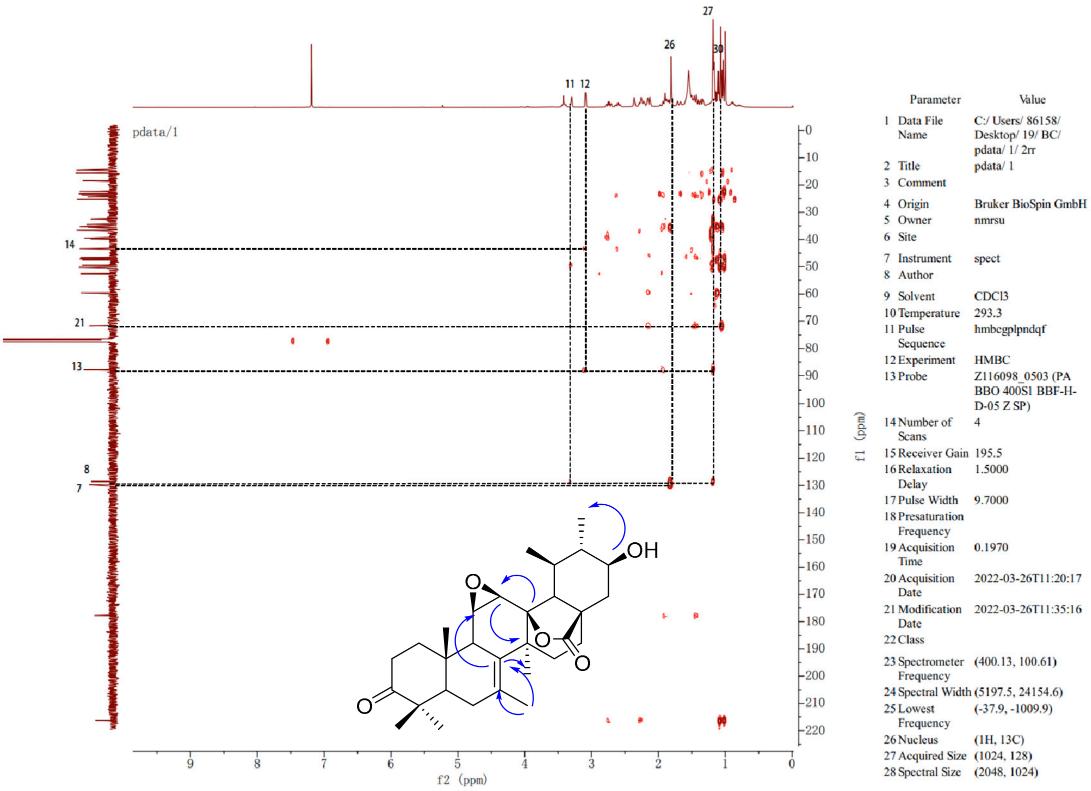


**Figure S102** HMBC spectrum (100 MHz) of compound **19** in $CDCl_3$

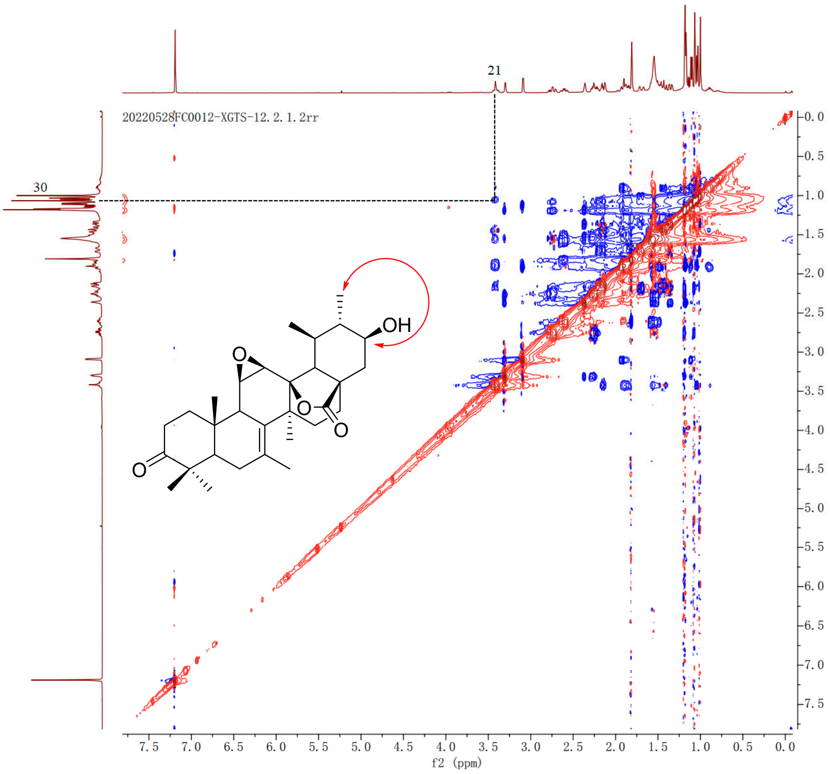


| Parameter | Value |
|---|---|
| 1 Data File Name | C:/ Users/ 86158/ Desktop/ 19/ NOESY/ pdata/ 1/ 2rr |
| 2 Title | 20220528FC0012-XGTS-12.2.1.2rr |
| 3 Comment | |
| 4 Origin | Bruker BioSpin GmbH |
| 5 Owner | nmrsu |
| 6 Site | |
| 7 Instrument | spect |
| 8 Author | |
| 9 Solvent | CDCl3 |
| 10 Temperature | 293.3 |
| 11 Pulse Sequence | noesygpphpp |
| 12 Experiment | NOESY |
| 13 Probe | Z116098_0503 (PA BBO 400S1 BBF-H-D-05 Z SP) |
| 14 Number of Scans | 4 |
| 15 Receiver Gain | 62.3 |
| 16 Relaxation Delay | 1.9324 |
| 17 Pulse Width | 9.7000 |
| 18 Presaturation Frequency | |
| 19 Acquisition Time | 0.3236 |
| 20 Acquisition Date | 2022-05-29T11:25:46 |
| 21 Modification Date | 2022-05-29T12:10:16 |
| 22 Class | |
| 23 Spectrometer Frequency | (400.13, 400.13) |
| 24 Spectral Width | (3164.6, 3164.6) |
| 25 Lowest Frequency | (-11.8, -11.8) |
| 26 Nucleus | (1H, 1H) |
| 27 Acquired Size | (1024, 256) |
| 28 Spectral Size | (1024, 1024) |

**Figure S103** NOESY spectrum (400 MHz) of compound **19** in $CDCl_3$

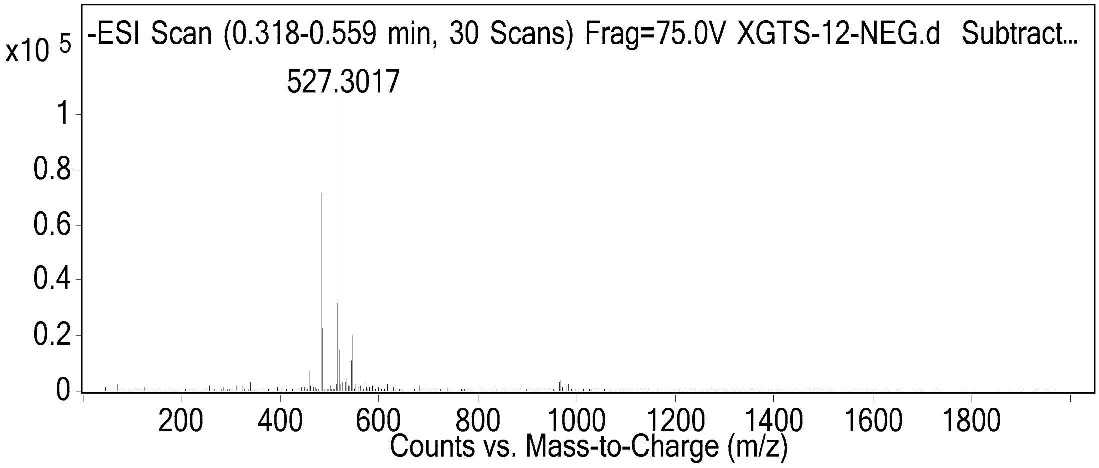


**Figure S104** HR-ESI-MS spectrum of compound **19**
